# Interactions Between Active Matters and Endogenous Fields

**DOI:** 10.1002/adma.202503091

**Published:** 2025-09-07

**Authors:** Jinwei Lin, Qiaoxin Guan, Jiangqi Feng, Shuqin Chen, Leilei Xu, Jianguo Guan, Samuel Sánchez

**Affiliations:** ^1^ Institute for Bioengineering of Catalonia (IBEC) Barcelona Institute of Science and Technology (BIST) Carrer de Baldiri i Reixac, 10‐12 Barcelona 08028 Spain; ^2^ State Key Laboratory of Advanced Technology for Materials Synthesis and Processing International School of Materials Science and Engineering Wuhan University of Technology Wuhan 430070 China; ^3^ Institució Catalana de Recerca i Estudis Avançats (ICREA) Passeig de Lluís Companys, 23 Barcelona 08010 Spain; ^4^ Department of Chemistry and Institute for the Physics of Living Systems University College London London WC1H 0AJ UK

**Keywords:** active matter, collective behavior, endogenous fields, nanomotors

## Abstract

Active matter, encompassing both natural and artificial systems, utilizes environmental energy to sustain autonomous motion, exhibiting unique non‐equilibrium behaviors. Artificial active matter (AAM), such as nano/micromotors, holds transformative potential in precision medicine by enhancing drug delivery and enabling targeted therapeutic interventions. Under the demand for increasing intelligence in AAM, controlling their non‐equilibrium processes within complex in vivo environments presents significant challenges. Endogenous fields—biological fields generated within living systems—play a pivotal role in guiding natural active matter's (NAM) directional migration and collective transformations, offering a strategy for in vivo control of non‐equilibrium systems. Research in NAMs‐inspired AAMs spans biology, chemistry, materials science, engineering, and physics, yet communication barriers among disciplines often impede progress. This review seeks to bridge these gaps by summarizing the key characteristics of chemical and physical endogenous fields in biological contexts such as tumors, wounds, and inflammation. It explores how natural and artificial active matter sense, transmit, and execute responses to these fields, and discusses how insights from natural systems can inform the design of synthetic counterparts. Potential issues and prospects of this research direction are also discussed. It is hoped that this review fosters interdisciplinary collaborations and propels the development of intelligent active matter for biomedical applications.

## Graphical abstract

This review synthesizes endogenous field information and computational methods in contexts such as cancer, wounds, and biofilms. It organizes NAMs and AAMs by sensing, transmitting, and executing functions, compares their limitations, and from these contrasts proposes design strategies for next‐generation AAMs, offering perspectives to foster interdisciplinary dialogue and future research.

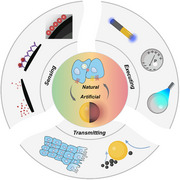

## 1 Introduction

Over the past two decades, research on active matter has expanded significantly, focusing on systems composed of colloidal particles or microorganisms that consume energy and generate forces, thereby transcending the constraints of equilibrium statistical mechanics.^[^ ^1^, ^2^, ^3^, ^4^ ^]^ Broadly, these systems can be categorized into natural active matter (NAM), such as proteins, bacteria, algae, and various cells with distinctive propulsion mechanisms and metabolic dynamics,^[^ ^5^ ^]^ and artificial active matter (AAM), which are synthetic, non‐living entities often referred to as micro/nanorobots or micro/nanomotors. AAMs mimic the motion and collective behaviors of their natural counterparts^[^ ^6^ ^]^ and have garnered significant attention for their potential in fields such as biomedicine^[^ ^7^, ^8^, ^9^ ^]^ environmental remediation,^[^ ^10^, ^11^ ^]^ and microscale manipulation.^[^ ^12^, ^13^, ^14^, ^15^, ^16^ ^]^

One of the most promising applications of AAMs lies in precision medicine. The concept of nanomachines navigating through blood vessels to target cancer cells, popularized by works of fiction—like *Fantastic Voyage*, remains an enduring inspiration despite its frequent use as a cliché. The first generation of AAMs has demonstrated their ability to localize therapeutic interventions, often guided by external magnetic fields,^[^ ^17^, ^18^, ^19^ ^]^ and to enhance the penetration and distribution of therapeutic agents in specific lesions by exploiting non‐equilibrium diffusion.^[^ ^20^, ^21^ ^]^ For instance, the enhanced diffusion of NO‐powered nanomotors, inspired by endogenous biochemical reactions, improves their cell uptake and offers beneficial effects, such as promoting endothelialization and exhibiting anticancer properties.^[^ ^22^ ^]^ Furthermore, swarms of radio‐labeled nanomotors have demonstrated an eight‐fold increase in tumor penetration, leading to ≈90% reduction in tumor size through radionuclide therapy.^[^ ^9^ ^]^ As the field advances, the need for “intelligence” in AAMs has emerged, which is a capability to adaptively control non‐equilibrium processes in response to changing environments. However, the microscale and nanoscale dimensions of AAMs present significant challenges to integrating conventional chips, the traditional carriers of computational intelligence. This raises a pivotal question: how can intelligence be embedded in active matter without relying on chips?

In nature, NAMs offer valuable insights for this, especially through their interactions with “endogenous fields”—fields generated within biological systems rather than imposed externally. Despite lacking computational systems akin to chips or brains, nano‐ and microscale NAMs exhibit remarkable adaptability to their environments and execute complex tasks. For example, neutrophils dynamically sense and respond to a range of physicochemical cues, including IL‐8, CXCL1, and reactive oxygen species (ROS), enabling them to exit the bloodstream and infiltrate inflamed tissues.^[^ ^23^ ^]^ Endogenous electric field guides the epidermal cells to move to the cathode by the migration of membrane components.^[^ ^24^ ^]^ These observations underscore the potential of NAMs as a blueprint for creating the next generation of intelligent AAMs. Key questions arise from: 1) What are the intensity, direction, and spatial characteristics of endogenous fields? 2) How do NAMs and AAMs interact with these fields? 3) What specific strategies from NAMs can inspire AAM development? Answering these questions will demand interdisciplinary collaboration across physics, materials science, chemistry, engineering, and biology. AAM design must integrate physiological data from biomedical studies on endogenous fields and insights into NAM behaviors from biophysical and biochemical research, ultimately translating these principles into tangible materials and mechanical designs. However, this interdisciplinary approach also introduces communication challenges, underscoring the need for a common language and comprehensive summaries on endogenous fields and their interactions with both NAMs and AAMs. Such efforts will establish a foundational understanding for the field and illuminate pathways for future research.

In this review, we begin by providing an overview of active matter and its fundamental propulsion principles (Section 2), followed by an introduction to computational methods employed in the study of fields (Section 3). Section 4 compiles a detailed summary of ten endogenous fields, including oxygen gradients, pH variations, ROS, topological structures, and electric fields, among others, which are particularly relevant to NAMs’ applications in contexts such as tumors, skin, and inflammation (summarized in Tables 1 and 3). Section 5 delves into the interactions between NAMs, AAMs, and fields, with an emphasis on mechanisms, models, and in vitro studies (highlighted in Tables 4, 5, 6). Finally, we compare NAMs and AAMs, examining how NAM‐inspired designs could inform the development of advanced AAMs. This discussion also emphasizes unresolved challenges, particularly in chemotaxis, a key focus area in the field. Some perspectives here are subjective, such as outlook views, aiming to stimulate critical discourse and guide future research directions.

**Table 1:** Summary of endogenous chemical fields.

Classification	Position	Intensity	Direction	Method
O_2_	Cervical cancer	12.95–20.7 mmHg cm^−1 [^ ^99^, ^100^, ^101^, ^102^, ^103^, ^104^ ^]^	Outward from the center of cancer	O_2_‐sensitive polarographic needle electrode
	Breast cancer	11.7–21.1 mmHg cm^−1 [^ ^105^, ^106^, ^107^, ^108^ ^]^		
	Prostate cancer	0.97–5.31 mmHg cm^−1 [^ ^109^, ^110^ ^]^		
	Bladder cancer	≈6.21 µm cm^−1[^ ^111^ ^]^		
	Renal cell carcinoma	≈3.53 mmHg cm^−1 [^ ^112^ ^]^		^18^F‐fluoromisonidazole (^18^F‐FMISO) positron emission tomography (PET)
	Liver cancer	≈8.3 mmHg cm^−1 [^ ^113^, ^114^ ^]^		O_2_‐sensitive polarographic needle electrode
	Biofilm	≈0.1625 mM µm^−1[^ ^83^ ^]^ ≈0.15 mM µm^−1[^ ^77^ ^]^ (*P. aeruginosa*)	Toward the center of biofilm	
	Intestine	79–100 mmHg cm^−1[^ ^82^, ^115^, ^116^ ^]^ (large intestine) 50–90 mmHg cm^−1[^ ^117^, ^118^ ^]^	Outward the mucosa	Phosphorescence quenching method by oxygen probe
	Kidney	≈1 mmHg cm^−1[^ ^119^ ^]^	Outward the medulla	O_2_‐sensitive polarographic needle electrode
	Arthrosis	≈2.5 % mm^−1[^ ^75^, ^76^ ^]^ ≈1 % mm^−1[^ ^75^, ^76^ ^]^	Toward the calcified cartilage Toward the “middle zone”	
	Wound	Acute wounds: ≈70 mmHg cm^−1[^ ^72^ ^]^ Chronic wounds: ≈40.8 mmHg cm^−1^	Toward the wound boundary	Luminescent indicator dye
pH	Lymphoma cancer (rat)	0.07–0.19 mm^−1[^ ^120^, ^121^ ^]^	Outward from the center of cancer	^13^C‐labelled magnetic resonance spectroscopy (MRS)^[^ ^121^ ^]^ ^13^C‐labelled Z‐OMPD chemical shift imaging (CSI)^[^ ^120^ ^]^
	Breast cancer	5.6–7.5 mm^−3[^ ^122^, ^123^, ^124^ ^]^		^31^P MRS of pi and 3‐APP^[^ ^123^, ^124^ ^]^ ^1^H MRS of IEPA^[^ ^122^ ^]^
	Colon cancer	≈0.25 cm^−1 [^ ^125^ ^]^		^31^P MRS of pi and 3‐APP
	Sarcoma cancer (rat)	≈0.15 mm^−1[^ ^126^ ^]^		
	Hepatoma cancer (rat)	≈0.1 mm^−1[^ ^127^ ^]^		^19^F MRS of ZK‐150471
	Adenocarcinoma cancer (rat)	≈0.11 mm^−1[^ ^128^, ^129^ ^]^		^13^C‐labelled zymonic acid nuclear magnetic resonance (NMR)
	Healthy bladder (rat)	≈1.23 cm^−1[^ ^129^ ^]^	Toward the cortex	
	Healthy kidneys (rat)	≈0.52 cm^−1[^ ^120^, ^129^ ^]^	Toward the medulla	^13^C‐labelled zymonic acid nuclear magnetic resonance (NMR)^[^ ^129^ ^]^ ^13^C‐labelled Z‐OMPD chemical shift imaging (CSI)^[^ ^120^ ^]^
	Wound (rat)	3–6 mm^−1[^ ^72^, ^85^ ^]^ ≈1.5 cm^−1^ (chronic wound)^[^ ^130^ ^]^	Outward from the center of the wound	Luminescent indicator dye
	Biofilm	≈0.1 µm^−1^ (*P. fluorescens*)^[^ ^87^, ^131^ ^]^ 0.72 mm^−1^ (*E. coli* PHL628)^[^ ^132^ ^]^	Outward from the center of the biofilm	
	Stomach (pig)	≈8 mm^−1^ (in the mucus of the guinea pig)^[^ ^133^, ^134^ ^]^	Toward the bottom of the mucus	Microelectrode
	Gut	≈13 mm^−1[^ ^86^, ^135^ ^]^	Toward the microvilli	
ROS	Malignant cancer (no specific position)	cH_2_O_2_: ≈66.7 µM mm^−1[^ ^70^ ^]^	Toward the center of cancer	–
	Prostate carcinoma (*ex vivo*)	cH_2_O_2_: 50–100 µM µm^−1[^ ^136^ ^]^		Nanoelectrode‐based electrochemical method
	Osteoarthritis	cROS: 500–1000 µM µm^−1[^ ^91^ ^]^	Toward the cartilage	Chemiluminescence
	Wound (zebrafish)	H_2_O_2_ gradient (without specific value)^[^ ^137^ ^]^	Toward the center of the wound	
	Lung	cH_2_O_2_: < 0.6 µM mm^−1^ (exhaled breath condensate)^[^ ^95^, ^96^ ^]^ cH_2_O_2_: 0.13–1.5 µM mm^−1^ (smoker)^[^ ^138^ ^]^ cH_2_O_2_: 0.1–0.47 µM mm^−1^ (bronchoalveolar lavage fluid in rat)^[^ ^139^, ^140^, ^141^ ^]^	Inward the lung	Electron paramagnetic resonance (EPR) spectroscopy^[^ ^95^ ^]^ Fluorometric method^[^ ^95^, ^96^, ^139^, ^140^, ^141^ ^]^
	Kidney	cH_2_O_2_: 100 ± 60 µM (fresh urine)^[^ ^142^, ^143^, ^144^ ^]^	–	Fluorometric method

Note: In the above table, the field intensity calculations (except for those related to ROS) are all performed using *method 1*, and the value of *dx* is derived from the actual tumor/tissue size. The given ranges are statistics of medium value from multiple references, the rest are the measured medium values or specific values of a single reference.

## 2 Brief Introduction of Active Matter

Active matter encompasses systems of self‐propelled entities far from equilibrium. These entities extract energy from their surroundings or intrinsic stores, enabling them to move autonomously, thereby producing complex dynamic behaviors and emergent macroscopic properties. Broadly, active matter systems are categorized as either natural or artificial, based on their sources. In this review, we focus primarily on active matter at the nano‐ and microscale, where fluid‐mediated motion poses unique challenges due to the absence of inertial forces, such as, in an overdamped regime governed by the Stokes Equation:^[^ ^25^ ^]^

(1) $$-\nabla p+\mu \nabla^{2}u=0,\;\;\;\;\;\;\nabla \cdot u=0$$

where *p*, μ, **u** are pressure, dynamic viscosity, and velocity of fluid. The Stokes equation's linearity, immediacy, and time‐reversibility imply a strict linear relationship between kinetics and kinematics, necessitating a continuous net force acting on active matter for sustained movement. It implies the critical role of building asymmetry in enabling self‐propulsion. In the following section, we outline the primary propulsion principles for both NAMs and AAMs, with view of building asymmetry around active matters.

### 2.1 Natural Active Matter

#### 2.1.1 Natural Molecular Motor

**Figure 1:**
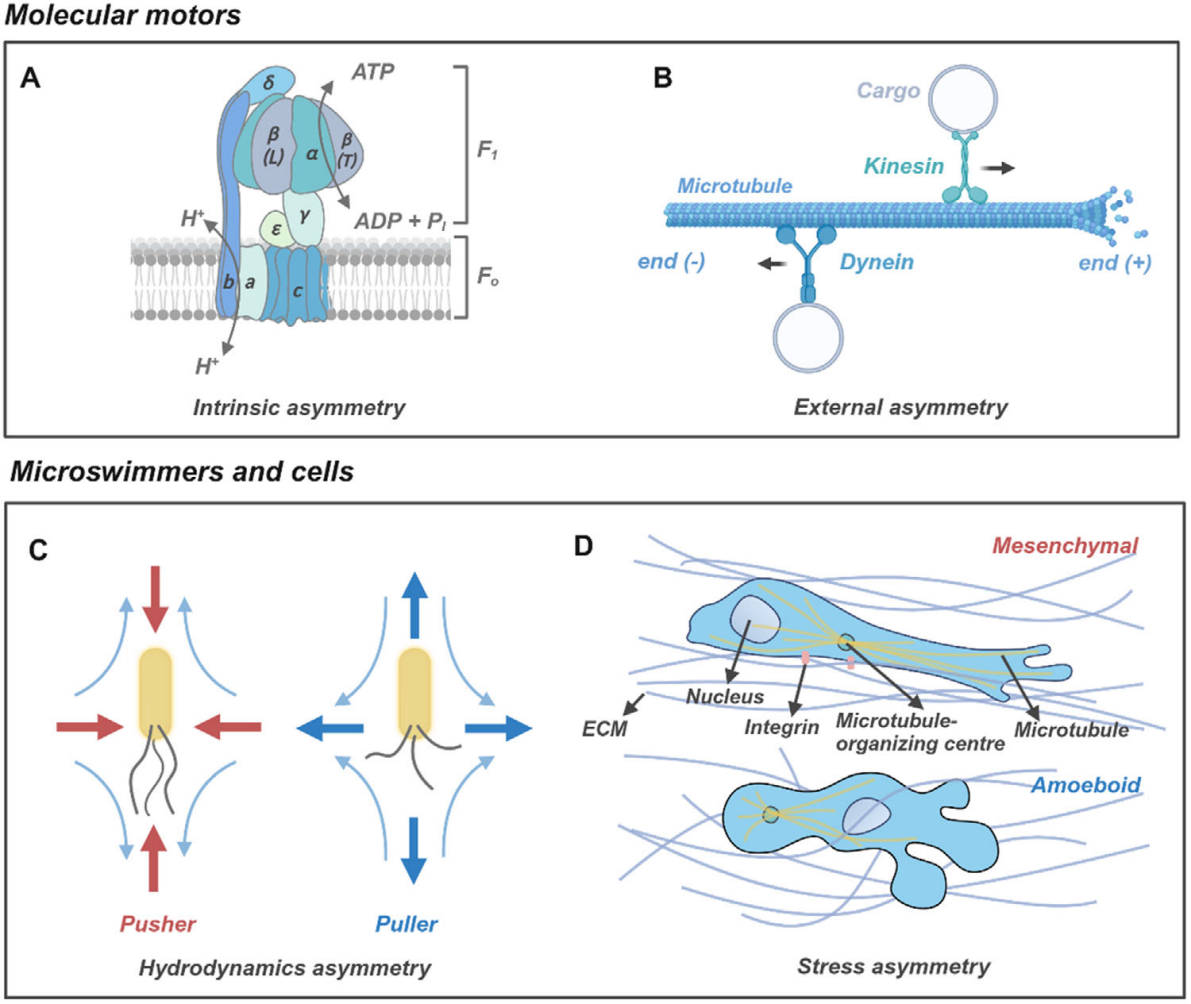
Schematic illustration of propulsion principles for NAMs. A) Structure of FoF1‐ATP synthase: two rotary molecular motors attached to a common shaft, each attempting to rotate in opposite directions. The proton channel lies at the interface between subunits a and c. B) Stepping movement of kinesin and dynein on polarized microtubule, where kinesin moves toward the positive end (end (+)) and dynein moves toward the negative end (end (−)). C) “Pusher” and “puller” types of microswimmers build asymmetric flow around them. D) Mesenchymal and amoeboid cell migration provided by stress asymmetry.

Natural molecular motors, protein‐based nanomachines integral to cellular processes, convert energy into asymmetric mechanical deformation to drive essential biological functions. This class of motors includes myosins, kinesins, dyneins, DNA and RNA polymerases, and adenosine triphosphate (ATP) synthase, which can be broadly classified into two categories: linear and rotary motors.^[^ ^26^ ^]^ Activation of these motors predominantly relies on ATP hydrolysis or the utilization of transmembrane proton gradients. Provided that the free energy released from ATP hydrolysis (≈12 kcal mol^−1^) exceeds the product of the mechanical load and step size, adjusted for motor efficiency, these biomolecular systems can sustain continuous cycles of mechanochemical reactions. One mechanism by which molecular motors achieve directional motion is through intrinsic asymmetry in molecular structure and the establishment of chemical gradients. For example, the rotary FoF1‐ATP synthase complex comprises two primary domains: the membrane‐embedded Fo sector, facilitating proton translocation, and the cytoplasmic F1 sector, which houses catalytic sites for ATP synthesis.^[^ ^27^ ^]^ Within the F1 sector, the β subunits transition through three conformational states—relaxed (L), tense (T), and open (O)—that coordinate ATP synthesis through a periodic cycle. In its hydrolytic mode, the enzyme rotates in 120° left‐handed increments, with each rotation driven by the hydrolysis of a single ATP molecule. Conversely, in the synthetic mode, the F1 head rotates 120° right‐handed, coupling proton gradients to ATP production (**Figure** **1**A). External asymmetry can also dictate directional motion, as demonstrated by linear motors like myosin and kinesin. Myosins facilitate muscle contraction, while kinesins “walk” along polar cytoskeletal filaments such as actin and microtubules (Figure 1B).^[^ ^28^, ^29^ ^]^ Microtubules, which provide structural support and serve as tracks for motor proteins, are polar filaments composed of tubulin subunits that determine the binding and movement direction of kinesins and dyneins.^[^ ^29^, ^30^ ^]^ These filaments exhibit a fast‐growing “plus” end and a slow‐growing “minus” end. Kinesins transport cargo from the “minus” to the “plus” end, extending to the cell periphery, whereas dyneins move in the opposite direction, toward the “minus” end. This bidirectional transport is critical for maintaining intracellular organization and overcoming obstacles such as viscosity and Brownian motion.

#### 2.1.2 Microswimmers and Cells

The rotational and linear mechanical motions generated by molecular motors underpin the autonomous movements of microswimmers and cells, driving phenomena such as flagellar motion, cellular deformation, migration, and collective behaviors. Both microswimmers and cells achieve motion by creating asymmetric fluid flows and stress distribution around them.

In microswimmers, propulsion primarily arises from the coordinated movement of flagella or cilia, a mechanism shared by diverse organisms, including bacteria, fungi, algae, and sperm. Depending on the species, microswimmers may possess one or multiple flagella positioned at various surface locations. Each flagellum is powered by a molecular motor embedded in the cell membrane, generating hydrodynamic flows that propel the microswimmer forward. These swimming modes are broadly classified as “puller” or “pusher” types, based on their far‐field hydrodynamic signatures (Figure 1C). For example, flagellated bacteria often exhibit a run‐and‐tumble motion, alternating between linear swimming and brief reorientation phases to adjust their trajectories.^[^ ^31^ ^]^

Cellular migration, in contrast, involves dynamic cell shape remodeling and interactions with the extracellular matrix (ECM). Single‐cell migration is typically categorized into two distinct modes: amoeboid and mesenchymal, which differ in morphology and function (Figure 1D). Amoeboid migration, exemplified by the movement of *Dictyostelium discoideum*,^[^ ^32^ ^]^ proceeds via a cyclical process: cortical layer rupture (separation of the membrane from the cortex), cytoplasmic flow to form protrusions, cortical healing, and cell body contraction.^[^ ^33^, ^34^ ^]^ This mechanism, driven by reorganization of the cortical actin cytoskeleton, enables rapid repositioning (2–30 µm min^−1^) with minimal ECM degradation, reflecting weak cell‐ECM interactions. In mesenchymal migration, cells align along the tension lines of anteriorly oriented ECM fibers, leaving behind proteolytically degraded tunnels. This mode is characterized by integrin‐mediated adhesion and follows a sequential process: forward protrusion, adhesion formation, ECM fiber proteolysis, and rear retraction. Mesenchymal cells rely on protease‐loaded vesicles targeted to the leading edge, remodeling the ECM to create migratory pathways.^[^ ^35^ ^]^ Unlike amoeboid cells, mesenchymal cells exhibit strong cell‐ECM interactions and lower migration rates, a feature typical of large adherent cells such as smooth muscle cells, fibroblasts, and gliomas.^[^ ^36^ ^]^ While amoeboid migration relies on squeezing through ECM pores without aligning fibers or proteolytic remodeling, mesenchymal migration depends on contractile forces and ECM digestion. The spatial positioning of the microtubule‐organizing center further differentiates the two modes: anterior to the nucleus in mesenchymal cells and posterior to the nucleus in amoeboid cells.^[^ ^37^ ^]^ These distinct migration strategies highlight the adaptability of cellular systems to diverse mechanical and biochemical environments.

### 2.2 Artificial Active Matter

#### 2.2.1 Artificial Molecular Motors

Artificial molecular motors are synthetic molecular‐level systems designed to provide new insights into the workings of natural molecular motors in biological systems, enabling bottom‐up approaches to understanding their mechanisms. By harnessing external energy sources, these motors undergo conformational changes or interact with substrates, driving motion through the generation of asymmetric stress fields. A notable example is the Feringa motor, developed by Nobel laureate Bernard L. Feringa and his team. These motors utilize large steric alkenes to achieve unidirectional motion on metal surfaces via electric‐field‐induced double‐bond isomerization and vibrationally excited helical inversion.^[^ ^38^ ^]^ Another prominent class of artificial molecular motors includes rotaxanes and catenanes, which are based on mechanically interlocked molecules and exhibit a rich repertoire of conformational motions such as extension, rotation, sliding, and twisting. Rotaxanes consist of a dumbbell‐shaped “axle” threaded through a ring‐like “macrocycle” or “wheel,” with stoppers at both ends of the axle preventing the macrocycle from detaching. In contrast, catenanes comprise two or more interlocked macrocycles mechanically linked without any covalent bonds between them.^[^ ^39^ ^]^ Adsorbate motors represent an emerging class of artificial molecular motors that exploit surface interactions to achieve directed movement. These motors rely on simple molecules, which, when subjected to external energy inputs such as voltage pulses, undergo processes like proton transfer that propel them in specific directions along the surface.^[^ ^40^ ^]^ More recently, DNA origami–based motors have been developed, including bipedal walkers achieving autonomous, high‐speed locomotion on origami tracks^[^ ^41^ ^]^ and rotary ratchet motors capable of processive rotation under electric fields.^[^ ^42^ ^]^ These designs offer precise motion control, excellent biocompatibility, and potential for chip‐independent nanorobotics. The design and functionality of artificial molecular motors offer valuable platforms for mimicking and extending the capabilities of natural molecular motors, bridging the gap between synthetic and biological systems while opening new avenues for fundamental research and technological applications.

#### 2.2.2 Artificial Active Colloids

Inspired by NAMs, artificial active colloids—often termed micro/nanomotors or synthetic active colloids—achieve self‐propulsion through self‐generated or externally imposed asymmetry. These systems utilize diverse energy sources, including chemical, optical, magnetic, acoustic, and electrical stimuli, which can be categorized as either homogeneous or heterogeneous. Here, “homogeneous” refers to energy sources uniformly distributed around the active agent, while “heterogeneous” describes asymmetric distributions in either magnitude or direction. Notably, the alternating current (AC) electric field is treated as homogeneous stimuli here due to the size mismatch between the colloids and the field's wavelength. Active colloids typically measure between 10^−8^ and 10^−5^ meters, whereas the wavelengths of commonly applied AC fields range from 10^2^ to 10^3^ meters. Consequently, the influence of a simple harmonic AC wave, expressed as $E\left(t\right)=E_{0}\cos\left(\omega t+\phi \right)$, can be approximated by its effective root‐mean‐square value, $E_{rms}=\frac{E_{0}}{\sqrt{2}}$. Similarly, ultrasound stimuli with frequencies in the range of kHz–MHz (frequently employed in active colloid systems) also feature wavelengths of 10^0^–10^4^ m, classifying them as homogeneous stimuli.

**Figure 2:**
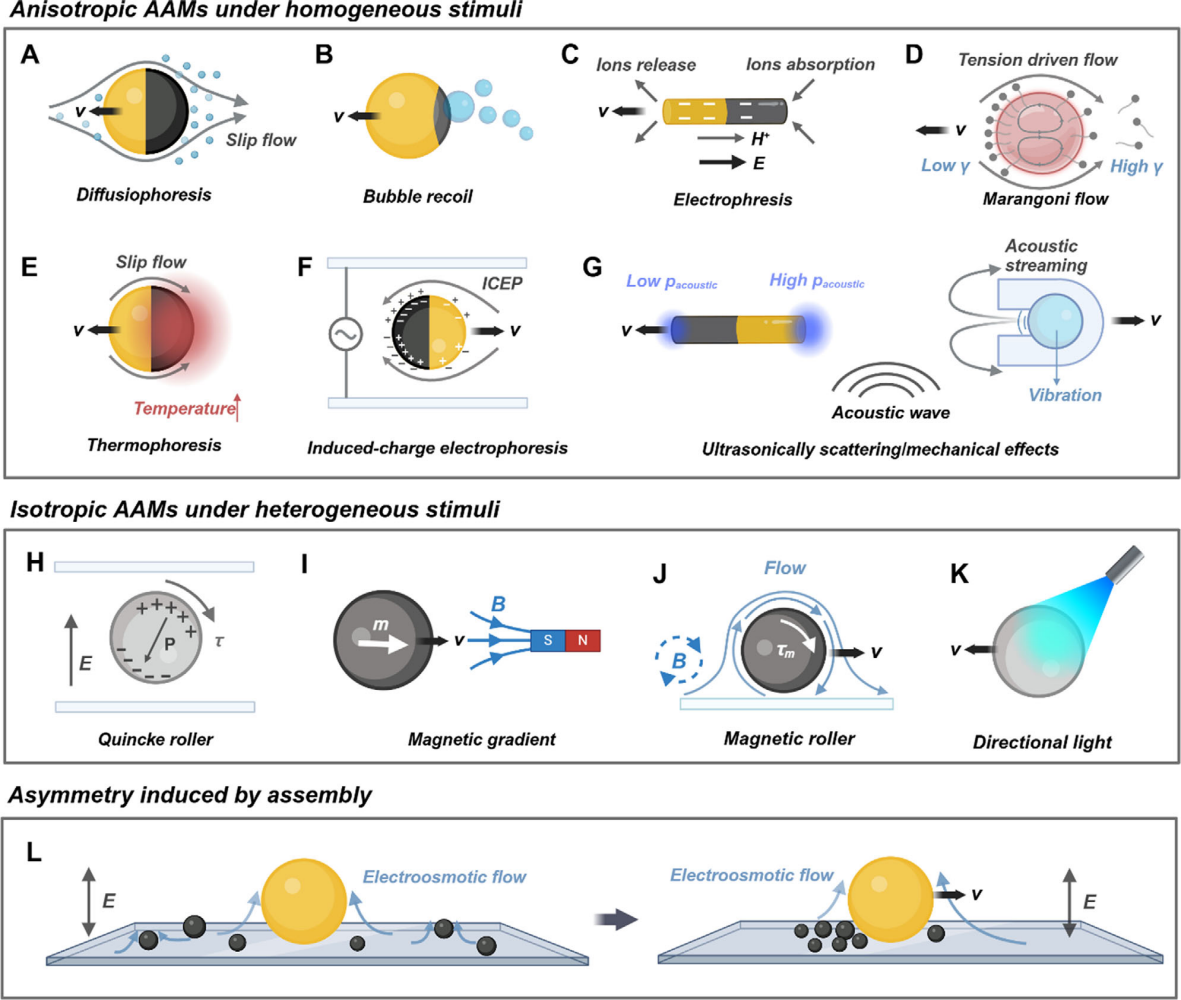
Schematic illustration of propulsion principles for artificial active colloids. A) Diffusiophoresis of a catalytic Janus particle in the presence of chemical fuel. The dark hemisphere indicates the catalytic side. B) Bubble propulsion of a reactive anisotropic particle. The dark part indicates the catalyst. C) Self‐electrophoresis of an anisotropic bimetallic nanorod in the presence of chemical fuel. Dark arrows indicate the electric field. D) Marangoni flow of an active droplet stabilized by surfactant. E) Self‐thermophoresis of a Janus particle. The dark hemisphere indicates the side with the photothermal effect. F) Induced‐charge electrophoresis of a metal‐dielectric Janus particle under an AC electric field. The dark hemisphere indicates the metal side. G) The acoustic pressure, induced by the scattering effect of acoustic waves, on an anisotropic nanorod with different curvature and density on two sides. And the acoustic streaming propulsion of an acoustic microrobot with a bubble trapped in the cavity. H) Quincke rotation of an insulating microsphere under a DC electric field. I) Translational motion of a magnetic agent in the magnetic gradient field generated by a magnet. J) Translational motion of a magnetic roller near a substrate, under a rotating magnetic field. K) Mobility of a semiconductor particle induced by a directional light. L) Symmetry breaking induced by assembly of active colloids under an AC field.

For anisotropic active colloids, the intrinsic structural or compositional asymmetry allows homogeneous stimuli to induce autonomous motion. In catalytic systems, reactions on the active surface of anisotropic colloids generate local concentrations of products (ionic or non‐ionic), forming gradients. These gradients drive slip diffusiophoresis—a flow induced by the chemical potential difference between the colloid's surface and its surroundings—resulting in propulsion toward the side with lower chemical potential, typically the inert side (**Figure** **2**A).^[^ ^43^ ^]^ In systems involving gas molecules such as H_2_ in Mg‐acid reactions or O_2_ in Pt‐H_2_O_2_ systems, propulsion occurs when gas concentrations exceed solubility limits, causing bubble nucleation, growth, and detachment.^[^ ^44^ ^]^ The resulting recoil momentum propels the colloids (Figure 2B).^[^ ^45^, ^46^ ^]^ Additionally, charge asymmetry in ionic gradients applies extra electric forces to the colloids, inducing electrophoresis (Figure 2C).^[^ ^47^ ^]^ In other scenarios, asymmetric physical phenomena such as surface tension gradients, photothermal responses, electric polarization, scattering, and acoustic wave interactions propel colloids through mechanisms like Marangoni flow (Figure 2D),^[^ ^48^ ^]^ thermophoresis (Figure 2E),^[^ ^49^ ^]^ induced‐charge electrophoresis (ICEP, Figure 2F),^[^ ^50^ ^]^ acoustic pressure effects^[^ ^51^, ^52^ ^]^ and acoustic streaming (Figure 2G).^[^ ^53^ ^]^ The first two mechanisms rely on fluid property variations, specifically tension (γ) and density, which induce the slip flow around particles. Except for chemical stimuli, electrophoresis can also be driven by external electric fields in a process known as induced‐charge electrophoresis (ICEP). For example, anisotropic colloids composed of a metal and a SiO_2_ hemisphere display polarization asymmetry in an AC electric field, with the metal side becoming more polarized than the SiO_2_ side. This polarization disparity creates an uneven charge distribution, generating a local electric field and ionic flow that drives movement toward the more polarized metal hemisphere.^[^ ^54^ ^]^ Ultrasonic stimuli enable material‐specific propulsion mechanisms ideal for biomedical applications. For solid materials, such as metals, acoustic pressure (*p*_acoustic_) differences arising from scattering propel colloids toward regions of lower density or curvature.^[^ ^52^ ^]^ Soft materials like bubbles and liposomes, exhibit bubble vibration under ultrasonic waves, resulting in rapid acoustic streaming (10^1^–10^2^ mm s^−1^) that drives active colloids.^[^ ^55^ ^]^

For isotropic agents, mobility relies on heterogeneous stimuli. A notable example is the Quincke roller, which exhibits random motion when activated by a uniform direct current (DC) electric field. In a conducting fluid, insulating spheres accumulate charge due to conductivity differences between the spheres and the medium, leading to electric polarization (**P**). This charge distribution is inherently unstable and subject to minor fluctuations; spontaneous symmetry breaking in the charge distribution results in electrostatic torque, causing the sphere to rotate randomly (Figure 2H). Magnetic agents, another isotropic system, respond to heterogeneous stimuli and are categorized based on their magnetic hysteresis: ferromagnetic materials (high hysteresis) and paramagnetic materials (low hysteresis). These agents are driven by magnetic torque (***τ***_***m***_) and magnetic force (**F**) in response to magnetic fields that may be gradient‐based, oscillating, or rotating. Magnetic force, present only in the presence of a magnetic field gradient (Figure 2I), expressed as:

(2) $$F=\nabla \left(m\cdot B\right)$$

where **m** is the magnetic dipole moment and **B** represents the magnetic field intensity.^[^ ^56^ ^]^ Magnetic torque arises when the magnetization direction of the agent differs from the direction of the external magnetic field (Figure 2J). The rolling direction of magnetic rollers aligns with the plane of the rotating field, while helices propel perpendicular to the plane.^[^ ^57^ ^]^ Notably, the drag asymmetry provided by substrate (or other physical constraint, like acoustic virtual walls),^[^ ^58^ ^]^ is necessary for the propulsion of roller‐type AAMs.^[^ ^59^ ^]^ Additionally, for isotropic agents composed of semiconductor materials like TiO_2_, CdS, or ZnO, limited light penetration results in an asymmetric light intensity around the agent, which induces asymmetric surface physicochemical reactions (Figure 2K).^[^ ^60^ ^]^

Interestingly, dynamic assembly provides another form of induced asymmetry, where interactions between agents lead to organized structures (Figure 2L).^[^ ^61^, ^62^ ^]^ For example, isotropic dielectric agents exposed to an AC electric field generate symmetric electrohydrodynamic flows. While insufficient for individual propulsion, these flows enable hierarchical self‐organization among particles with differing sizes and dielectric properties, resulting in leader‐follower microswarms and emergent collective behaviors.^[^ ^61^ ^]^

This section introduced the primary principles for AAM propulsion through symmetry‐breaking mechanisms. For a deeper exploration of specific propulsion mechanisms and recent advancements, we recommend several comprehensive reviews offering detailed discussions on energy conversion processes and stimuli‐specific responses driving the diverse motion behaviors of artificial active colloids.^[^ ^44^, ^59^, ^63^, ^64^, ^65^, ^66^, ^67^, ^68^ ^]^

## 3 Calculation of Field

In physics, a “field” is a spatial and temporal distribution of a physical quantity, where each point is assigned a specific value. This quantity can be a scalar, vector, spinor, or tensor.^[^ ^69^ ^]^ A vector field, for instance, can be visualized through a surface wind map, where each point on the map has an arrow indicating wind speed and direction, also called as a rank‐1 tensor field. In biological systems, an “endogenous field” refers to a field generated by a living entity, such as through cellular metabolism, diffusion, or convection. Fields are typically described by three key parameters: the physical quantity (*Q*), intensity (*I*), and direction (*D*), with the positive direction defined as pointing toward higher field strength. Two primary methods are commonly used to calculate field intensity. The first (*method 1*) involves measuring positional physical quantities at different locations and calculating the derivative of the quantity to estimate the field strength:

(3) $$I=\frac{dQ}{dx}$$

However, due to technological limitations, especially in chemical fields, it is often difficult to measure values at every spatial point. In such cases, an alternative approach (*method 2*) is to treat the tissue or organ producing the chemical as a “point source” and calculate the field intensity using the diffusion distance (*S*) of the substance:

(4) $$I=\frac{Q}{S}$$

## 4 Endogenous Fields

### 4.1 Chemical Fields

Chemical fields serve as the foundation for nearly all endogenous fields within biological systems, encompassing both ionic and non‐ionic, polar and nonpolar components. The chemical gradients are often accompanied by additional fields, such as electric and pressure fields. The formation of chemical gradients is primarily driven by processes of reaction, diffusion, and convection. Reaction refers to the local production or consumption of molecules, diffusion involves the movement of molecules from regions of high concentration to low concentration, and convection is the bulk movement of molecules within a fluid. Together, these processes establish the spatial distribution of chemical species within biological tissues, generating gradients that play a pivotal role in various physiological and pathological processes. The common chemical endogenous fields are summarized in **Table** **1**.

#### 4.1.1 O_2_

**Figure 3:**
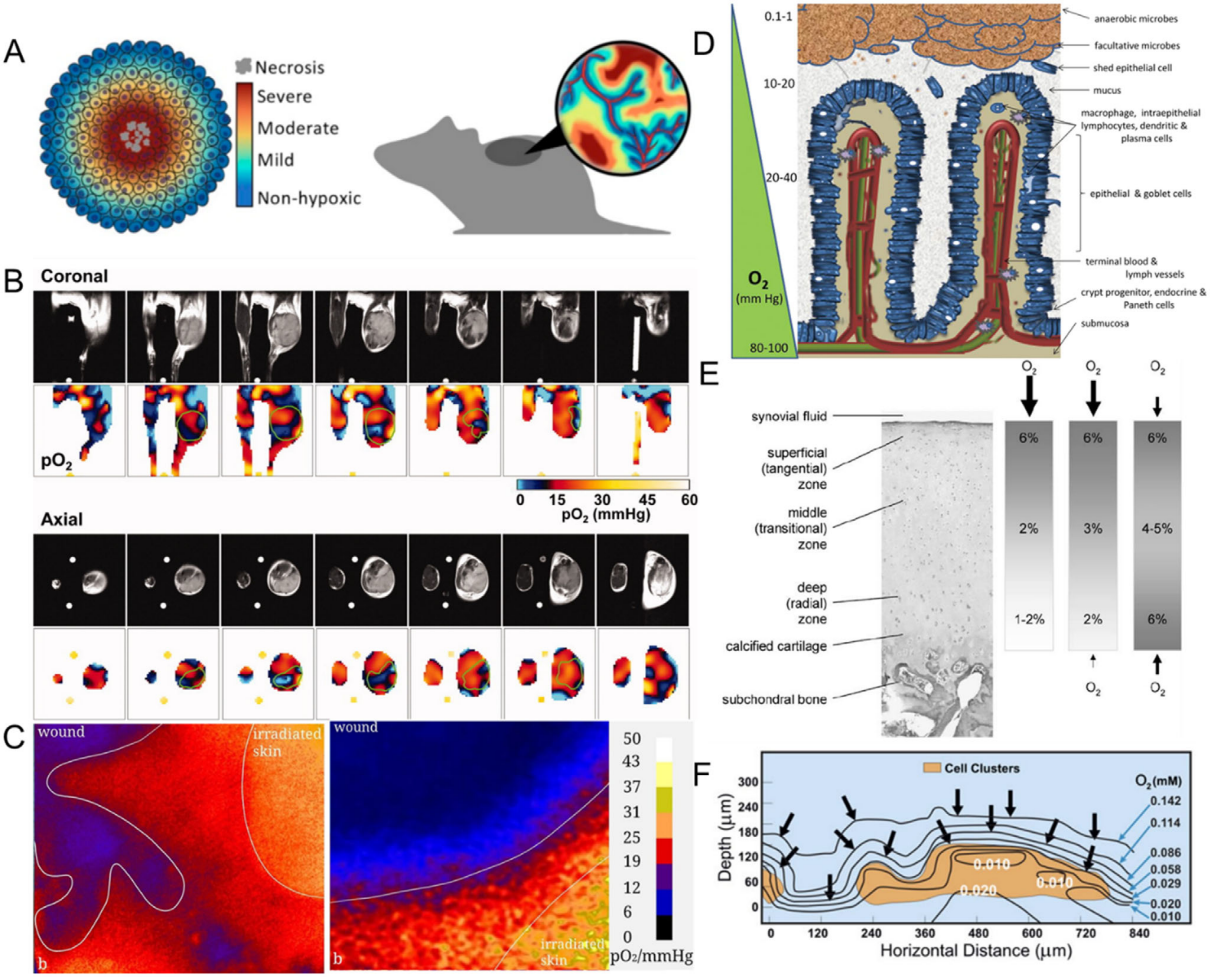
Endogenous O_2_ fields in vivo. A) A spheroid tumor with a gradient in hypoxia levels, transitioning from the periphery (non‐hypoxic tissue) toward the center (necrosis tissue). Reproduced with permission.^[^ ^80^ ^]^ Copyright 2021, Multidisciplinary Digital Publishing Institute. B) Three‐dimensional oxygen mappings in a squamous cell carcinoma (SCC) tumor. Oximetric images of a mouse model bearing an SCC tumor were acquired by electron paramagnetic resonance (EPR) at 10 mT. The coronal and axial sliced images were serially displayed at every 2 mm. Reproduced with permission.^[^ ^81^ ^]^ Copyright 2013, Wiley‐VCH GmbH. C) A clinical case of O_2_ distribution in severe wound healing. Reproduced with permission.^[^ ^72^ ^]^ Copyright 2019, Springer Nature. D) An overview of intestinal villus anatomy in relation to gradients of oxygen that dissipate to near anoxia along the radial axis from the submucosa into the lumen. Reproduced with permission.^[^ ^82^ ^]^ Copyright 2013, Elsevier. E) The structure of articular cartilage and its oxygen supply. Estimated O_2_ levels within the matrix are presented for three scenarios: penetration of O_2_ exclusively from the synovial fluid, O_2_ supply predominantly from synovial fluid (90 %) with a minor contribution from subchondral bone (10 %), supply of O_2_ in equivalent amounts from synovial fluid and subchondral bone. Reproduced with permission.^[^ ^75^ ^]^ Copyright 2007, Springer Nature. F) Dissolved oxygen gradients measured in biofilms. Reproduced with permission.^[^ ^83^ ^]^ Copyright 2024, Montana State University.

Oxygen gradients are critical chemical fields in the body, shaped by vascular delivery and cellular metabolic consumption. These gradients are pervasive in both healthy and diseased tissues, with marked variations based on physiological and pathological states. In solid tumors, rapid cellular proliferation and insufficient angiogenesis lead to hypoxia, a hallmark of malignancy (**Figure** **3**A).^[^ ^70^ ^]^ Tumor oxygen levels (partial pressure of oxygen here, *p*O_2_) decline from the periphery to the core, ranging from 40–60 mmHg in healthy tissue to 0–2.5 mmHg in central tumor regions (Figure 3B).^[^ ^71^ ^]^ Hypoxia promotes angiogenesis, metastasis, and resistance to therapies, including radiotherapy and chemotherapy. Similarly, oxygen gradients also form in wounds, driven by vascular disruption. Acute wounds show gradients of approximately 70 mmHg cm^−1^ directed outward, while chronic wounds exhibit lower gradients of 40.8 mmHg cm^−1^ (Figure 3C).^[^ ^72^ ^]^ These gradients influence cellular responses, such as endothelial adhesion and migration.^[^ ^73^ ^]^

The gastrointestinal tract presents a steep oxygen gradient across the intestinal mucosa and gut lumen (Figure 3D). Partial pressures of oxygen reach 80 mmHg deep in the submucosa, but drop to 0.1–1 mmHg in the large intestinal lumen, where anaerobic microbes thrive and facultative anaerobes act as oxygen scavengers.^[^ ^74^ ^]^ And luminal microbiota is the source of many gases, including methane, H_2_, and hydrogen sulfide, which may form gradients impacting the intestinal epithelium and mucosa. Cartilage, being avascular, exhibits low oxygen tensions due to low consumption rate (7 × 10^−15^ mol cell h^−1^) and limited diffusion through its thick extracellular matrix. Chondrocytes experience oxygen levels of 5 % on average, varying from 6 % at the surface to 2%–3 % in deeper regions. Disease states further exacerbate hypoxia by reducing synovial capillarity or increasing oxygen consumption by inflammatory cells (Figure 3E).^[^ ^75^, ^76^ ^]^

Bacteria alter their local chemical environment through both consumption and the production of a variety of molecules, ultimately shaping the local ecology.^[^ ^77^ ^]^ In biofilms, oxygen gradients result from aerobic respiration near the surface, creating anaerobic zones within a few micrometers (Figure 3F).^[^ ^78^ ^]^ These localized gradients regulate microbial behavior, with oxygen serving as a critical component for electron transport and energy generation. Collectively, oxygen gradients across tissues and systems exemplify their central role in maintaining physiological balance and driving responses to injury, disease, and microbial colonization.^[^ ^79^ ^]^

#### 4.1.2 pH

**Figure 4:**
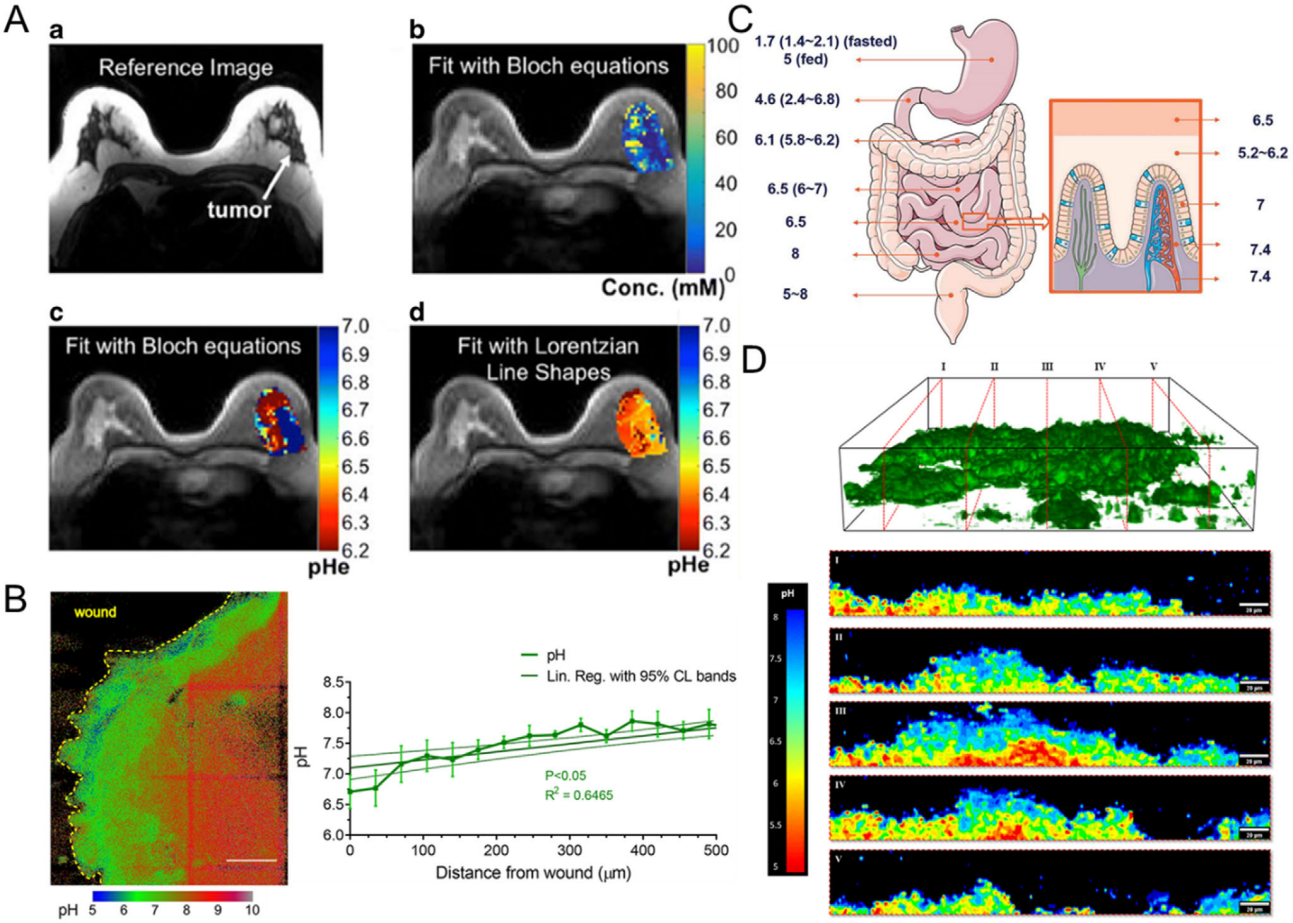
Endogenous pH fields in vivo. A) Parametric maps of the high‐grade invasive ductal carcinoma patient. a) A representative image of the patient with high‐grade invasive ductal carcinoma. b) A parametric map of tumor extracellular pH (pHe). c) A parametric map of tumor concentration determined with Bloch fitting is overlaid on the anatomical image. d) A parametric map of tumor pH determined with Lorentzian fitting is overlaid on the anatomical image. Reproduced with permission.^[^ ^89^ ^]^ Copyright 2017, Springer Nature. B) Analysis of pH in the wound. Maximum intensity projection of a representative pH map stack. The wound margin is highlighted with a yellow dashed line. Scale bars represent 100 µm. Reproduced with permission.^[^ ^85^ ^]^ Copyright 2020, Springer Nature. C) GI physiological pH. Reproduced with permission.^[^ ^86^ ^]^ Copyright 2021, Taylor & Francis Group. D) Representative 3D confocal laser scanning microscopy reconstruction and corresponding ratio metric orthogonal slices showing the pH gradients in the *y*, *z* direction within *P. fluorescens* biofilms. Scale bars represent 20 µm. Reproduced with permission.^[^ ^87^ ^]^ Copyright 2019, American Chemical Society.

pH is a tightly regulated physiological parameter, controlled by endogenous buffers such as bicarbonate, phosphate, histidines on proteins, and sulfates on glycosaminoglycans. It is intricately linked to cellular metabolism and significantly altered in pathological states, including inflammation, ischemia, and cancer.^[^ ^84^ ^]^ The tumor microenvironment (TME) of most solid tumors is characterized by an acidic pH, which stems from the Warburg effect.^[^ ^70^ ^]^ Tumor cells preferentially use glycolysis over oxidative phosphorylation for energy production, leading to lactic acid accumulation in the extracellular environment and a diffusion of hydrogen ions into the stroma. Consequently, intratumoral pH ranges from 6.3 to 6.8 (**Figure** **4**A), promoting proteolytic enzyme activation, extracellular matrix degradation, and tumor metastasis. In normal skin, extracellular pH is neutral to alkaline, but tissue injury induces metabolic shifts to anaerobic processes (less energy‐efficient) under hypoxic conditions, resulting in localized acidosis (pH 6.4–6.7) (Figure 4B).^[^ ^85^ ^]^

The gastrointestinal tract exhibits pronounced pH gradients. The stomach maintains highly acidic conditions with a pH ranging from 1 to 2.5. The mean pH in the proximal small intestine is 6.6 and 7.5 in the terminal ileum (Figure 4C). In the cecum, there is a sharp fall to 6.4, followed by a gradual increase across the colon, reaching a final mean value of 7 in the left colon. While the luminal pH can be significantly affected by the luminal components, the mucus layer that separates the luminal fluid from the microvilli cells maintains a relatively constant pH ranging from 5.2 to 6.2, with an unstirred water layer thickness of 30–100 µm in vivo.^[^ ^86^ ^]^

Biofilms present another dynamic pH environment, as anaerobic fermentation under low oxygen tension produces organic acids,^[^ ^87^ ^]^ such as lactic and acetic acid, creating localized acidic zones. The extracellular polymeric substances (EPS) matrix buffers these fluctuations, maintaining a microenvironment distinct from the surroundings (Figure 4D). For instance, *Escherichia coli* adapts to pH extremes via receptor methylation of Tar and Tsr, demonstrating precise regulatory mechanisms.^[^ ^88^ ^]^

#### 4.1.3 ROS

**Figure 5:**
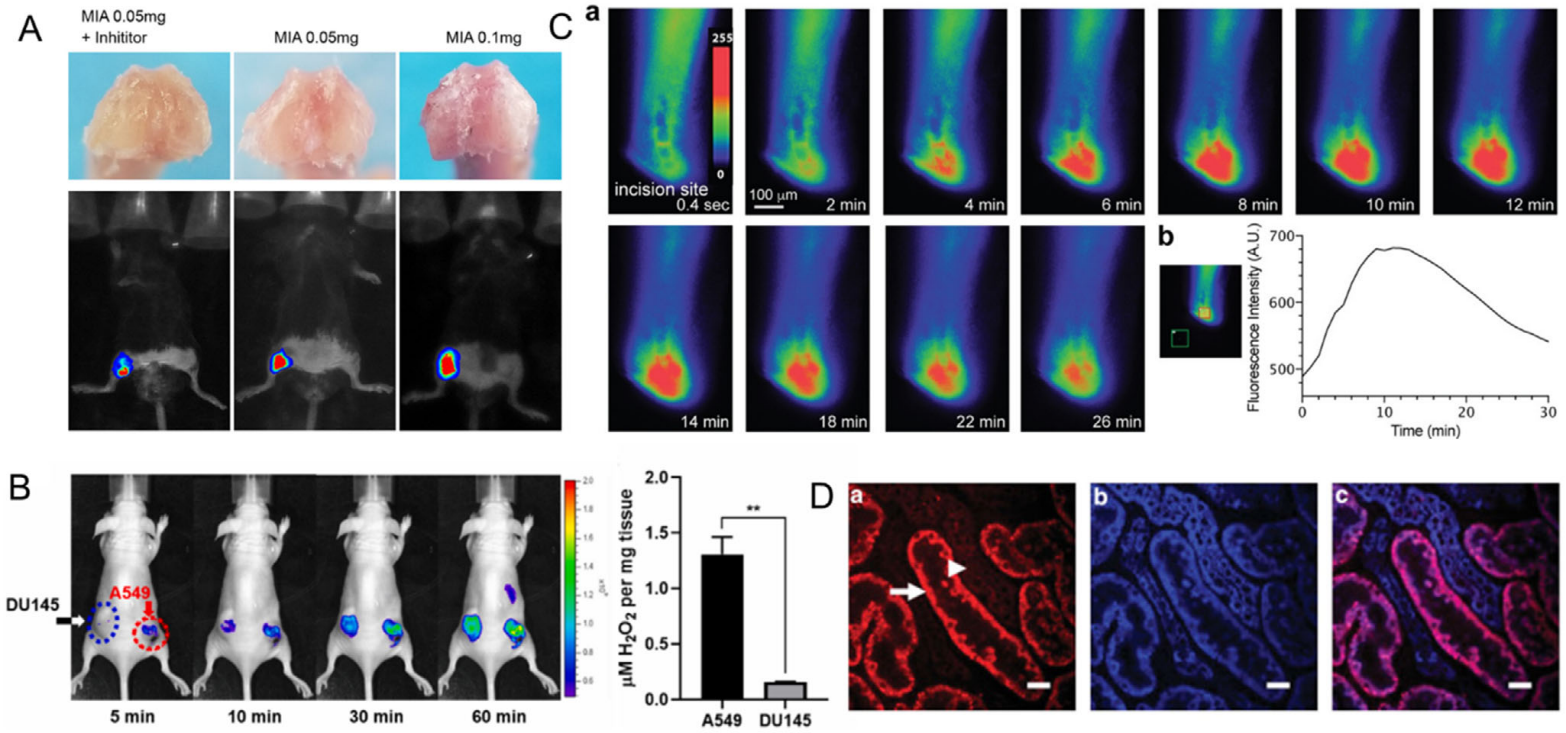
Endogenous ROS fields in vivo. A) Representative photographs showed the macroscopic appearance of the cartilage from the femoral condyles, while in vivo NIR bioimaging indicated the level of ROS via the fluorescent intensity according to the progression of osteoarthritis. Monosodium iodoacetate (MIA) is an inhibitor of glyceraldehyde‐3‐phosphate dehydrogenase activity, including inducing oxidative stress injury and the pathological osteoarthritis symptoms of chondrocytes. Reproduced with permission.^[^ ^91^ ^]^ Copyright 2021, Springer Nature. B) Time‐dependent in vivo fluorescence imaging of ROS in a ROS‐high A549 lung tumor (right flank) and ROS‐low DU145 prostate tumor (left flank), and the relative fluorescence intensity at the region of interest (ROI) (right). And concentrations of H_2_O_2_ present in tumor tissues. Reproduced with permission.^[^ ^97^ ^]^ Copyright 2024, Elsevier. C) Snapshots of the fluorescence imaging of wound‐induced H_2_O_2_ and the fluorescence intensity over time. The *y*‐axis represents the fluorescence intensity in the red square relative to that in the green square. Reproduced with permission.^[^ ^94^ ^]^ Copyright 2020, Wiley‐VCH GmbH.D) ROS in the kidney by the reactive ROS‐sensitive dye dihydroethidium (HEt), the fluorescence signal in proximal tubules (arrow) was higher than that in adjacent distal tubules (arrowhead). Reproduced with permission.^[^ ^98^ ^]^ Copyright 2013, Elsevier.

ROS, including superoxide (O_2_^·−^), hydrogen peroxide (H_2_O_2_), and hydroxyl radicals (·OH), are primarily generated by NADPH oxidases, the mitochondrial respiratory chain, xanthine oxidase, and nitric oxide synthases.^[^ ^90^ ^]^ These systems are tightly regulated to balance ROS production and catabolism, preventing oxidative stress while supporting essential signaling and immune functions. Mitochondria are major contributors, especially under hypoxia, where electron leakage enhances superoxide formation,^[^ ^91^ ^]^ subsequently converted to H_2_O_2_. The regulation of ROS is essential, as excessive ROS levels lead to oxidative stress, which can cause irreversible cellular damage, while insufficient ROS can impair cellular signaling and immune responses.^[^ ^92^ ^]^ Pathological conditions exacerbate ROS imbalance. In osteoarthritis, ROS concentrations in affected joints reach 50–100 µm, accelerating cartilage degradation via protein carbonylation and hyper‐peroxidation (**Figure** **5**A). Additionally, malignant tumor cells exhibit H_2_O_2_ levels up to 100 mm, far exceeding normal cellular concentrations (below 20 nm).^[^ ^93^ ^]^ Within the tumor microenvironment (TME), ROS, produced by cancer and stromal cells, drive immune suppression, angiogenesis, metastasis, and therapeutic resistance, with tissue H_2_O_2_ concentrations ≈1.2 µm mg^−1^ (Figure 5B).

ROS gradients play critical roles in tissue responses. Following injury, H_2_O_2_ is rapidly produced, acting as a chemoattractant to mobilize immune cells within seconds to minutes (Figure 5C).^[^ ^94^ ^]^ Similarly, ROS gradients in the kidney, particularly in proximal tubules with dense mitochondria, contribute to damage localization during acute kidney injury (Figure 5D). In the healthy organs, hydrogen peroxide concentrations in the lungs in situ may be well expected in the micromolar to tens‐micromolar range.^[^ ^95^, ^96^ ^]^ Curiously, information on ROS concentrations at specific sites is scarce, so we recommend using *method 2* (Section 3) to calculate field intensity, and the diffusion distance of ROS is shown in **Table** **2**.

**Table 2:** Effective diffusion distance and lifetime of species.

Species	Diffusion distance	Lifetime
Nitric oxide (NO^•^)	10 m^[^ ^145^ ^]^	≈1 s
Superoxide radical anion (O_2_^•‐^)	320–500 nm^[^ ^146^ ^]^	≈50 ms
Hydroxyl radical (OH•)	4.5 nm^[^ ^145^ ^]^	≈10^−9^ s
Nitrogen dioxide (NO_2_•)	188 nm^[^ ^145^ ^]^	–
Hydrogen peroxide (H_2_O_2_)	1.5 mm^[^ ^145^ ^]^	≈1 ms
O_2_	0.1–0.2 mm (healthy tissue)^[^ ^147^ ^]^ 0.03–0.07 mm (tumor)^[^ ^148^ ^]^	≈420 min

### 4.2 Physical Fields

#### 4.2.1 Electromagnetic Field

Endogenous electric fields (EFs) are present throughout the human body, extensively studied in NAMs and ignored in AAMs. In tissues, organs, and cells, and are inextricably linked to chemical fields due to ionic charge carriers like Na⁺ and Cl^−^ in physiological fluids.^[^ ^149^ ^]^ EFs can be broadly classified into action potentials and extracellular space potentials. Action potentials involve rapid voltage changes across the membrane due to ion channel conductance, crucial for neuronal signaling and muscle contraction. These transient events are highly localized, whereas extracellular space potentials are sustained, spanning hundreds of microns. These fields are typically steady DC gradients of electrical potential that arise in extracellular spaces and are pivotal for processes such as tissue repair, development, and organization.

**Figure 6:**
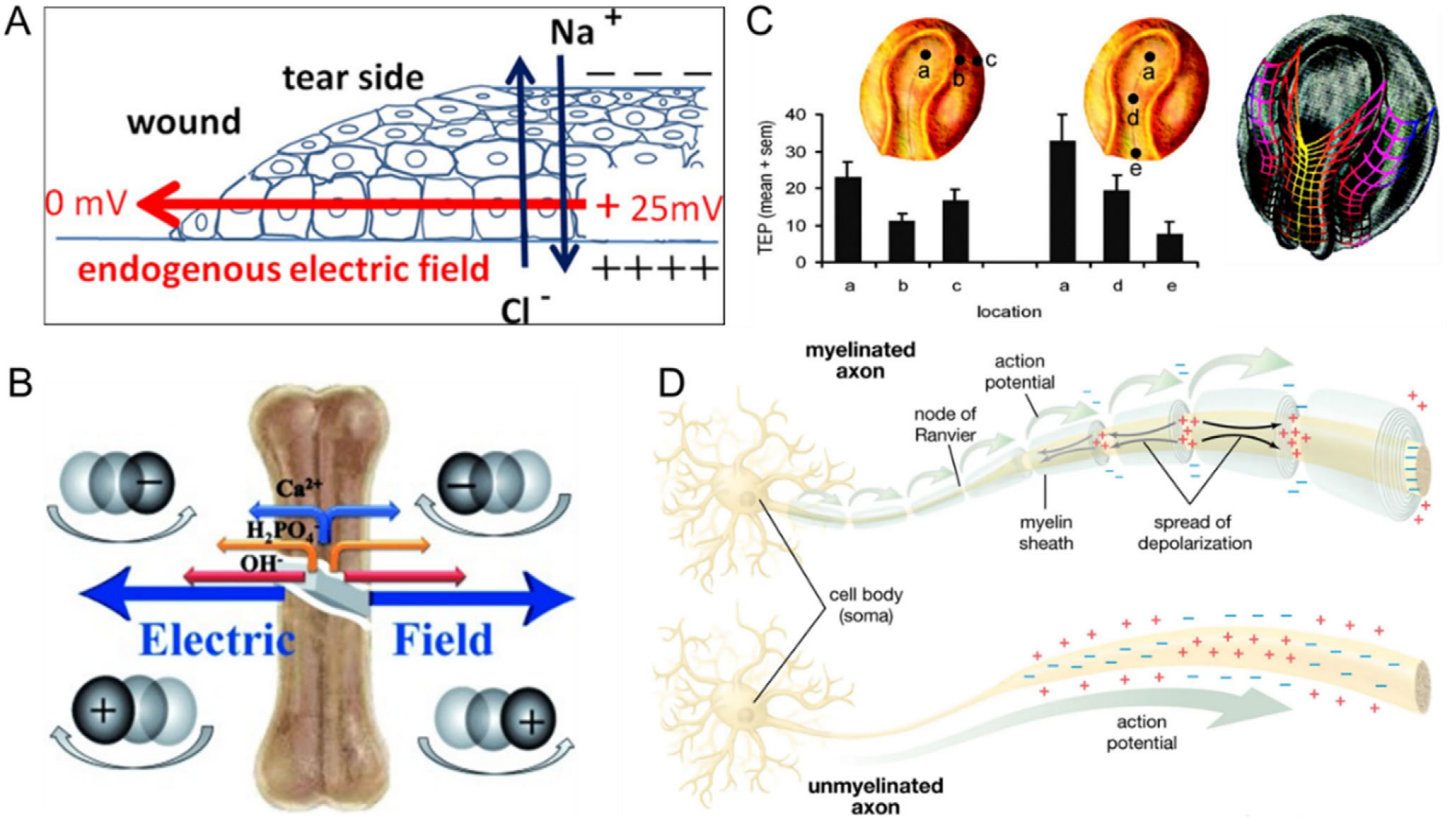
Endogenous electromagnetic fields in vivo. A) EF formation in the wound due to ions and charged particles flowing out from the wound edge. Skin and other epithelial layers establish laterally orientated endogenous electric fields that point to the wound center upon being wounded. Reproduced with permission.^[^ ^166^ ^]^ Copyright 2009, Elsevier. B) Depiction of the electric field in the bone fracture, induced by the ion gradient. The lengths of the arrows adjacent to the ions represent their relative mobilities. Reproduced with permission.^[^ ^167^ ^]^ Copyright 2013, Wiley‐VCH GmbH. C) Spatial differences in the transepithelial potential difference (TEP) generate electric fields within intact embryos. a: at the rostral end of the neural groove; b: at the lateral edge of the neural fold; c: within the lateral epithelium; d: halfway along the neural groove; e: at the caudal end of the neural groove near the blastopore. On the right is an artist's impression of the spatial differences of TEP in an embryo. Colors represent the magnitude of the TEP. Yellow is the highest, and purple is the lowest. Reproduced with permission.^[^ ^166^ ^]^ Copyright 2009, Elsevier. D) Action potential on nerve cells. Reproduced with permission.^[^ ^166^ ^]^ Copyright 2009, Elsevier.

Tissue injury disrupts trans‐epithelial potential difference (TEPD), generating lateral EFs orthogonal to TEPD (**Table** **3**). These wound‐induced EFs, ranging from 40 to 200 mV mm^−1^ (toward wounds) depending on tissue and damage extent, promote directed cell migration essential for repair (**Figure** **6**A).^[^ ^150^ ^]^ For example, in corneal wounds, the formation of such an EF is crucial for re‐epithelialization^[^ ^151^ ^]^ where the potential difference between the wound site and adjacent intact tissue guides epithelial cell migration. The extracellular injury electric field is ≈580 mV mm^−1^,^[^ ^152^ ^]^ and the boundary between the ischemic and normal tissue, through which the current flows, is estimated to be around 8 mm.^[^ ^153^ ^]^ Tumor invasion and deformation often disrupt epithelial integrity, leading to TEPD collapse (50–500 mV mm^−1^) and reversal of EF direction,^[^ ^154^ ^]^ which influences cancer cell behavior. Additionally, differential surface charges between proliferative and non‐proliferative regions further contribute to EF formation.^[^ ^155^ ^]^

In vascular systems, EFs regulate endothelial zeta potentials (100–400 mV),^[^ ^156^ ^]^ maintaining blood flow, vascular permeability, and clotting mechanisms.^[^ ^157^ ^]^ In bone tissue, endogenous electronegative potentials appear immediately after injury (Figure 6B), which could directly control cell proliferation and differentiation.^[^ ^158^ ^]^ Additionally, all embryonic cells during normal developmental migration are surrounded by the embryonic electric field.^[^ ^159^ ^]^ It has been established that the embryonic electric field originates from the ionic flow generated by the pumping of cations, such as Na^+^, into the embryo by the embryonic epithelium (Figure 6C).^[^ ^160^ ^]^ The vector direction of this EF is consistent with the main axis of the embryo, points to the neural groove. The primitive zone of the early chicken embryo exhibits an external leakage current of 100 µA cm^−2^.^[^ ^160^ ^]^

The nervous system, composed of electrically polarized neurons, generates complex EM fields as part of normal brain function. The nerve growth cone was often measured to have a DC current of 10–100 µA cm^−2^, and this resulted in a steady DC electric field of 10–100 mV mm^−1^ according to Ohm's law based on the tissue resistance.^[^ ^161^ ^]^ EFs in neuron, with average intensities around 2 mV mm^−1^,^[^ ^162^ ^]^ is sufficient to shift neuronal membrane potential by 0.1 to 1.3 mV and modulate brain activity when applied at low frequencies (< 2 Hz) (Figure 6D).^[^ ^163^ ^]^ Recent studies have measured the brain's magnetic fields using magnetoencephalography (MEG), detecting magnetic signatures as low as 25–100 nT at the cortical surface.^[^ ^164^ ^]^ These brain‐generated fields are involved in various cognitive functions and neurological disorders.^[^ ^165^ ^]^

#### 4.2.2 Light Field

Ultraweak photon emissions (UPEs) are a phenomenon observed in both unicellular and multicellular organisms (Table 3). Unlike bioluminescence, which stems from specific enzymatic reactions involving luciferin and luciferase, UPEs represent low‐intensity visible light emissions detected in various animal tissues and cells, including organs such as the liver, heart, lungs, nerves, and muscles, as well as biological fluids like blood and exhaled air under normal physiological conditions. These emissions have attracted attention for their potential role in cellular metabolic regulation, although comprehensive quantitative data are limited. For instance, mouse liver emits photons at physiological temperatures, and perfused rat liver exhibits spontaneous emissions ranging from 7 to 12 photons s^−1^ cm^−2^.^[^ ^168^ ^]^ Notably, these emissions are oxygen‐dependent and increase significantly when hydroperoxides are introduced. Cell fractionation studies have pinpointed mitochondria and microsomal fractions as primary sources of UPEs, particularly under conditions of oxygen and hydroperoxide presence.^[^ ^169^ ^]^

UPEs are thought to result from the relaxation of electronically excited species generated during oxidative metabolic processes, including lipid, protein, and nucleic acid metabolism, driven by reactive oxygen species (ROS).^[^ ^170^ ^]^ For example, localized inflammatory responses lead to increased photon production, which subsides as the inflammation resolves.^[^ ^171^ ^]^ Phagocytosis, involving high levels of oxygen radicals, also correlates with enhanced luminescence, with singlet oxygen playing a pivotal role as a long‐lived and biologically active species.

The human brain has also been shown to emit weak light pulses across ultraviolet, visible, and infrared spectra. These emissions are distinct from bioluminescence and are linked to the brain's intrinsic biochemistry, such as ROS production and microtubule dynamics.^[^ ^172^ ^]^ This photon emission, occurring independently of conventional sensory systems like retinal photoreceptors, implies that the brain can emit and potentially detect light in a manner unrelated to vision.

#### 4.2.3 Flow and Pressure Field

Fluid flow within the human body is an essential physiological process, involving the movement of blood, lymph, cerebrospinal fluid (CSF), and other bodily fluids across various systems. These flows, mainly involved in circulatory, respiratory and interstitial systems, facilitate the delivery of nutrients, oxygen, hormones, and immune cells to tissues while ensuring the efficient removal of metabolic waste. Fluid flow is intricately linked to pressure fields, dominated by the applied force (such as muscle contraction) and molecule distribution (chemical potential). Blood pressure, generated by the heart's pumping action, typically ranges from 100 to 140 mmHg in arteries to 30 mmHg in capillaries, driving blood circulation and enabling capillary exchange for nutrient and gas diffusion (Table 3).^[^ ^173^ ^]^ The lymphatic system collects excess interstitial fluid, ensuring fluid balance and supporting immune function.

The delicate balance between fluid flow and pressure fields can be disrupted in pathological conditions such as cancer. In healthy tissues, interstitial fluid pressure (IFP) typically fluctuates between –3 and +3 mmHg, maintaining homeostasis. In contrast, tumors exhibit structural abnormalities and increased vascular permeability, disordered blood flow, a stiffened extracellular matrix, and impaired lymphatic drainage, leading to elevated IFP. This pressure can vary from <1 kPa (7.5 mmHg) in brain tumors to 5 kPa (37 mmHg) in renal cell carcinomas.^[^ ^174^ ^]^ This elevated pressure drives interstitial fluid from the tumor core to the surrounding normal tissues, generating fluid flow that exerts shear stresses on nearby cells. Fluid field and pressure field affect the active matter usually by mechanotransduction mechanisms. In tumors, shear stresses can activate cancer cell motility, induce matrix metalloproteinase (MMP) activity, and promote tumor invasion and metastasis. Furthermore, these fluid forces can influence stromal and immune cell behavior, affecting processes like angiogenesis, lymphangiogenesis, and immune regulation.^[^ ^175^ ^]^

#### 4.2.4 Mechanical Field

**Figure 7:**
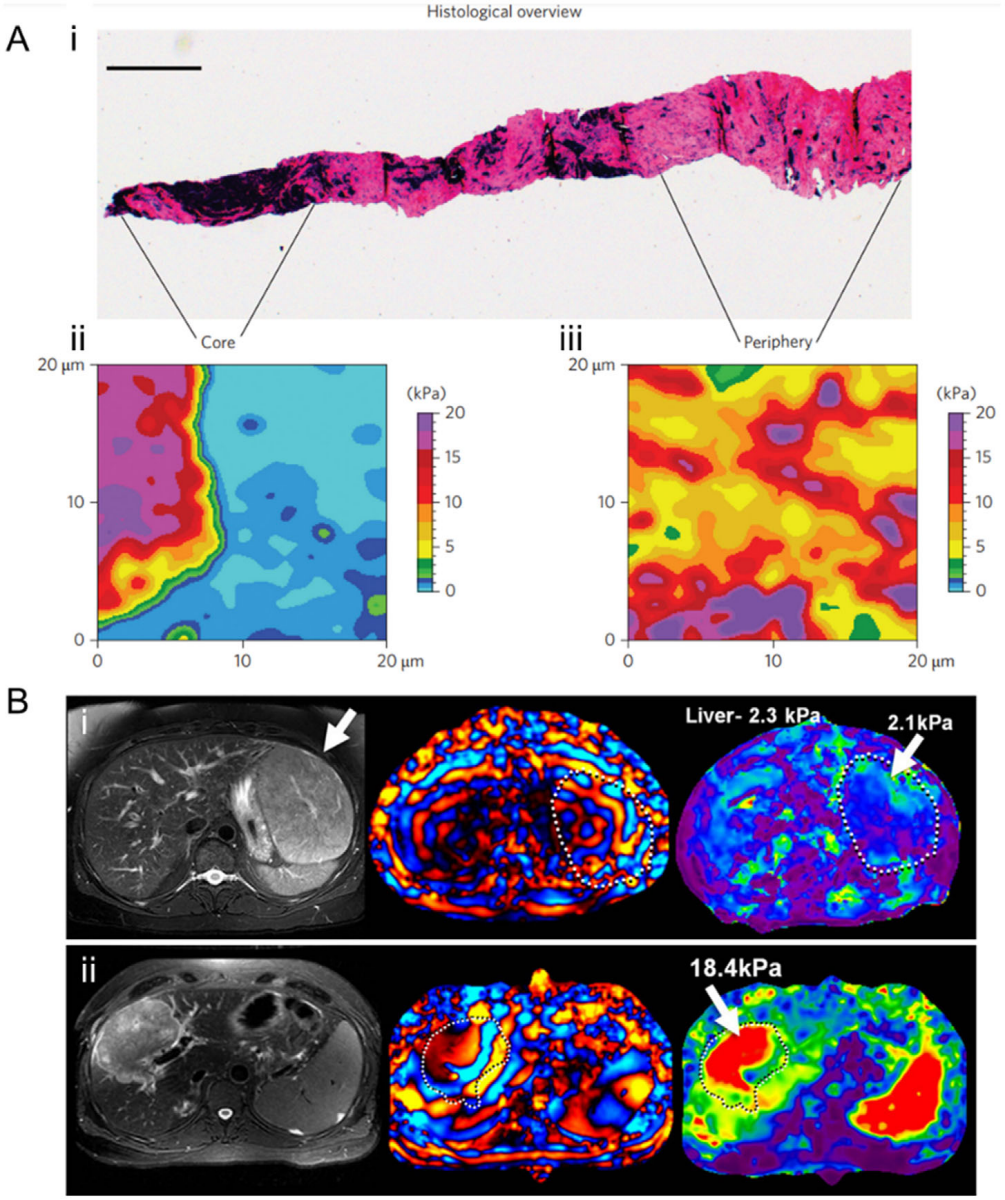
Endogenous mechanical fields in vivo. A) i) Post‐AFM histological overview of the entire cancer biopsy. Scale bar, 500 mm. ii) Representative AFM stiffness map (24 × 24 px) of the core region. iii) A typical stiffness map (24 × 24 px) of the tumour periphery demonstrates stiff features. Reproduced with permission.^[^ ^177^ ^]^ Copyright 2012, Springer Nature. B) Magnetic resonance elastography of i) normal liver and ii) hepatocellular carcinoma‐cholangiocarcinoma. Reproduced with permission.^[^ ^178^ ^]^ Copyright 2013, Elsevier.

A mechanical field refers to the distribution of forces within a biological system that influences the physical state and behavior of cells and tissues (Table 3). Tumor microenvironment (TME) comprises tumor cells and stromal components, including the extracellular matrix (ECM), basement membrane, vasculature, immune cells, and fibroblasts. During tumor progression, all components change their physical structures and functions. With a few exceptions, primary tumors across various cancer types exhibit increased mechanical rigidity compared to their healthy tissue counterparts. This heightened tissue stiffness is primarily attributed to the excessive deposition and enhanced crosslinking of ECM, particularly collagen.^[^ ^176^ ^]^ This mechanical environment is also closely associated with elevated interstitial fluid pressure and hypoxia. In human breast tumor biopsies, the periphery of tumors exhibits a sevenfold increase in stiffness (E = 5.51 ± 1.70 kPa) compared to the tumor core (E = 0.74 ± 0.26 kPa), whereas healthy breast tissue has a stiffness range of 1.13–1.83 kPa (**Figure** **7**A).^[^ ^177^ ^]^ In liver tissue, normal parenchymal stiffness remains below 5 kPa, while malignant tumors present significantly higher stiffness values ranging from 14–18.4 kPa (Figure 7B).^[^ ^178^ ^]^ On substrates of varying stiffness (2.83 kPa vs. 34.88 kPa), cancer cells demonstrate enhanced migration and proliferation, generating higher traction forces (620 Pa vs. 200 Pa).^[^ ^179^ ^]^

Biofilms, defined as complex microbial communities embedded within a 3D extracellular matrix, contrast with the planktonic lifestyle by protecting against mechanical, chemical, and environmental stresses. The matrix ensures mechanical cohesion, contributing to the survival and persistence of embedded microorganisms. The mechanical properties of biofilms, influenced by population density and morphology, show significant differences compared to individual bacterial cells.^[^ ^180^ ^]^ Compared to kPa order of EPS, the Young's modulus of *E coli*. bacteria biofilm is approximately 1 to 10 MPa, which is three orders of magnitude stiffer than the bacteria.^[^ ^181^ ^]^

#### 4.2.5 Topological Field

Topological fields, encompassing defects and structures, arise from geometric confinement and external forces (Table 3). These fields contribute to spatial variations in the alignment and polarity of active matters. In microstructured environments that mimic the extracellular matrix, cells align along topographical features, forming long‐range order interspersed with topological defects.^[^ ^182^, ^183^ ^]^ These defects result from the competition between local cell alignment and external factors such as substrate topography or shear forces.^[^ ^184^ ^]^ In nematic systems, the local orientation of cells becomes disrupted at defect points, creating regions of high curvature or torsion that act as boundaries or attractors, influencing the system's overall dynamics. In polar systems, topological defects like vortices and antivortices, with topological charges of +1 and −1, respectively, direct collective migration by guiding cell flows.^[^ ^185^ ^]^ Distinct growth behaviors are observed between normal and cancerous breast epithelial cells when cultured in 3D matrices compared to 2D environments.^[^ ^186^ ^]^ Surface topography also mediates cell migration through topotaxis,^[^ ^187^ ^]^ in which cells respond to gradients in microenvironmental surface features, impacting processes like cancer invasion and metastasis. However, detailed studies of topological fields in vivo remain limited, and fixed physical parameters for these fields have yet to be established. Second harmonic generation imaging has revealed cross‐linked ECM in tumor tissues and a looser ECM in normal tissues, indicating denser, 3D voids in tumors.^[^ ^188^ ^]^ Additionally, animal cells release traces—such as straight lines, arcs, loops, and branches—onto glass or silicon surfaces during adhesion and migration.^[^ ^189^ ^]^

### 4.3 Complexity of Endogenous Field

The complexity of fields includes heterogeneity, dynamics, and interplay between them. The physiological environment has strong regional differences, and it is dynamic with the selective pressure provided by microenvironmental conditions.^[^ ^190^ ^]^ For example, although the entire tumor exhibits hypoxic characteristics, there are still some vessels that supply O_2_. This spatial association between genetically different cancer cells and blood vessels may be attributed to environmental adaptation, or the ability of cancer cells to modify their environments. In terms of dynamics, upon injury of the skin, the pH value of the wound surface increases owing to the leakage from the microvessels, and approximates a physiologic pH (from 6 to 7.4), accommodating to bacterial infections. Consequently, the pH value becomes more alkaline, commonly between pH values 7.5–8.9, which causes inflammation and leads to prolonged wound healing.^[^ ^191^ ^]^ So far, the characterization of heterogeneous dynamic microenvironments lacks unified and scientific parameters, and it is impossible to give a specific summary here. Here are some reviews to discuss the complexity of the microenvironment.^[^ ^180^, ^192^, ^193^, ^194^, ^195^ ^]^ Further analysis of the endogenous field puts forward high requirements for imaging technologies and analysis methods. We need a comprehensive characterization of multiple microenvironment features and get the mapping, instead of measuring values at certain points.

**Table 3:** Summary of endogenous physical fields.

Classification	Position	Intensity	Direction	Method
Magnetic field	Neocortex	25–100 nT^[^ ^164^, ^196^ ^]^		Magnetoencephalography (MEG)
		10^−16^–10^−18 ^W cm^−2^		
	Forehead	10^−12^–10^−14^ W cm^−2^ (human eye sensitivity)^[^ ^171^ ^]^		
		12.3 ± 0.15 cps^[^ ^197^ ^]^ (320 nm)		
	HT‐29 cell	9.5 ± 0.14 cps^[^ ^197^ ^]^ (360 nm)		
		11.9 ± 0.14 cps^[^ ^197^ ^]^ (420 nm)		
	Transplanted bladder cancer (Female nude mice)	10.0 ± 0.13 cps^[^ ^197^ ^]^ (470 nm)		
	Breast cancer	6.9 ± 0.12 cps^[^ ^197^ ^]^ (530 nm)		
Light field		6.1 ± 0.12 cps^[^ ^197^ ^]^ (570 nm)		Photo multiplier tube (PMT)
	Infiltrating ductal carcinoma	5.7 ± 0.13 cps^[^ ^197^ ^]^ (630 nm)		
		5.1 cps (normal)^[^ ^198^ ^]^		
		8.1 cps (4 mM H_2_O_2_)^[^ ^198^ ^]^		
		27.4 ± 20.7 (× 10^−3^ px^−1^ h^−1^)^[^ ^199^ ^]^		
		10.0 ± 5.4 (× 10^−3^ px^−1^ h^−1^)^[^ ^199^ ^]^		
		121 ± 30 (counts min^−1^ cm^−2^)^[^ ^200^ ^]^		
		3243 ± 214 (counts min^−1^ cm^−2^)^[^ ^200^ ^]^		
Electric field	Brain	2 mV mm^−1[^ ^162^ ^]^ (1 Hz)^[^ ^163^ ^]^	–	Silicon recording electrodes
	Injured tissue	40–200 mV mm^−1[^ ^150^ ^]^ 87 mV mm^−1^ (Human skin)^[^ ^201^ ^]^ 400 mV mm^−1^ (wound on the back of a human hand)^[^ ^202^ ^]^	Toward the center of wound	Microelectrode Microneedle Array^[^ ^201^ ^]^ Capacitative coupling based bioelectric field imager^[^ ^202^ ^]^
	Bone	0.5–12 µA cm^−2^ (Intact bone)^[^ ^203^ ^]^ 86.3–102 µA cm^−2^ (Fracture)^[^ ^203^ ^]^	Outward the fracture^[^ ^167^ ^]^	Vibrating probe
	Blood flow	100–400 mV mm^−1[^ ^156^ ^]^	Toward the center of vessel	Microelectrode
Pressure	Mammary adenocarcinoma R3230AC (rat)	≈10.67 mmHg mm^−1[^ ^204^ ^]^	Outward the center of tumor	Micropipette
	Walker 256 carcinoma (rat)	≈23.5 mmHg mm^−1[^ ^204^ ^]^		
	Breast carcinomas (human)	≈24.17 mmHg mm^−1[^ ^205^ ^]^		
	Lymphomas (human)	≈3.83 mmHg mm^−1[^ ^206^ ^]^		
	Renal cell carcinoma (human)	≈31.67 mmHg mm^−1[^ ^207^ ^]^		
	Cervical carcinomas (human)	≈15.83 mmHg mm^−1[^ ^208^ ^]^		
	Rectal cancer (human)	≈1.5 mmHg mm^−1[^ ^209^ ^]^		
	Skin after thermal injury	1.2–2 mmHg mm^−1[^ ^210^ ^]^	Outward the edema	
Fluid	Normal granulation tissue in the rabbit ear	0.59 ± 0.16 µm s^−1[^ ^211^ ^]^		Photobleaching measurements
	Neoplastic tissue in the rabbit ear	0.55 ± 0.16 µm s^−1[^ ^211^ ^]^		
	Lymph flow (mouse)	53 ± 16 µm s^−1[^ ^212^ ^]^		Doppler optical coherence tomography (DOCT)
	Aorta	≈40 cm s^−1[^ ^213^ ^]^		
	Vena cavae	≈15 cm s^−1[^ ^213^ ^]^		
	Capillary	≈30 µm s^−1[^ ^213^ ^]^		Photobleaching measurements
	Lymphatic vessels	280–1350 µm s^−1[^ ^212^ ^]^		
Topology	Mouse Fibroblast (L929)	Dendritic branches (locomotion)^[^ ^189^, ^214^ ^]^ Point pattern ring (stationary)^[^ ^189^, ^214^ ^]^		Scanning Electron Microscopy (SEM) Interference Reflection Microscopy (IRM)
Mechanics	Fibroadenomas (benign lesions)^[^ ^215^ ^]^	≈3.125 kPa mm^−1[^ ^215^ ^]^	Toward the periphery	Ultrasound
	Invasive cancer	≈6.95 kPa mm^−1[^ ^215^, ^216^, ^217^ ^]^		
	Prostate cancer	≈10.25 kPa mm^−1[^ ^218^ ^]^		
	Breast tumor	0.91–1.46 kPa mm^−1[^ ^177^ ^]^		indentation‐type atomic force microscope (IT‐AFM)
	Liver tumor	2.84–3.65 kPa mm^−1[^ ^178^ ^]^		Magnetic resonance elastography (MRE)

## 5 Interaction Between Active Matter and Fields

In this section, the interactions between active matter and fields are categorized into three sequential stages: sensing, transmitting, and executing. Upon the generation of fields, active matter first detects signals through specialized sensory modules. These signals are then transmitted to downstream effectors, often requiring amplification within NAMs to ensure a robust response. Finally, the transmitted signal is converted into the application of asymmetric forces, driving movement. In NAMs, this process predominantly involves cytoskeletal rearrangements, while in AAMs, hydrodynamic slip‐flow mechanisms play a central role, although other factors may also contribute. Tables 4, 5, 6 summarize the information of fields detected by NAMs and AAMs, the signal transmitting processes of them, alongside their experimentally observed interactions. Notably, most current studies rely on in vitro experiments due to technological limitations in in vivo systems, and the data presented here are derived exclusively from in vitro investigations.

**Table 4:** Interaction between NAMs with fields.

Field	Information	Active matter	Sensing	Transmitting	Executing
O_2_	0.5 % mm^−1^	*C. jejuni* ^[^ ^221^ ^]^	Enzyme‐coupled receptor (Aer1, Aer2, Tlp6, Tlp9)	CheA, CheZ, CheO, FliM and FliY involved signaling pathways	Chemotaxis with speed of 45 µm s^−1^
Glucose	–	*E. coli* ^[^ ^222^ ^]^	Enzyme‐coupled receptor (Trg)	CheA, CheY, FliM and FliY involved signaling pathways	Chemotaxis with speed of 2.6 µm s^−1^
CCL19	10 nm mm^−1^	Lymphoid cell^[^ ^238^ ^]^	CC chemokine receptor (CCR7)	FA‐1‐mediated cell–cell contacts	Individual chemotaxis with migration index of 0.32. Collective chemotaxis with migration index of 0.71
MeAsp	180 µm mm^−1^	*E.coli* ^[^ ^238^ ^]^	Trg	CheA, CheY, FliM and FliY involved signaling pathways	As cell density increases, there is a kinetic phase transition from near zero average velocity at low cell intensities to finite average velocities (25 µm s^−1^)
Light	UV irradiation, 10–100 µmol photons m^−2^	*C. jejuni* ^[^ ^240^ ^]^	Channel‐coupled receptor (Channelrhodopsin)	Ca^2+^ induced signaling pathways	Phototaxis with phototactic index of 0.5–0.75
Magnetism	50 mT	*Proteobacteria* ^[^ ^241^ ^]^	Magnetosomes	–	Magnetotaxis
Electricity	0.25–3 V cm^−1^	Keratocytes^[^ ^24^ ^]^	Channel‐coupled receptor (voltage‐gated ion channels)	PI3K, ROCK‐involved signaling pathways	Galvanotaxis with speed of 0.25–1 µm s^−1^
Mechanics	0.16 kPa mm^−1^	Humanmammary epithelial cells (MCF‐10A)^[^ ^236^ ^]^	–	Cell–cell junctions	Individual cell doesn't exhibit durotaxis. Collective durotaxis with speed of 0.5 µm min^−1^
Topology	–	*C. reinhardtii* ^[^ ^242^ ^]^	Mechanosensory	–	Topotaxis with speed from 8 to 80 µm s^−1^

### 5.1 Natural Active Matter

#### 5.1.1 Sensing

**Figure 8:**
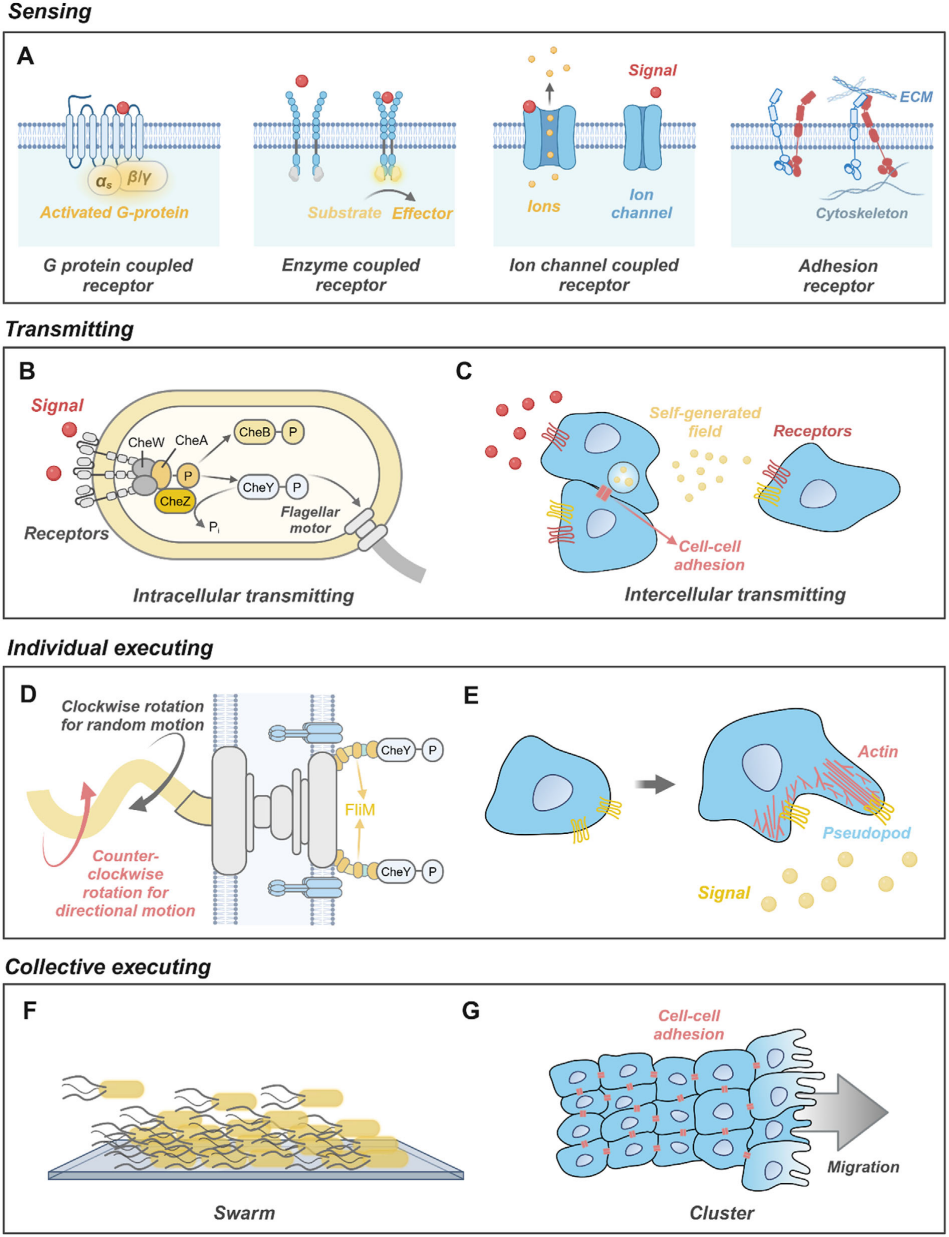
Interactions between NAMs and fields in sensing, transmitting, and executing. A) Four kinds of signaling receptors. B) Signal transmitting inside bacteria during chemotaxis. C) Intercellular transmission by self‐generated signal or cell–cell adhesion. D) Executing of bacteria in the chemotaxis: CheY induces in turn a flagellar rotation switch through the switch complex component FliM, leading to clockwise rotation for random movements or counterclockwise rotation for directional movements. E) Formation of protrusions in cell migration, driven by branched and linearly polymerized actin networks. F) Swarm behavior of bacteria near a substrate. G) Cluster motion of cells based on cell–cell adhesion, and polarization of the front cells.

To sense the diverse signals present in the environment, NAMs rely on various protein receptors embedded in the plasma membrane. Signals cause conformational change of these protein receptors, which switch the ion channels, expose or hide key active sites, or regulate further biochemical reactions. The main types of receptors in NAMs include G protein‐coupled receptors (GPCRs), enzyme‐coupled receptors, ion channel‐coupled receptors, and adhesion receptors (**Figure** **8**A).^[^ ^219^ ^]^

GPCRs bind to signaling molecules and activate associated G proteins, which act as molecular switches to relay signals to intracellular effectors. G proteins stimulate pathways such as the cAMP cascade or phosphoinositide signaling, leading to specific cellular responses. For example, during macrophage chemotaxis, GPCRs like CCR2 and CCR5 detect inflammatory chemokines (e.g., CCL2 and CCL5) released from inflammatory sites.^[^ ^220^ ^]^ Activated G proteins enhance actin polymerization and promote cytoskeletal rearrangement, enabling macrophages to migrate directionally toward higher chemokine concentrations. Enzyme‐coupled receptors modulate intracellular enzymatic activity upon signal binding, thereby influencing specific biochemical pathways. In bacteria, the Aer receptor detects oxygen indirectly by sensing redox changes in its FAD cofactor.^[^ ^221^ ^]^ Activated Aer receptors regulate the kinase activity of CheA, leading to phosphorylation cascades that control bacterial flagellar rotation and orientation. This collaboration with other receptors, like Tsr and Tar, ensures precise navigation in oxygen gradients.^[^ ^222^ ^]^ Ion channel‐coupled receptors regulate ion flow across membranes, inducing rapid changes in membrane potential and intracellular ion concentrations. These alterations trigger a range of downstream effects, such as cytoskeletal remodeling, membrane depolarization, and vesicular transport. For instance, channelrhodopsin (ChR) responds to light by facilitating Ca^2+^ influx,^[^ ^223^ ^]^ which activates actin dynamics and reshapes cellular structures.^[^ ^224^, ^225^ ^]^ Mechanically gated channels, such as PIEZO1/2, respond to mechanical forces by modulating ion flux, which can induce changes in cell morphology or motility.^[^ ^226^ ^]^ Adhesion receptors mediate cell–cell and cell‐ECM interactions, providing spatial information essential for tissue organization. Upon sensing the ECM or neighboring cells, these receptors initiate cytoskeletal remodeling and intracellular signaling pathways that regulate cell behavior. For instance, integrins bind ECM components like collagen and fibronectin, triggering focal adhesion assembly and activating Rho GTPases. This process drives cell polarization and directional migration, as seen in fibroblast movement during wound healing.^[^ ^227^ ^]^

Collectively, these receptors work in concert to sense extracellular signals and coordinate intracellular responses. This coordination often involves crosstalk between signaling pathways triggered by different receptors. Under a DC electric field, negatively charged membrane components redistribute, activating multiple signaling pathways and collaboratively guiding cells toward the anode.^[^ ^24^ ^]^ Similarly, in *Chlamydomonas reinhardtii*, phototaxis is governed by light signals and ROS, where ROS promotes positive phototaxis, while ROS quenchers reverse the response.^[^ ^228^ ^]^

#### 5.1.2 Transmitting

From sensing modules to the machinery that executes the actual movement, there is a transmitting process to benefit efficiency and accuracy. Transmitting here usually involves transformation and amplification of the signal, rather than the simple propagation of the initial signal. Signal signal‐transmitting process in NAMs, encompassing both intracellular and intercellular levels, is primarily governed by diffusion‐convection processes coupled with reaction dynamics:

(5) $$\frac{\partial c_{i}}{\partial t}+\nabla \cdot \left(-D_{i}\nabla c_{i}+c_{i}u\right)=R_{i}$$

where *c_i_, D_i_, R_i_* are the concentration, diffusion, and reaction rate of species *i*, respectively, while **u** denotes fluid flow.

The process of intracellular signal transmitting is typically mediated by a series of cascade reactions involving second messengers such as cAMP, cGMP, IP3 and Ca^2+^ etc. These complex and intricate intracellular signaling pathways can accurately and rapidly convey perceived signals to specific cellular structures, activating their movement. For instance, under microaerophilic conditions, the activated CheA of *Campylobacter jejuni* rapidly transfers a phosphate group to CheY, leading to the phosphorylation of CheY (Table 5). The phosphorylated CheY interacts with the flagellar motor proteins like FliM and FliY, thereby transducing the signal sensed by CheA to the locomotive organelles (Figure 8B).^[^ ^221^ ^]^ A similar mechanism is observed in the phototaxis of algae, where light, as a signaling factor, triggers light‐gated ion channels, allowing Ca^2+^ influx into the algae's eyespot region. The changes in Ca^2+^ concentration in the eyespot region induce a series of electrical responses, then generate photocurrents that are transmitted to voltage‐dependent calcium channels (VDCC) located on the flagella, ultimately altering the flagellar beat and causing the cell to reorient.^[^ ^229^ ^]^

The intercellular signal transmission relies on self‐generated gradients, signaling factors produced by the cells, rather than by the environment. Upon stimulation, cells can produce signaling molecules that act on target cells through blood circulation, diffusion, direct contact, and intercellular junctions (Figure 8C).^[^ ^230^ ^]^ For example, endocrine cells monitor the concentration of specific substances in the blood to regulate the secretion of hormones (a long‐distance signaling molecules). In contrast, local signals are more frequently transmitted via intercellular spaces or junctions. An illustrative example is neurons, which utilize neurotransmitters to convey messages. These intercellular communications facilitate the collective migration behavior. During the collective bacterial chemotaxis, the individual bacteria consume attractants, thereby creating gradients that provide direction for nearby bacteria and coordinate their movement speeds.^[^ ^231^ ^]^ In biological organisms, collective cell migration frequently gives rise to the formation of a polarized supracellular unit, comprising a head and a rear.^[^ ^232^ ^]^ The cells at the front of the supracellular unit become the leading group and form stable lamellipodia or filopodia toward the substrate, and the rear only forms small, transient cryptic lamellipodia. Alternatively, the leading cells may secrete matrix metalloproteinases that remodel the surrounding extracellular matrix (ECM) to pave the way for collective migration.

#### 5.1.3 Executing

Fields trigger diverse cellular behaviors, including proliferation, differentiation, apoptosis, *etc*. Instead of summarizing all the complicated cellular behaviors, this review will only focus on their tactic movement, like chemotaxis, durotaxis, galvanotaxis, haptotaxis, magnetotaxis, phonotaxis, rheotaxis, topotaxis, *etc*. Directed migration cues do not activate special mechanisms of active matter translocation, as discussed in Section 2.1.2, but bias the migration and the polarity machinery that operates during normal, random migration. Here, we explain the main executing mechanisms of NAMs under fields.

In the cases of NAMs that are driven by the asymmetric flow fields, their tactic movement often depends on the coupling of the perception of signaling factors and the transition of movement modes (Figure 8D). Taking *E. coli* as an example,^[^ ^222^ ^]^ when its surface sensory proteins are not bound to ligands, it triggers autophosphorylation of CheA and conveys the signal to the downstream protein CheY. Phosphorylated CheY interacts with the flagellar motor proteins, causing the flagella to rotate clockwise and the bacteria to tumble. However, when its surface sensory proteins are bound to signaling ligands, the enzymatic activity of CheA is inhibited, reducing the phosphorylation level of CheY and decreasing the interaction with the flagella. This change causes the flagella to rotate counterclockwise instead of clockwise, allowing the bacteria to swim smoothly. In this way, within a gradient field of ligands, *E. coli* can sense changes in concentration and switch between these two movement modes, thus achieving taxis toward a favorable direction.

For NAMs driven by asymmetrical stress fields, both mesenchymal and amoeboid movement, they share a common characteristic feature: the formation of protrusive structures on their surface. These protrusive structures are formed by actin filament arrays and typically emerge at the tips of the cells where they sense ligands, aligning with the direction of the environmental signal fields (Figure 8E). The mechanism underlying the tactic migration of these cells is primarily dependent on the selective stabilization of protrusions situated in regions of high or low signal concentrations after the splitting of protrusive structures, as observed in the chemotaxis of *Bacillus subtilis*.^[^ ^232^ ^]^ On the other hand, cells that move based on the mesenchymal mechanism form integrin‐based adhesion structures with the ECM. The specific asymmetric fields exert a corresponding influence on the adhesion structures, resulting in their strengthening or weakening along the direction of the field.^[^ ^232^ ^]^ This provides direction for their tactical movement. It should be noted that the migration mechanisms of cells can convert into each other.^[^ ^233^ ^]^ The concentration of Rac GTPase and Rho in the cell determines the different migration mechanisms of cells to adapt to different environments.

Besides the individual behaviors, fields also induce some collective intelligence, like swarms of bacteria and the cluster motion of cells. Swarming motility generally requires a crowded population density, an energy‐rich environment, and a solid boundary.^[^ ^234^ ^]^ In a dilute liquid environment in which cell–cell interaction is negligible, flagellated bacteria, such as *E. coli*, perform chemotaxis by biased random walks alternating between run‐and‐tumble. And in the bacteria swarm, chemotaxis is enhanced because of cell crowding and is correlated with an increase in the degree of cell body alignment (Figure 8F).^[^ ^235^ ^]^ Some research has shown that durotaxis only exists in multicellular clusters, rather than isolated constituent cells.^[^ ^236^ ^]^ This emergent mode of directed collective cell migration applies to a variety of epithelial cell types, requires the action of myosin motors, and originated from the supracellular transmission of contractile physical forces.^[^ ^237^ ^]^ The organization of cells in sheets and clusters has also been shown to enable collective sensing of both chemical gradients, migrating in leader‐follower mode (Figure 8G).^[^ ^238^ ^]^ When cells are prevented from transmitting forces by disrupting cell–cell junctions or by inhibiting myosin motors, collective gradient sensing is often impaired. Another striking emergent phenomenon in cell monolayers is their ability to propagate mechanical waves. In response to sudden unconfinement, the first row of cells at the monolayer edge spreads and migrates toward the freely available substrate, whereas the cells behind them remain static.^[^ ^239^ ^]^ With time, every cell row becomes progressively engaged in collective motion following a wave of deformation and force generation (**Table** **4**).

### 5.2 Artificial Active Matter

#### 5.2.1 Sensing

**Figure 9:**
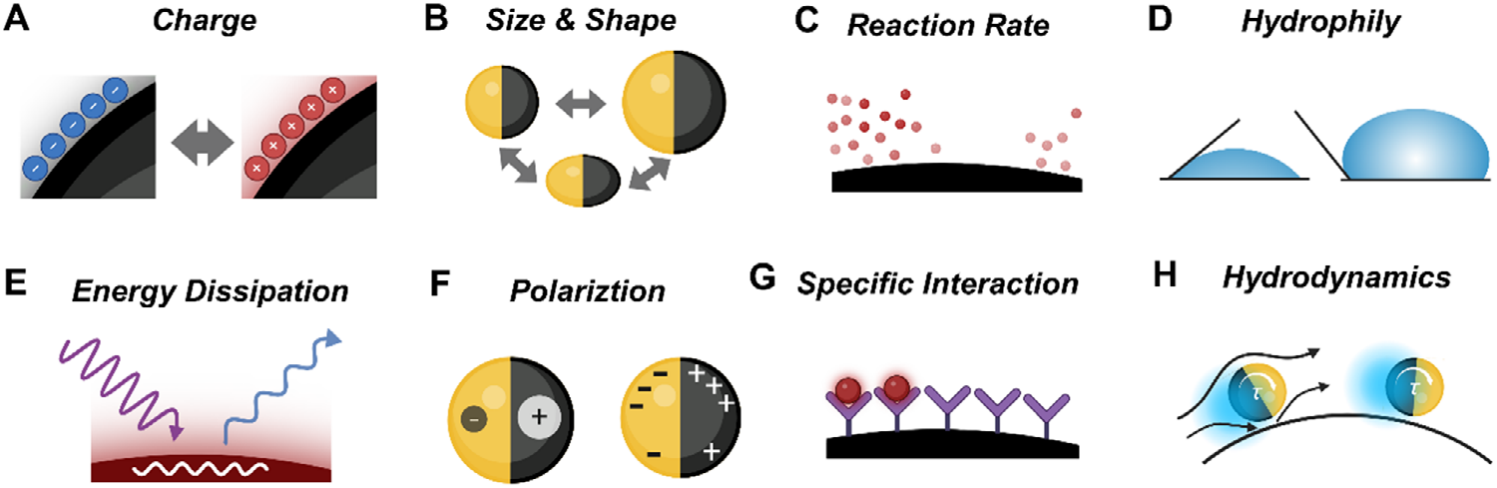
Main principles of AAMs sensing environmental parameters.

The sensory capabilities of AAMs are rooted in the interactions of responsive materials with environmental parameters. These interactions enable various functionalities, including: 1) changes in charge (**Figure** **9**A) size and shape (Figure 9B), 2) modulation in reaction rates (Figure 9C), 3) transitions in hydrophilic properties (Figure 9D), 4) energy dissipation (e.g., heat generation, Bjerknes forces and acoustic streaming, Figure 9E), 5) polarization and magnetization (Figure 9F), 6) specific interaction, like ligand‐receptor interaction (Figure 9G), and 7) hydrodynamic adaptations (Figure 9H). The sensing of AAMs will be introduced through the categories of responsive materials, including polymer, catalyst, biomaterial, and electromagnetic material.

**Figure 10:**
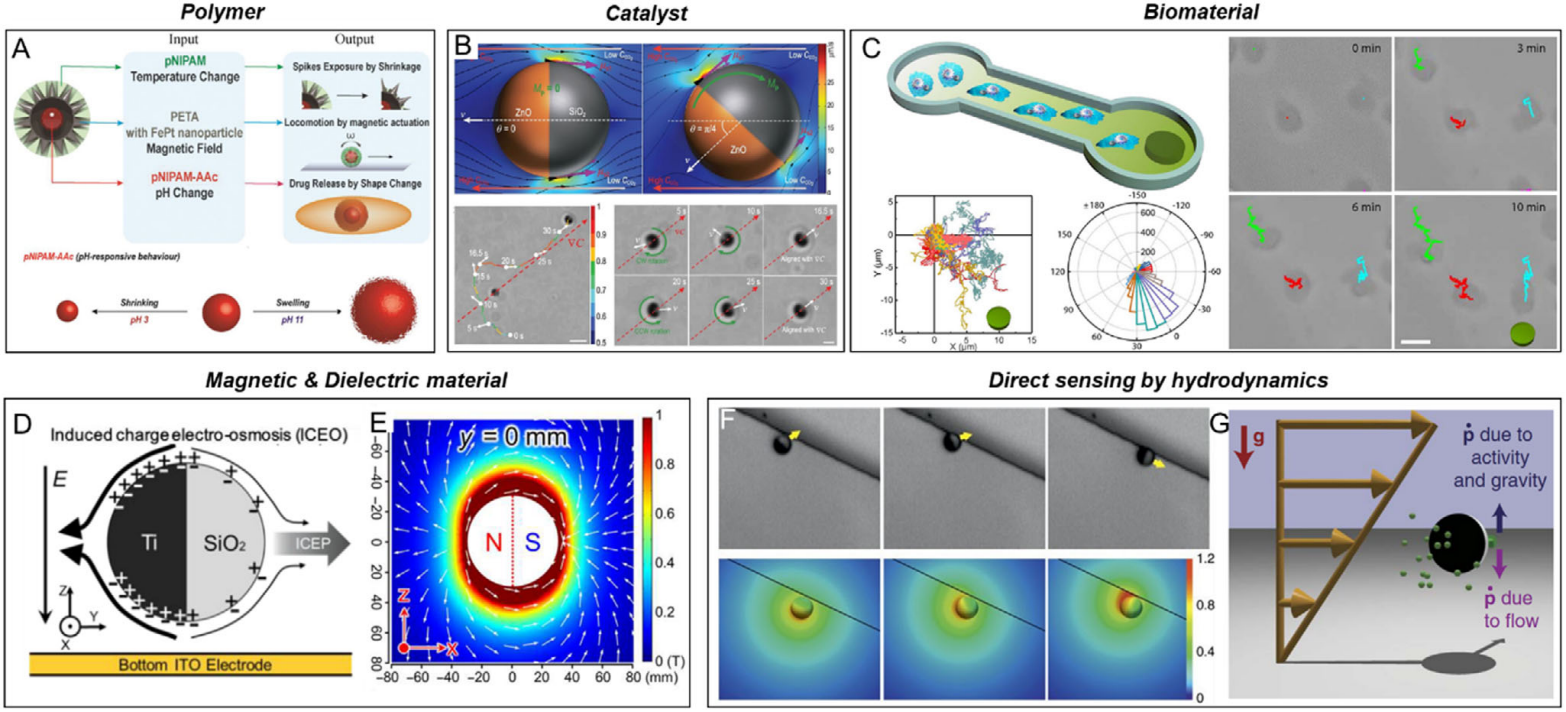
Sensing of AAMs based on responsive materials. A) A multifunctional pollen grain‐inspired hydrogel (MPH) robot is composed of a temperature‐actuated crust shell for controllable attachment, a pH‐responsive sphere structure for cargo releasing, and a magnetic actuated spike structure with pollen grain‐inspired spikes. Reproduced with permission.^[^ ^246^ ^]^ Copyright 2023, Wiley‐VCH GmbH. B) Flow fields around a ZnO/SiO_2_ Janus (black streamlines with black arrows) when its orientation is θ = 0 and θ = π/4, and the trajectory of a slightly etched ZnO/SiO_2_ Janus approaching the CO_2_ source. Reproduced with permission.^[^ ^265^ ^]^ Copyright 2021, Springer Nature.C) Chemotactic motion of neutrophil‐based microrobots along the gradient of chemokine. Reproduced with permission.^[^ ^278^ ^]^ Copyright 2021, American Association for the Science. The D) polarization and E) magnetization in the AC electrical and magnetic field, respectively. Reproduced with permission.^[^ ^19^, ^67^ ^]^ Copyright 2023, American Association for the Science.Copyright 2020, Royal Society of Chemistry. F) A Pt‐SiO_2_ Janus senses and approaches the step by chemical activity and associated hydrodynamic interactions. Reproduced with permission.^[^ ^299^ ^]^ Copyright 2016, Wiley‐VCH GmbH. G) The self‐generated flow of Pt‐SiO_2_ Janus couples with the applied flow and align their propulsion axes to be nearly perpendicular to both the direction of flow and the normal vector of a nearby bounding surface. Reproduced with permission.^[^ ^307^ ^]^ Copyright 2018, SAGE Publishing.

Responsive polymers are particularly notable for their reversible modulation of physicochemical properties, such as conformation, charge, and hydrophilicity, in response to external stimuli. For example, temperature‐sensitive polymers like poly(N‐isopropyl acrylamide) (PNIPAM) undergo phase transitions based on environmental temperature. Below the lower critical solution temperature (LCST), PNIPAM exhibits the hydrophilic state, but above this threshold, it collapses into a hydrophobic state. This temperature sensitivity, tunable through copolymerization with acrylamide (AAm) and acrylic acid (AA), allows PNIPAM‐based AAMs to regulate reaction rates,^[^ ^243^, ^244^ ^]^ mechanisms^[^ ^245^ ^]^ and microrobot shape,^[^ ^246^ ^]^ making them highly effective in sensing thermal fields (**Figure** **10**A). Similarly, pH‐responsive polymers, such as poly(2‐diisopropylamino)ethyl methacrylate (PDPA)^[^ ^246^ ^]^ and poly[2‐(methacryloyloxy)ethyltrimethylammonium chloride] (PMETAC)^[^ ^247^ ^]^ adjust their hydrophilicity in response to pH changes. Notably, some endogenous stimuli for polymers— such as reactive oxygen species (ROS), ions, and glucose—are underexplored in the design of AAMs. Comprehensive reviews on responsive polymers provide additional insights.^[^ ^248^, ^249^, ^250^, ^251^, ^252^ ^]^

Catalytic reactions offer another avenue for sensing environmental fields. In a normal chemical reaction:

(6) $$\alpha A+\beta B\overset{catalyst}{\to }\gamma D+\delta E$$

the reaction rate *r* = *k* *c* ^α^(*A*) *c* ^β^(*B*), where *k* is the rate constant, is highly dependent on environmental parameters, such as substrate concentration like temperature, ions, pH, and redox conditions. This sensitivity not only influences reaction dynamics but also impacts hydrodynamic flows and the distribution of species surrounding active particles, thereby altering their behavior. A widely accepted view is that the AAMs powered by catalysts tend to accumulate in regions with favorable substrate binding, minimizing chemical potential.^[^ ^253^ ^]^ Notable catalytic systems include those involving H_2_O_2_ (e.g., Pt,^[^ ^254^ ^]^ Cu,^[^ ^255^ ^]^ catalase,^[^ ^256^, ^257^ ^]^ nanozyme),^[^ ^258^ ^]^ urea (urease),^[^ ^259^, ^260^ ^]^ ROS (L‐arginine),^[^ ^261^ ^]^ glucose (glucose oxidase),^[^ ^262^ ^]^ glutathione (glutamyltransferase),^[^ ^263^ ^]^ and ATP (ATPase).^[^ ^264^ ^]^ Mou et al. proposed a reorientation mechanism driven by different reaction rates on different locations of SiO_2_‐ZnO Janus microparticles (Figure 10B).^[^ ^265^ ^]^ However, this theory fails to explain reorientation when the angle between the micromotor's orientation and the field exceeds 90°. Additionally, the ability of sensing relying on chemical activity to detect nanoscale differences remains a matter of debate, and thermal perturbations affect it strongly. Fischer and colleagues emphasized the importance of differential phoretic mobility between the catalyst and inert side of Janus particles,^[^ ^266^ ^]^ while He and co‐authors demonstrated that geometric asymmetry‐induced torque is crucial for sensing.^[^ ^264^, ^267^ ^]^ At the nanoscale, Sen's group employed enzyme‐coated liposomes to demonstrate how spatial energy gradients, generated by reaction products, guide the migration of active particles.^[^ ^268^ ^]^ The field responsiveness of enzyme‐based active matter is often explained through mechanisms such as differential diffusivity between bound and free forms^[^ ^269^, ^270^ ^]^ or fluctuation‐induced hydrodynamic effects.^[^ ^271^, ^272^ ^]^

Numerous NAMs, including microalgae,^[^ ^273^, ^274^ ^]^ bacteria,^[^ ^275^, ^276^, ^277^ ^]^ neutrophils,^[^ ^278^ ^]^ macrophages,^[^ ^279^, ^280^, ^281^ ^]^ sperm,^[^ ^282^, ^283^ ^]^ stem cells,^[^ ^284^, ^285^ ^]^ T cells,^[^ ^286^ ^]^ and platelets,^[^ ^287^ ^]^ demonstrate organ‐specific affinity and tumor‐targeting capabilities. These properties are being exploited to engineer cells capable of field sensing and therapeutic delivery (Figure 10C).^[^ ^288^ ^]^ Furthermore, bio‐membrane‐coated AAMs—derived from macrophage,^[^ ^289^, ^290^, ^291^ ^]^ bacterial,^[^ ^292^ ^]^ or platelet membranes^[^ ^293^, ^294^ ^]^—exhibit cell‐like field responsiveness. While the specific mechanisms underlying this behavior remain unclear, it is hypothesized that ligand‐receptor interactions and surface energy variations also play a significant role, as the bio‐membranes mimic the sensing capabilities of natural active matter without contributing to propulsion.

Electromagnetic field interactions also play a significant role in AAM behavior. Under an external electric field, electrons redistribute within a dielectric material, induce polarization, resulting in the formation or reorientation of an electric dipole.^[^ ^67^ ^]^ This dipole induces a non‐uniform surface charge, which attracts oppositely charged ions from the surrounding medium, creating an electroosmotic flow on the particle's surface. In the case of a Janus microsphere with a conductive metal cap (e.g., SiO_2_‐Ti or PS‐Au), this flow, known as induced charge electro‐osmosis (ICEO), is substantially stronger on the metal side due to its higher degree of polarization, leading to movement with the dielectric end forward (Figure 10D). Similarly, magnetically responsive materials reorient their magnetic domains to align with an applied magnetic field,^[^ ^295^, ^296^ ^]^ a phenomenon resulting from electron spin and orbital motion at the microscale (Figure 10E). Leveraging material includes pure metals (e.g., Fe, Co, Ni), alloys (e.g., FeCo, Alnico, Permalloy), ferrites (e.g., Mn_0.6_Zn_0.4_Fe_2_O_4_, CoFe_2_O_4_), and iron oxides (e.g., Fe_3_O_4_, γ‐Fe_2_O_3_), due to their high saturation magnetization, chemical/colloidal stability, and biocompatibility. A recent example is the FePt(L10) microrollers developed by Sitti's group, which demonstrate both high magnetic performance and biocompatibility.^[^ ^297^ ^]^

Besides modifying material properties directly, AAMs adapt to their environment through hydrodynamics.^[^ ^298^ ^]^ Simmchen et al. demonstrated that SiO_2_‐Pt Janus particles detect submicrometer topographic features through both their chemical activity and hydrodynamic interactions, allowing them to navigate along boundaries (Figure 10F).^[^ ^299^, ^300^ ^]^ Furthermore, Katuri and Palacios et al. introduced micropatterned ratchets with complex topographical features, including curvature and chirality, showing that these features can direct the macroscopic flow of AAMs.^[^ ^301^, ^302^ ^]^ Additionally, fields further exert forces on AAMs, including pressure, magnetic, and electrostatic forces via the gradient in magnitude. For particles with mass asymmetry, imposed flows are detected through the interplay between particle polarity and alignment via viscous torque, resulting in positive or negative rheotaxis.^[^ ^303^, ^304^, ^305^, ^306^ ^]^ Furthermore, Jaideep et al. explored how wall interactions, combined with torque from shear flow and active hydrodynamics, reorient active matter perpendicularly to the flow direction (Figure 10G).^[^ ^307^ ^]^

#### 5.2.2 Transmitting

In AAMs, signal sensing induces physicochemical changes on the surface, which can further propagate to other AAMs through either short‐range or long‐range interactions. Short‐range interactions, typically less than 10 nm, include covalent bonding and dipole–dipole interactions. Long‐range interactions rely on the self‐generated physical or chemical fields. Both types of self‐generated fields predominantly induce hydrodynamic effects, including pressure‐driven flow, dissipative flow, buoyant‐driven flow, Marangoni flow, diffusiophoresis, and diffusioosmosis.

**Figure 11:**
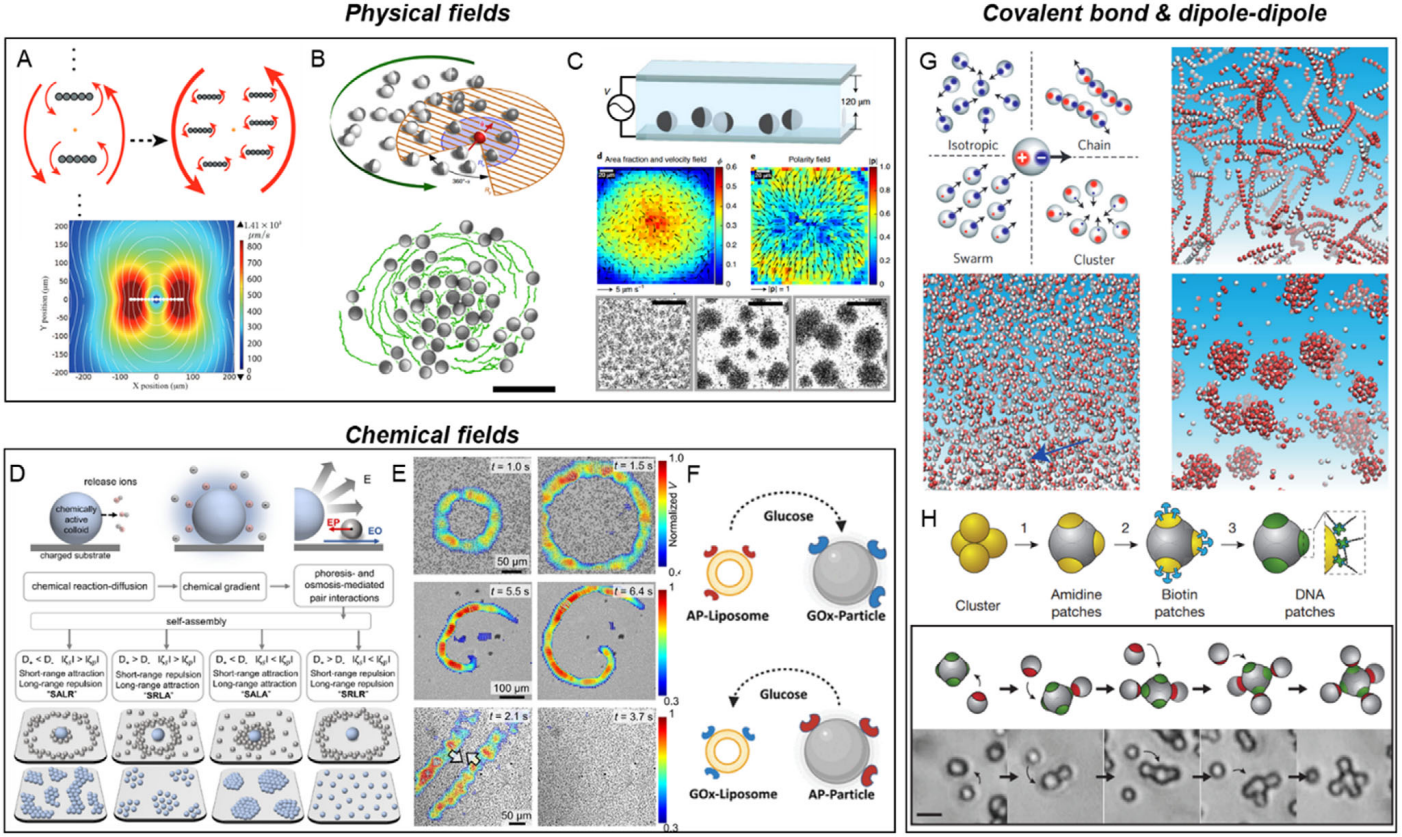
Transmitting between AAM agents. A) Local flow induced by a rotating particle chain with a rotating magnetic field (f = 5 Hz). The gray circles indicate paramagnetic nanoparticles, and the vortex flows that nanoparticle chains induce are represented with the red arrows. Reproduced with permission.^[^ ^308^ ^]^ Copyright 2019, American Chemical Society. B) An active particle is stimulated by NIR, senses the positions and orientations of neighbours, and induces a counterclockwise rotating swirl. Reproduced with permission.^[^ ^328^ ^]^ Copyright 2024, American Chemical Society. C) Cluster of SiO_2_‐Ti APs induced by ICEP, under an AC field. The bottom includes time‐averaged local area fraction, along with the velocity field and polarity field in a cluster. Reproduced with permission.^[^ ^314^ ^]^ Copyright 2020, Cambridge University Press. D) A conceptual framework for understanding the pairwise interactions and assembled patterns of chemically active colloids. The cations and anions diffuse at different rates, leading to an electric field. Electrically charged colloids nearby respond to this electric field via electrophoresis and electroosmosis. Reproduced with permission.^[^ ^320^ ^]^ Copyright 2021, Springer Nature. E) Evolution of target waves, spiral waves, and the annihilation of two colloidal waves traveling in opposite directions. Reproduced with permission.^[^ ^322^ ^]^ Copyright 2024, American Chemical Society. F) AP‐attached AAMs transfer the glucose‐6‐phosphate (G6P) signal into a glucose signal and induce the enhanced diffusion of GOx‐attached AAMs. Reproduced with permission.^[^ ^329^ ^]^ Copyright 2016, Springer Nature. G) Program dual electric charges shifted from the sphere center onto opposing hemispheres and the induced 3D simulated structures of active chains, swarms, and clusters. Reproduced with permission.^[^ ^54^ ^]^ Copyright 2016, Springer Nature. H) Specific directional bonding between colloidal atoms induced by DNA‐functionalized patches in colloids. Reproduced with permission.^[^ ^326^ ^]^ Copyright 2023, American Association for the Science.

Self‐generated physical fields, such as pressure, tension, density, temperature, and induced charges, are common in AAM systems. For instance, the application of a rotating magnetic field to nanoparticle chains induces mechanical rotation, creating fluid vortices. These vortices interact over long ranges via hydrodynamic coupling, forming coordinated microswarms, with behavior modulated by magnetic field parameters like rotational frequency and direction (**Figure** **11**A).^[^ ^17^, ^18^, ^308^ ^]^ At shorter distances, hydrodynamic repulsion between similar vortices further refines collective dynamics.^[^ ^309^ ^]^ In the context of tension, typically, micellar solubilization is a transport process occurring in surfactant‐stabilized emulsions that can lead to Marangoni flow and droplet motility. Ciera et al. controlled the direction of interfacial tension and further attractive or repulsive Marangoni flows, by manipulating the oil chemical structure, nonionic surfactant structure, and surfactant concentration.^[^ ^310^ ^]^ Regarding density, enzymatic reactions generating gas can create density gradients between product‐rich particles and the surrounding media, transmitting chemical signals into buoyancy‐driven flows and inducing collective swarming akin to bioconvection.^[^ ^311^ ^]^ Photothermal effects similarly induce slight density variations in the surrounding fluid, amplifying and transmitting signals across populations (Figure 11B).^[^ ^312^, ^313^ ^]^ Induced‐charge electroosmosis (ICEO), driven by AC electric fields, generates directional flows around particles, facilitating attractive interactions between them (Figure 11C).^[^ ^314^ ^]^

Self‐generated chemical fields, whether ionic or non‐ionic, significantly influence AAM dynamics and interactions. In diffusiophoresis‐powered AAMs, which rely on natural product gradients without electrodynamic effects, long‐range hydrodynamic flows emerge, effectively behaving as Stokeslets^[^ ^315^ ^]^ that induce local attractions.^[^ ^316^ ^]^ Additionally, imbalances in ion diffusion create localized electric fields, further enabling electrohydrodynamic effects.^[^ ^317^ ^]^ For example, ultraviolet light applied to TiO_2_ particles with rich surface groups generates ionic fields, resulting in diffusiophoresis and diffusioosmosis, which drive long‐range particle attraction.^[^ ^318^ ^]^ This interaction, in mixed particle systems, can be controlled by dye‐sensitized TiO_2_ colloids with specific light wavelengths, enabling spectral‐sensitive signal modulation.^[^ ^319^ ^]^ A recent conceptual framework by Wei Wang's group describes the competition and coupling between diffusiophoresis and diffusioosmosis under self‐generated ionic fields, providing insights into particle interactions (Figure 11D).^[^ ^320^ ^]^

Additionally, in terms of a hierarchical system, Wu et al. enhanced signal transmission by coupling self‐propelled ZnO nanorods with sulfonated polystyrene through a simple ion‐exchange reaction.^[^ ^321^ ^]^ This interaction amplifies applied fields, increasing the efficiency of quorum decision‐making to accumulate at regions of highest curvature. Beyond these cumulative fields, non‐linear reactions involving activators and inhibitors can transmit signals via traveling chemical waves (Figure 11E).^[^ ^322^ ^]^

Chemical fields also influence signal transmission through surface energy variations. Enzyme‐coated active particles, for example, convert applied fields into product gradients, affecting collective behavior via the Hofmeister effect.^[^ ^268^ ^]^ Yu‐Ching et al. utilized a liposome coated with glucose oxidase (GOx) and acid phosphatase to realize the transferring and transmitting of signals.^[^ ^323^ ^]^ Phosphatase catalyzes glucose‐6‐phosphate (G6P) into glucose, which is then converted to gluconic acid and hydrogen peroxide by GOx, consequently, it activates the enhanced diffusion of GOx‐based liposome (Figure 11F).

Besides long‐range interactions, short‐range interactions, including covalent bonding and dipole–dipole interactions, also play a critical role in signal transmission. Unlike the cumulative fields in the diffusiophoresis, electrophoresis relies on asymmetric surface ion distributions to propagate signals with dipole–dipole interactions.^[^ ^324^ ^]^ Polarization under electromagnetic fields also transmits signals via dipole–dipole interactions (Figure 11G). Functionalized colloids, incorporating chemistry groups or DNA with sticky single‐stranded ends, facilitate signal propagation through specific interactions, further extending communication capabilities (Figure 11H).^[^ ^325^, ^326^, ^327^ ^]^

The efficacy of signal transmission in AAMs is determined by factors such as range, strength, and selectivity. Electromagnetic forces dominate at the microscale due to their intensity, but are limited in range and selectivity. Ionic fields, common in chemically active particles, are prone to screening in biological environments due to high ionic strength. Non‐ionic fields, while less frequent, offer superior selectivity and resistance to screening. Hydrodynamic transmission, effective over distances up to 100 µm, is relatively ion‐insensitive but lacks selectivity. Future research should prioritize strategies to amplify signals, improve interspecies communication among active matter, and optimize the balance between transmission range, strength, and selectivity.

#### 5.2.3 Executing

**Figure 12:**
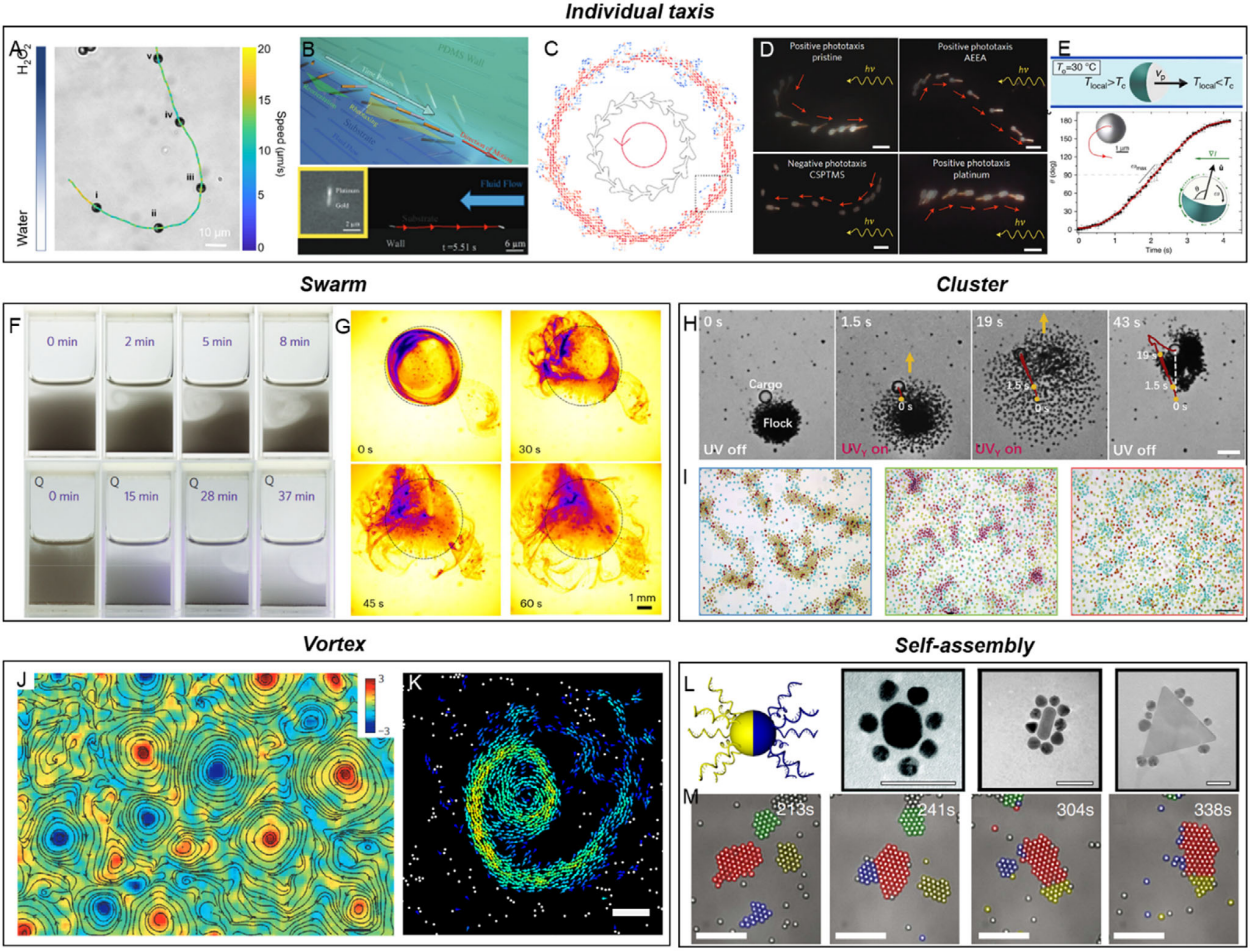
Performance execution of individual and collective AAMs. A) Chemotaxis of Cu@SiO_2_ toward high H_2_O_2_ concentrations. Reproduced with permission.^[^ ^255^ ^]^ Copyright 2022, Wiley‐VCH GmbH. B) Rheotaxis of a bimetallic nanorod against the applied flow. Reproduced with permission.^[^ ^304^ ^]^ Copyright 2022, Springer Nature. C) Haptotaxis of micromotors and the circular flow inside periodic ratchet‐like structures. Reproduced with permission.^[^ ^301^ ^]^ Copyright 2021, Springer Nature. D) Programmable phototaxis of an individual TiO_2_ Janus nanotree. Reproduced with permission.^[^ ^330^ ^]^ Copyright 2016, Springer Nature. E) Temperature gradient‐guided directional motion of individual AAM. Reproduced with permission.^[^ ^331^ ^]^ Copyright 2022, Wiley‐VCH GmbH. F) Programmable phototaxis of TiO_2_ Janus nanotree swarms. Reproduced with permission.^[^ ^330^ ^]^ Copyright 2016, Springer Nature. G) Swarming behaviors of urease‐based AAMs in the urea solution. Reproduced with permission.^[^ ^9^ ^]^ Copyright 2024, Springer Nature. H) Cluster of TiO_2_ AAMs transports a large SiO_2_ cargo (10 mm in size) in open space. Reproduced with permission.^[^ ^318^ ^]^ Copyright 2023, Springer Nature. I) Spectrally tunable segregation of a ternary mixture composed of L0, LEG4, and SQ2 sensitized TiO_2_ colloids. Reproduced with permission.^[^ ^319^ ^]^ Copyright 2024, American Chemical Society. J) Multiple vortices of metal–dielectric Janus colloids subjected to perpendicular AC electric fields. Reproduced with permission.^[^ ^54^ ^]^ Copyright 2016, Springer Nature. K) Spiral vortex of Quincke rollers immersed in a conducting fluid subjected to a vertical DC electric field. Reproduced with permission.^[^ ^335^ ^]^ Copyright 2013, American Association for the Science. L) Control over the spatial distribution of surface‐bound DNA strands allows for particles that are asymmetric and can form core‐satellite clusters. Reproduced with permission.^[^ ^327^ ^]^ Copyright 2020, Springer Nature. M) Self‐assembly of light‐activated AAMs into living crystals. Reproduced with permission.^[^ ^336^ ^]^ Copyright 2023, American Chemical Society.

As previously discussed (Section 2.2.2), AAMs are driven by asymmetric fields, either self‐generated or externally applied. Upon sensing and transmitting signals, these fields around AAMs can be activated or modified, ultimately influencing the dynamical behaviors of the system. We summarize the resultant dynamical behaviors under various fields, encompassing both individual (**Table** **5**) and collective (**Table** **6**) responses. For individual behaviors, signal transmission primarily relies on the diffusion‐convection of initial signals or reaction products, without inducing additional interactions between AAMs. Similar to natural active matter, individual agents exhibit a range of tactic responses to stimuli, including chemotaxis (**Figure** **12**A),^[^ ^255^ ^]^ rheotaxis (Figure 12B),^[^ ^304^ ^]^ haptotaxis (Figure 12C),^[^ ^301^ ^]^ phototaxis (Figure 12D),^[^ ^330^ ^]^ thermotaxis (Figure 12E),^[^ ^331^ ^]^ and magnetotaxis, which have been comprehensively reviewed elsewhere.^[^ ^253^, ^332^, ^333^, ^334^ ^]^ Simply, for AAMs with asymmetric structure, they usually experience an aligning torque **M**, which orients the AAMs temporally or continuously. Isotropic AAMs may produce propulsion forces whose direction is solely determined by the applied field, regardless of their Brownian rotations.^[^ ^253^ ^]^

**Table 5:** Interaction between individual AAMs with fields.

Field	Information	Active matter	Sensing	Executing
Urea	2.94 mm mm^−1^	Au‐urease Janus nanoparticle (d = 90 nm)^[^ ^337^ ^]^	Differences of reaction rates	Positive chemotaxis with directionality of 0.14
	1.25 mm µm^−1^	Liposome‐urease nanoparticle (d = 123 nm)^[^ ^268^ ^]^	Energy state of surface	Negative chemotaxis with chemotactic distance of 11 µm
H_2_O_2_	16.6 mm mm^−1^	Liposome‐catalase nanoparticle (d = 123 nm)^[^ ^268^ ^]^	Energy state of surface	Positive chemotaxis with chemotactic distance of 5 µm
	26 mm mm^−1^	Cu‐SiO_2_ Janus microparticle (d = 5 µm)^[^ ^255^ ^]^	Differences of reaction rates and different phoretic mobility on the inert/active surface	Positive chemotaxis with velocity of 10 µm s^−1^
CO_2_	0.5 µm µm^−1^	ZnO/SiO_2_ Janus microparticle (d = 2.5 µm)^[^ ^265^ ^]^	Differences of reaction rates	Positive chemotaxis with chemotaxis index of 0.21
pH	20 mm^−1^	Flask‐like carbonaceous nanomotors loading Fe_3_O_4_ nanoparticles^[^ ^338^ ^]^	Differences of reaction rates and mechanisms	Negative chemotaxis
	–	Rotary FoF1‐ATPase motor‐propelled flasklike pentosan microparticle^[^ ^264^ ^]^	Hydrodynamics and geometric asymmetry	Negative chemotaxis with velocity of 1.80 µm s^−1^
ATP	0.83 mm mm^−1^	Liposome‐ATPase nanoparticle (d = 123 nm)^[^ ^268^ ^]^	Energy state of surface	Positive chemotaxis with chemotactic distance of 11.4 µm
Glucose	880 mm cm^−1^	Au‐GOx Janus nanoparticle (d = 25 nm)^[^ ^339^ ^]^	–	Positive chemotaxis
	1 m cm^−1^	PMPC‐PDPA/PEO‐PBO nanoparticle loaded with catalase and GOx (d = 50 nm)^[^ ^262^ ^]^	Differences of reaction rates	Positive chemotaxis with velocity of 16 µm s^−1^
ROS/NOS	4.06 µM cm^−1^	PCBMA@L‐arginine nanoparticle (d = 220 nm)^[^ ^340^ ^]^	Differences of reaction rates	Positive chemotaxis with velocity of 1.3 µm s^−1^, and chemotaxis index of 0.8
GSH	1.92 µg ml^−1^	PMG‐ glutathione hydrolase γ‐ glutamyltransferase nanoparticle (d = 280 nm)^[^ ^263^ ^]^	Differences of reaction rates	Positive chemotaxis with velocity of 17 µm s^−1^
Light	UV light (λ = 365 nm), 1 W cm^−2^	TiO_2_ microparticle (d = 1.2 µm)^[^ ^60^ ^]^	Differences of light absorbance	Negative phototaxis with velocity of 12.8 µm s^−1^
	UV light (λ = 365 nm)	TiO_2_/Si nanotree^[^ ^330^ ^]^	Electrostatic force and differences of light absorbance	Positive phototaxis and negative phototaxis with velocity of 0.44–0.83 mm^3^ J^−1^
Flow	4 µm s^−1^	Spherical 3‐methacryloxypropyl trimethoxysilane polymer (TPM) containing a hematite cube^[^ ^303^ ^]^	Pressure	Positive rheotaxis with velocity of 2 µm s^−1^
	14 µm s^−1^	SiO_2_‐Pt Janus microparticle (d = 2.5 µm)^[^ ^307^ ^]^	Pressure and hydrodynamics	Cross‐stream migration
Electromagnetism	50 Gauss	Bowl‐shaped polymersomes loaded with Pt and Ni^[^ ^341^ ^]^	Magnetization	Positive magnetotaxis with velocity of 13.1 µm s^−1^
	1–10 V cm^−1^	Negatively charged quantum dots^[^ ^167^ ^]^	Electrostatic force	Positive galvanotaxis
Topology	Step‐like submicrometer topographical features	Pt‐SiO_2_ Janus microparticle (d = 5 µm)^[^ ^299^ ^]^	Chemical activity and associated hydrodynamic effect	Get close to and move along with the step
	Linear ratchet structure	Pt‐SiO_2_ Janus microparticle (d = 5 µm)^[^ ^301^ ^]^	Chemical activity, associated hydrodynamic effect	Move along the chirality and accumulate in the final corner

**Table 6:** Interaction between collective AAMs with fields.

Field	Information	Active matter	Sensing	Transmitting	Executing
pH	4.83 mm^−1^	Active urease droplets (d = 100 µm)^[^ ^342^, ^343^ ^]^	Differences of interfacial tension	Self‐generated field of OH^‐^	Cluster and positive chemotaxis with velocity of 5 µm s^−1^
Glucose 6 phosphate	1 mm	Liposomes with acid phosphatase and glucose oxidase (d ≈200–300 nm)^[^ ^323^ ^]^	Differences of reaction rates	Self‐generated field of glucose	Enhanced diffusion of liposome loaded with GOx
Light	UV light (λ = 365 nm), 1 W cm^−2^	TiO_2_ microparticle (d = 1.2 µm)^[^ ^318^ ^]^	Differences of light absorbance	Hydrodynamics	Cluster and negative phototaxis with velocity around 10 µm s^−1^
	UV light (λ = 365 nm), 43 mW cm^−2^	PMMA‐Ag Janus microparticle (d = 2 µm)^[^ ^322^ ^]^	Differences of light absorbance and hydrodynamics	Self‐generated field of OH^‐^	Chemical waves
	Light with different wavelength and ratios of power rate	Dye‐sensitized TiO_2_ microparticle^[^ ^319^ ^]^	Differences of light absorbance	Phoretic hydrodynamics	Selective phase segregation of cluster
Electromagnetism	Rotating magnetic field with H = 6000 A m^−1^ and f = 30 Hz	Peanut‐shaped hematite colloidal microparticle^[^ ^56^ ^]^	Magnetizing	Dipole–dipole interaction and hydrodynamics	Transfer between vortex, chain and ribbon
	Alternating electric field with frequency: 20–50 kHz	SiO_2_‐metal microparticle (d = 3 µm)^[^ ^54^ ^]^	Polarization	ICEP	Transfer between cluster, chain and swarm
	Alternating electric field	SiO_2_ and TiO_2_ microparticle^[^ ^61^ ^]^	Polarization	ICEP	Formation and directional motion of cluster, enhanced positive and negative phototaxis
Acoustic field	10 Vpp with frequency: 618 kHz	Au‐Pt nanorods^[^ ^344^ ^]^	Bjerknes forces	Secondary Bjerknes force	Cluster
Topology	Curvature	ZnO microrods and sulfonated polystyrene microparticles^[^ ^321^ ^]^	Hydrodynamics	Hydrodynamics	Accumulation in the corner with high curvature

Additionally, fields can also activate the collective behaviors, but with ignorable interactions between each other, like swarms (Figure 12F,G). For example, the individual agents showing positive/negative phototaxis in 2D, also exhibit phototactic and antigravity swarming in 3D, like eukaryotic phototactic algae, and it can be controlled by the charge of the tree head.^[^ ^330^ ^]^ Furthermore, interactions between agents can also be modulated by fields, inducing collective behaviors like cluster (Figure 12H,I), vortex (Figure 12J,K), and self‐assembly (Figure 12L,M). Notably, the effects of interactions between AAMs under the fields, especially the chemical fields, are usually ignored in the existing research, which is one of the potential hotspots in the future.

## 6 Summary and Outlook

This review provides a comprehensive overview of endogenous fields and their interactions with active matter. We begin by introducing the propulsion mechanisms of both NAMs and AAMs, emphasizing the pivotal role of asymmetric fields in active matter mobility. A critical section of our discussion synthesizes information on endogenous fields, such as oxygen, pH, ROS, topology, and electrical gradients, in scenes of AAM applications like tumors, skin, and inflammation. Furthermore, we explore the general principles governing active matter‐field interactions, focusing on the processes of sensing, transmission, and execution. Finally, we compare NAMs and AAMs, elucidating how NAM‐inspired designs can inform the advancement of AAMs and identifying persistent challenges in the field.

The dynamics of AAMs under gradient fields, such as high motility, targeted diffusion, and emergent collective behaviors, have attracted considerable attention in both principle and applied research.^[^ ^345^, ^346^, ^347^, ^348^ ^]^ Nevertheless, artificial systems still exhibit notable shortcomings compared to NAMs. 1) AAM sensors generally lack sensitivity, responding only to strong field intensities and a limited range of field types. NAMs exhibit dynamic coordination across multiple interacting fields, AAM dynamics are generally modeled as interactions with a single or a few fields. 2) Most studies on AAM dynamics under fields, particularly chemical fields, are confined to single AAM agent, leaving a significant knowledge gap in understanding collective behaviors. 3) Moreover, signal conversion and amplification, hallmarks of NAMs, are frequently ignored in AAM research.^[^ ^61^, ^318^, ^321^ ^]^ 4) AAMs show low energy‐conversion efficiencies, typically ranging between 10^−9^ and 10^−4^, far below the 10^−1^ and 10^0^ efficiencies observed in NAMs.^[^ ^349^, ^350^ ^]^ 5) Unlike NAMs, which separate motile and sensory functions, AAM designs often integrate these into a single unit, limiting adaptability. While. Future advancements in AAMs should prioritize improving sensor sensitivity, enhancing signal conversion, amplification, and synergy, increasing actuator efficiency, and adopting modular designs for functional components.

Chemotaxis remains a central focus in AAM‐field interactions, drawing attention for both its fundamental principles and potential applications.^[^ ^340^, ^351^, ^352^ ^]^ Despite these advancements, unresolved issues persist: 1) The absence of standardized parameters for describing field interactions and behaviors hinders data comparability. Current metrics, such as chemotactic velocity, index, and directionality, primarily target single particles, often neglecting field intensity and spatial range—key factors for comprehensive evaluations. 2) Most studies examine interactions with single fields, failing to simulate the complexity of real‐world environments. Future research should account for the competition and coordination of multiple, dynamic, and heterogeneous fields. 3) The absence of explorations about the AAMs‐fields coupling effect, rather than a one‐way effect. And the discussions of chemotaxis in porous and crowded environments, instead of open environments, provide more valuable references for applications. 4) While chemotaxis has demonstrated promise in specific applications, its reliability in practical settings, particularly in vivo, remains uncertain. Biological environments are highly complex, with various sources of “noise”, such as strong convection and confinement within the extracellular matrix, which may disrupt these weak taxis responses. Moreover, the field intensities encountered in vivo are often weaker than those tested in vitro. Although some studies have explored the potential of chemotaxis in vivo, strong control groups are lacking to isolate its effects from other influencing factors, such as convection, electrostatic interactions, and particle‐cell interactions.

Another critical challenge—and opportunity—in AAM‐field interaction studies lies in imaging and analytical technologies. Current imaging techniques often yield inconsistent quantifications of the same fields. Many imaging technologies, such as electrode‐based systems, provide only point‐specific data. The development of real‐time, comprehensive mapping methods and descriptors is essential to capturing the complexity of endogenous fields.

AAMs offer invaluable models for understanding natural phenomena, and their unique non‐equilibrium properties hold potential for transformative breakthroughs in biomedical, environmental, and sensing applications. By leveraging their interactions with endogenous fields, we can better harness these non‐equilibrium processes for practical use. Looking forward, we anticipate that the broad implementation of AAM systems across diverse applications could soon become a reality.

## Conflict of Interest

The authors declare no conflict of interest.

## References

[1] D. Needleman , Z. Dogic , Nat. Rev. Mater. 2017, 2, 17048.

[2] P. Romanczuk , M. Bär , W. Ebeling , B. Lindner , L. Schimansky‐Geier , Eur. Phys. J. Spec. Top. 2012, 202, 1.

[3] S. Chen , D. E. Fan , P. Fischer , A. Ghosh , K. Göpfrich , R. Golestanian , H. Hess , X. Ma , B. J. Nelson , T. Patiño Padial , J. Tang , K. Villa , W. Wang , L. Zhang , A. Sen , S. Sánchez , Nat. Nanotechnol. 2025, 20, 990. 40750672 10.1038/s41565-025-01962-9

[4] X. Ju , C. Chen , C. M. Oral , S. Sevim , R. Golestanian , M. Sun , N. Bouzari , X. Lin , M. Urso , J. S. Nam , Y. Cho , X. Peng , F. C. Landers , S. Yang , A. Adibi , N. Taz , R. Wittkowski , D. Ahmed , W. Wang , V. Magdanz , M. Medina‐Sánchez , M. Guix , N. Bari , B. Behkam , R. Kapral , Y. Huang , J. Tang , B. Wang , K. Morozov , A. Leshansky , et al., ACS Nano 2025, 19, 24174. 40577644 10.1021/acsnano.5c03911 PMC12269370

[5] M. Das , C. F. Schmidt , M. Murrell , Soft Matter 2020, 16, 7185. 32724969 10.1039/d0sm90137g

[6] T. Patiño Padial , S. Chen , A. C. Hortelão , A. Sen , S. Sánchez , Nat. Rev. Mater. 2025, 10.1038/s41578-025-00818-x.

[7] A. C. Hortelao , C. Simo , M. Guix , S. Guallar‐Garrido , E. Julian , D. Vilela , L. Rejc , P. Ramos‐Cabrer , U. Cossio , V. Gomez‐Vallejo , T. Patino , J. Llop , S. Sanchez , Sci. Rob. 2021, 6, abd2823. 10.1126/scirobotics.abd2823 34043548

[8] Q. Wang , Q. Wang , Z. Ning , K. F. Chan , J. Jiang , Y. Wang , L. Su , S. Jiang , B. Wang , B. Y. Ip , H. Ko , T. W. Leung , P. W. Chiu , S. C. Yu , L. Zhang , Sci. Rob. 2024, 9, adh1978. 10.1126/scirobotics.adh1978 38381838

[9] C. Simó , M. Serra‐Casablancas , A. C. Hortelao , V. Di Carlo , S. Guallar‐Garrido , S. Plaza‐García , R. M. Rabanal , P. Ramos‐Cabrer , B. Yagüe , L. Aguado , L. Bardia , S. Tosi , V. Gómez‐Vallejo , A. Martín , T. Patiño , E. Julián , J. Colombelli , J. Llop , S. Sánchez , Nat. Nanotechnol. 2024, 19, 554. 38225356 10.1038/s41565-023-01577-y PMC11026160

[10] L. Chen , H. Yuan , S. Chen , C. Zheng , X. Wu , Z. Li , C. Liang , P. Dai , Q. Wang , X. Ma , X. Yan , ACS Appl. Mater. Interfaces 2021, 13, 31226. 34176260 10.1021/acsami.1c03595

[11] J. Yang , Y. Liu , J. Li , M. Zuo , W. Li , N. Xing , C. Wang , T. Li , Appl. Mater. Today 2021, 25, 101190.

[12] S. Jeon , S. Kim , S. Ha , S. Lee , E. Kim , S. Y. Kim , S. H. Park , J. H. Jeon , S. W. Kim , C. Moon , B. J. Nelson , J. Kim , S.‐W. Yu , H. Choi , Sci. Rob. 2019, 4, aav4317.

[13] H. Ceylan , I. C. Yasa , O. Yasa , A. F. Tabak , J. Giltinan , M. Sitti , ACS Nano 2019, 13, 3353. 30742410 10.1021/acsnano.8b09233 PMC6728090

[14] Q. Wang , X. Du , D. Jin , L. Zhang , ACS Nano 2022, 16, 604. 34985859 10.1021/acsnano.1c07830

[15] Q. Wang , K. F. Chan , K. Schweizer , X. Du , D. Jin , S. C. H. Yu , B. J. Nelson , L. Zhang , Sci. Adv. 2021, 7, abe5914. 10.1126/sciadv.abe5914 PMC7909881 33637532

[16] Q. Wang , N. Xiang , J. Lang , B. Wang , D. Jin , L. Zhang , Adv. Intell. Syst. 2024, 6, 2300108.

[17] J. Yu , D. Jin , K.‐F. Chan , Q. Wang , K. Yuan , L. Zhang , Nat. Commun. 2019, 10, 5631. 31822669 10.1038/s41467-019-13576-6 PMC6904566

[18] D. Jin , K. Yuan , X. Du , Q. Wang , S. Wang , L. Zhang , Adv. Mater. 2021, 33, 2100070. 10.1002/adma.202100070 34337789

[19] D. Jin , Q. Wang , K. F. Chan , N. Xia , H. Yang , Q. Wang , S. C. H. Yu , L. Zhang , Sci. Adv. 2023, 9, adf9278. 10.1126/sciadv.adf9278 PMC10181194 37172097

[20] A. Liu , Q. Wang , Z. Zhao , R. Wu , M. Wang , J. Li , K. Sun , Z. Sun , Z. Lv , J. Xu , H. Jiang , M. Wan , D. Shi , C. Mao , ACS Nano 2021, 15, 13339. 34324304 10.1021/acsnano.1c03177

[21] J. Mujtaba , J. Liu , K. K. Dey , T. Li , R. Chakraborty , K. Xu , D. Makarov , R. A. Barmin , D. A. Gorin , V. P. Tolstoy , G. Huang , A. A. Solovev , Y. Mei , Adv. Mater. 2021, 33, 2007465. 10.1002/adma.202007465 33893682

[22] M. Wan , H. Chen , Q. Wang , Q. Niu , P. Xu , Y. Yu , T. Zhu , C. Mao , J. Shen , Nat. Commun. 2019, 10, 966. 30814497 10.1038/s41467-019-08670-8 PMC6393443

[23] T. Lämmermann , Nat. Biomed. Eng. 2017, 1, 0100.

[24] G. M. Allen , A. Mogilner , J. A. Theriot , Curr. Biol. 2013, 23, 560. 23541731 10.1016/j.cub.2013.02.047 PMC3718648

[25] M. C. Marchetti , J. F. Joanny , S. Ramaswamy , T. B. Liverpool , J. Prost , M. Rao , R. A. Simha , Rev. Mod. Phys. 2013, 85, 1143.

[26] R. Iino , K. Kinbara , Z. Bryant , Chem. Rev. 2020, 120, 1. 31910626 10.1021/acs.chemrev.9b00819

[27] H. Ueno , T. Suzuki , K. Kinosita , M. Yoshida , Proc. Natl. Acad. Sci. USA 2005, 102, 1333. 15668386 10.1073/pnas.0407857102 PMC545493

[28] J. A. Spudich , Nat. Rev. Mol. Cell Biol. 2001, 2, 387. 11331913 10.1038/35073086

[29] S. M. Block , Biophys. J. 2007, 92, 2986. 17325011 10.1529/biophysj.106.100677 PMC1852353

[30] G. Bhabha , G. T. Johnson , C. M. Schroeder , R. D. Vale , Trends Biochem. Sci. 2016, 41, 94. 26678005 10.1016/j.tibs.2015.11.004 PMC4706479

[31] K. Zegadło , M. Gieroń , P. Żarnowiec , K. Durlik‐Popińska , B. Kręcisz , W. Kaca , G. Czerwonka , IJMS 2023, 24, 1707. 36675220 10.3390/ijms24021707 PMC9864740

[32] P. Friedl , S. Borgmann , E.‐B. Bröcker , J. Leukocyte Biol. 2001, 70, 491. 11590185

[33] G. Charras , E. Paluch , Nat. Rev. Mol. Cell Biol. 2008, 9, 730. 18628785 10.1038/nrm2453

[34] G. T. Charras , J. C. Yarrow , M. A. Horton , L. Mahadevan , T. J. Mitchison , Nature 2005, 435, 365. 15902261 10.1038/nature03550 PMC1564437

[35] P. Friedl , K. Wolf , Nat. Rev. Cancer 2003, 3, 362. 12724734 10.1038/nrc1075

[36] V. Sanz‐Moreno , G. Gadea , J. Ahn , H. Paterson , P. Marra , S. Pinner , E. Sahai , C. J. Marshall , Cell 2008, 135, 510. 18984162 10.1016/j.cell.2008.09.043

[37] S. Even‐Ram , K. M. Yamada , Curr. Opin. Cell Biol. 2005, 17, 524. 16112853 10.1016/j.ceb.2005.08.015

[38] T. Kudernac , N. Ruangsupapichat , M. Parschau , B. Maciá , N. Katsonis , S. R. Harutyunyan , K.‐H. Ernst , B. L. Feringa , Nature 2011, 479, 208. 22071765 10.1038/nature10587

[39] K. Zhu , G. Baggi , S. J. Loeb , Nat. Chem. 2018, 10, 625. 29713030 10.1038/s41557-018-0040-9

[40] G. J. Simpson , M. Persson , L. Grill , Nature 2023, 621, 82. 37673992 10.1038/s41586-023-06384-y

[41] W. Siti , H. L. Too , T. Anderson , X. R. Liu , I. Y. Loh , Z. Wang , Sci. Adv. 2023, 9, adi8444. 10.1126/sciadv.adi8444 PMC10516491 37738343

[42] A.‐K. Pumm , W. Engelen , E. Kopperger , J. Isensee , M. Vogt , V. Kozina , M. Kube , M. N. Honemann , E. Bertosin , M. Langecker , R. Golestanian , F. C. Simmel , H. Dietz , Nature 2022, 607, 492. 35859200 10.1038/s41586-022-04910-y PMC9300469

[43] J. L. Anderson , Annu. Rev. Fluid Mech. 1989, 21, 61.

[44] S. Sánchez , L. Soler , J. Katuri , Angew. Chem., Int. Ed. 2015, 54, 1414. 10.1002/anie.201406096 25504117

[45] G. Gallino , F. Gallaire , E. Lauga , S. Michelin , Adv. Funct. Mater. 2018, 28, 1800686.

[46] D. Wang , D. Guan , J. Su , X. Zheng , G. Hu , Phys. Fluids 2021, 33, 122004.

[47] C. Zhou , H. P. Zhang , J. Tang , W. Wang , Langmuir 2018, 34, 3289. 29436833 10.1021/acs.langmuir.7b04301

[48] K. Feng , J. C. Ureña Marcos , A. K. Mukhopadhyay , R. Niu , Q. Zhao , J. Qu , B. Liebchen , Adv. Sci. 2023, 10, 2300866. 10.1002/advs.202300866 PMC10520641 37526332

[49] Y. Huang , C. Wu , J. Dai , B. Liu , X. Cheng , X. Li , Y. Cao , J. Chen , Z. Li , J. Tang , J. Am. Chem. Soc. 2023, 145, 19945. 37641545 10.1021/jacs.3c06322

[50] A. M. Boymelgreen , T. Balli , T. Miloh , G. Yossifon , Nat. Commun. 2018, 9, 760. 29472542 10.1038/s41467-018-03086-2 PMC5823901

[51] W. Wang , S. Li , L. Mair , S. Ahmed , T. J. Huang , T. E. Mallouk , Angew. Chem., Int. Ed. 2014, 53, 3201. 10.1002/anie.201309629 PMC4069361 24677393

[52] S. Ahmed , W. Wang , L. Bai , D. T. Gentekos , M. Hoyos , T. E. Mallouk , ACS Nano 2016, 10, 4763. 26991933 10.1021/acsnano.6b01344

[53] A. Aghakhani , O. Yasa , P. Wrede , M. Sitti , Proc. Natl. Acad. Sci. USA 2020, 117, 3469. 32015114 10.1073/pnas.1920099117 PMC7035478

[54] J. Yan , M. Han , J. Zhang , C. Xu , E. Luijten , S. Granick , Nat. Mater. 2016, 15, 1095. 27400388 10.1038/nmat4696

[55] F. Nadal , E. Lauga , Phys. Fluids 2014, 26, 082001.

[56] H. Xie , M. Sun , X. Fan , Z. Lin , W. Chen , L. Wang , L. Dong , Q. He , Sci. Rob. 2019, 4, aav8006. 10.1126/scirobotics.aav8006 33137748

[57] M. Driscoll , B. Delmotte , M. Youssef , S. Sacanna , A. Donev , P. Chaikin , Nat. Phys. 2017, 13, 375.

[58] Z. Zhang , A. Sukhov , J. Harting , P. Malgaretti , D. Ahmed , Nat. Commun. 2022, 13, 7347. 36446799 10.1038/s41467-022-35078-8 PMC9708833

[59] L. Yang , L. Zhang , Annu. Rev. Control Robot. Auton. Syst. 2021, 4, 509.

[60] C. Chen , F. Mou , L. Xu , S. Wang , J. Guan , Z. Feng , Q. Wang , L. Kong , W. Li , J. Wang , Q. Zhang , Adv. Mater. 2017, 29, 1603374. 10.1002/adma.201603374 27748536

[61] X. Liang , F. Mou , Z. Huang , J. Zhang , M. You , L. Xu , M. Luo , J. Guan , Adv. Funct. Mater. 2020, 30, 1908602.

[62] W. Li , C. Wu , Z. Xiong , C. Liang , Z. Li , B. Liu , Q. Cao , J. Wang , J. Tang , D. Li , Sci. Adv. 2022, 8, ade1731. 10.1126/sciadv.ade1731 PMC9645706 36351008

[63] C. Bechinger , R. Di Leonardo , H. Löwen , C. Reichhardt , G. Volpe , G. Volpe , Rev. Mod. Phys. 2016, 88, 045006.

[64] J. Elgeti , R. G. Winkler , G. Gompper , Rep. Prog. Phys. 2015, 78, 056601. 25919479 10.1088/0034-4885/78/5/056601

[65] J. L. Moran , J. D. Posner , Annu. Rev. Fluid Mech. 2017, 49, 511.

[66] B. Wang , S. Handschuh‐Wang , J. Shen , X. Zhou , Z. Guo , W. Liu , M. Pumera , L. Zhang , Adv. Mater. 2023, 35, 2205732. 10.1002/adma.202205732 36113864

[67] W. Wang , X. Lv , J. L. Moran , S. Duan , C. Zhou , Soft Matter 2020, 16, 3846. 32285071 10.1039/d0sm00222d

[68] K. J. Rao , F. Li , L. Meng , H. Zheng , F. Cai , W. Wang , Small 2015, 11, 2836. 25851515 10.1002/smll.201403621

[69] R. P. Feynman , R. B. Leighton , M. L. Sands , The Feynman Lectures on Physics Vol. II Ch. 1: Electromagnetism, https://www.feynmanlectures.caltech.edu/II_01.html (accessed: November 2024).

[70] Y. Chao , Z. Liu , Nat. Rev. Bioeng. 2023, 1, 125.

[71] N. Campillo , B. Falcones , J. Otero , R. Colina , D. Gozal , D. Navajas , R. Farré , I. Almendros , Front. Oncol. 2019, 9, 43. 30788287 10.3389/fonc.2019.00043 PMC6373430

[72] S. Auerswald , S. Schreml , R. Meier , A. Blancke Soares , M. Niyazi , S. Marschner , C. Belka , M. Canis , F. Haubner , Radiat. Oncol. 2019, 14, 199. 31711506 10.1186/s13014-019-1413-y PMC6849199

[73] H.‐C. Shih , T.‐A. Lee , H.‐M. Wu , P.‐L. Ko , W.‐H. Liao , Y.‐C. Tung , Sci. Rep. 2019, 9, 8234. 31160651 10.1038/s41598-019-44594-5 PMC6546762

[74] Y. Wang , R. Kim , S. S. Hinman , B. Zwarycz , S. T. Magness , N. L. Allbritton , Cell. Mol. Gastroenterol. Hepatol. 2018, 5, 440. 29675459 10.1016/j.jcmgh.2018.01.008 PMC5904029

[75] J. S. Gibson , P. I. Milner , R. White , T. P. A. Fairfax , R. J. Wilkins , Pflug. Arch. Eur. J. Physiol. 2007, 455, 563. 10.1007/s00424-007-0310-7 17849146

[76] S. Zhou , Z. Cui , J. P. G. Urban , Arthritis Rheum. 2004, 50, 3915. 15593204 10.1002/art.20675

[77] A. D. Klementiev , Z. Jin , M. Whiteley , MBio 2020, 11, 02536. 10.1128/mBio.02536-20 PMC7587442 33082251

[78] H.‐C. Flemming , J. Wingender , U. Szewzyk , P. Steinberg , S. A. Rice , S. Kjelleberg , Nat. Rev. Microbiol. 2016, 14, 563. 27510863 10.1038/nrmicro.2016.94

[79] A. M. Stock , Proc. Natl. Acad. Sci. USA 1997, 94, 10487.9380664

[80] T. Hompland , C. S. Fjeldbo , H. Lyng , Cancers 2021, 13, 499. 33525508 10.3390/cancers13030499 PMC7866096

[81] S. Matsumoto , K. Saito , H. Yasui , H. D. Morris , J. P. Munasinghe , M. Lizak , H. Merkle , J. H. Ardenkjaer‐Larsen , R. Choudhuri , N. Devasahayam , S. Subramanian , A. P. Koretsky , J. B. Mitchell , M. C. Krishna , Magn. Reson. Med. 2013, 69, 1443. 22692861 10.1002/mrm.24355 PMC3479339

[82] M. G. Espey , Free Radical Biol. Med. 2013, 55, 130. 23127782 10.1016/j.freeradbiomed.2012.10.554

[83] J. Lennox , Advantages of living in a biofilm, https://www.hypertextbookshop.com/biofilmbook/v004/r003/contents/chapters/chapter002/section003/blue/page001.html (accessed: November 2024).

[84] R. J. Gillies , N. Raghunand , M. L. Garcia‐Martin , R. A. Gatenby , IEEE Eng. Med. Biol. Mag. 2004, 23, 57. 10.1109/memb.2004.1360409 15565800

[85] D. Morone , F. D. Autilia , T. Schorn , M. Erreni , A. Doni , Sci. Rep. 2020, 10, 6289. 32286404 10.1038/s41598-020-63203-4 PMC7156395

[86] L. Gaohua , X. Miao , L. Dou , Expert Opin. Drug Metab. Toxicol. 2021, 17, 1103. 34253134 10.1080/17425255.2021.1951223

[87] S. Fulaz , D. Hiebner , C. H. N. Barros , H. Devlin , S. Vitale , L. Quinn , E. Casey , ACS Appl. Mater. Interfaces 2019, 11, 32679. 31418546 10.1021/acsami.9b09978

[88] Y. Yang , V. Sourjik , Mol. Microbiol. 2012, 86, 1482. 23078189 10.1111/mmi.12070

[89] K. M. Jones , E. A. Randtke , E. S. Yoshimaru , C. M. Howison , P. Chalasani , R. R. Klein , S. K. Chambers , P. H. Kuo , M. D. Pagel , Mol. Imaging Biol. 2017, 19, 617. 27896628 10.1007/s11307-016-1029-7 PMC6010170

[90] M. Schieber , N. S. Chandel , Curr. Biol. 2014, 24, R453. 24845678 10.1016/j.cub.2014.03.034 PMC4055301

[91] C. Shen , M. Gao , H. Chen , Y. Zhan , Q. Lan , Z. Li , W. Xiong , Z. Qin , L. Zheng , J. Zhao , J. Nanobiotechnol. 2021, 19, 395. 10.1186/s12951-021-01136-4 PMC8627084 34838028

[92] C. Nathan , A. Cunningham‐Bussel , Nat. Rev. Immunol. 2013, 13, 349. 23618831 10.1038/nri3423 PMC4250048

[93] Y. Wu , T. Guo , Y. Qiu , Y. Lin , Y. Yao , W. Lian , L. Lin , J. Song , H. Yang , Chem. Sci. 2019, 10, 7068. 31588274 10.1039/c9sc01070j PMC6676468

[94] D. Pham , U. Basu , I. Pohorilets , C. M. St Croix , S. C. Watkins , K. Koide , Angew. Chem., Int. Ed. 2020, 59, 17435. 10.1002/anie.202007104 32585075

[95] P. P. Rosias , G. J. Den Hartog , C. M. Robroeks , A. Bast , R. A. Donckerwolcke , J. W. Heynens , J. Suykerbuyk , H. J. Hendriks , Q. Jöbsis , E. Dompeling , Free Radic. Res. 2006, 40, 901. 17015269 10.1080/10715760500522648

[96] R. Stolarek , P. Bialasiewicz , M. Krol , D. Nowak , Clin. Chim. Acta 2010, 411, 1849. 20804745 10.1016/j.cca.2010.08.031

[97] W. Jung , M. Asaduddin , D. Yoo , D. Y. Lee , Y. Son , D. Kim , H. Keum , J. Lee , S.‐H. Park , S. Jon , Biomaterials 2024, 310, 122633. 38810387 10.1016/j.biomaterials.2024.122633

[98] A. M. Hall , G. J. Rhodes , R. M. Sandoval , P. R. Corridon , B. A. Molitoris , Kidney Int. 2013, 83, 72. 22992467 10.1038/ki.2012.328 PMC4136483

[99] M. Höckel , K. Schlenger , C. Knoop , P. Vaupel , Cancer Res. 1991, 51, 6098. 1933873

[100] M. Höckel , K. Schlenger , B. Aral , M. Mitze , U. Schaffer , P. Vaupel , Cancer Res. 1996, 56, 4509. 8813149

[101] C. M. Doll , M. Milosevic , M. Pintilie , R. P. Hill , A. W. Fyles , Int. J. Radiat. Oncol. Biol. Phys. 2003, 55, 1239. 12654433 10.1016/s0360-3016(02)04474-7

[102] R. K. Wong , A. Fyles , M. Milosevic , M. Pintilie , R. P. Hill , Int. J. Radiat. Oncol. Biol. Phys. 1997, 39, 405. 9308944 10.1016/s0360-3016(97)00328-3

[103] M. Höckel , C. Knoop , K. Schlenger , B. Vorndran , E. Baussmann , M. Mitze , P. G. Knapstein , P. Vaupel , Radiother. Oncol. 1993, 26, 45. 8438086 10.1016/0167-8140(93)90025-4

[104] B. S. Sørensen , M. R. Horsman , Front. Oncol. 2020, 10, 562. 32373534 10.3389/fonc.2020.00562 PMC7186437

[105] P. Vaupel , A. Mayer , S. Briest , M. Höckel , Cancer Res. 2003, 63, 7634. 14633681

[106] S. Runkel , A. Wischnik , J. Teubner , E. Kaven , J. Gaa , F. Melchert , Adv. Exp. Med. Biol. 1994, 345, 451. 8079743 10.1007/978-1-4615-2468-7_60

[107] M. Molls , P. Vaupel , C. Nieder , M. S. Anscher , The Impact of Tumor Biology on Cancer Treatment and Multidisciplinary Strategies, Springer Science & Business Media, Berlin, Heidelberg, 2009.

[108] E. L. Jones , L. R. Prosnitz , M. W. Dewhirst , P. K. Marcom , P. H. Hardenbergh , L. B. Marks , D. M. Brizel , Z. Vujaskovic , Clin. Cancer Res. 2004, 10, 4287. 15240513 10.1158/1078-0432.CCR-04-0133

[109] B. Movsas , J. D. Chapman , A. L. Hanlon , E. M. Horwitz , R. E. Greenberg , C. Stobbe , G. E. Hanks , A. Pollack , Urology 2002, 60, 634. 12385924 10.1016/s0090-4295(02)01858-7

[110] B. Movsas , J. D. Chapman , R. E. Greenberg , A. L. Hanlon , E. M. Horwitz , W. H. Pinover , C. Stobbe , G. E. Hanks , Cancer 2000, 89, 2018. 11064360 10.1002/1097-0142(20001101)89:9<2018::aid-cncr19>3.3.co;2-p

[111] T. Lin , X. Zhao , S. Zhao , H. Yu , W. Cao , W. Chen , H. Wei , H. Guo , Theranostics 2018, 8, 990. 29463995 10.7150/thno.22465 PMC5817106

[112] N. Lawrentschuk , A. M. Poon , S. S. Foo , L. G. Putra , C. Murone , I. D. Davis , D. M. Bolton , A. M. Scott , BJU Int 2005, 96, 540. 16104907 10.1111/j.1464-410X.2005.05681.x

[113] A. J. Brooks , J. Eastwood , I. J. Beckingham , K. J. Girling , Brit. J. Anaesth. 2004, 92, 735. 15033887 10.1093/bja/aeh112

[114] B. F. Jordan , P. Sonveaux , Front. Pharmacol. 2012, 3, 94. 22661950 10.3389/fphar.2012.00094 PMC3357106

[115] L. Albenberg , T. V. Esipova , C. P. Judge , K. Bittinger , J. Chen , A. Laughlin , S. Grunberg , R. N. Baldassano , J. D. Lewis , H. Li , S. R. Thom , F. D. Bushman , S. A. Vinogradov , G. D. Wu , Gastroenterology 2014, 147, 1055. 25046162 10.1053/j.gastro.2014.07.020 PMC4252572

[116] F. Rivera‐Chávez , L. F. Zhang , F. Faber , C. A. Lopez , M. X. Byndloss , E. E. Olsan , G. Xu , E. M. Velazquez , C. B. Lebrilla , S. E. Winter , A. J. Bäumler , Cell Host Microbe 2016, 19, 443. 27078066 10.1016/j.chom.2016.03.004 PMC4832419

[117] N. E. Zeitouni , S. Chotikatum , M. von Köckritz‐Blickwede , H. Y. Naim , Mol. Cell Pediatr. 2016, 3, 14. 27002817 10.1186/s40348-016-0041-y PMC4803720

[118] C. J. Kelly , L. Zheng , E. L. Campbell , B. Saeedi , C. C. Scholz , A. J. Bayless , K. E. Wilson , L. E. Glover , D. J. Kominsky , A. Magnuson , T. L. Weir , S. F. Ehrentraut , C. Pickel , K. A. Kuhn , J. M. Lanis , V. Nguyen , C. T. Taylor , S. P. Colgan , Cell Host Microbe 2015, 17, 662. 25865369 10.1016/j.chom.2015.03.005 PMC4433427

[119] K. O. Leonhardt , R. R. Landes , N. Engl. J. Med. 1963, 269, 115. 13929733 10.1056/NEJM196307182690301

[120] M. Grashei , P. Wodtke , J. G. Skinner , S. Sühnel , N. Setzer , T. Metzler , S. Gulde , M. Park , D. Witt , H. Mohr , C. Hundshammer , N. Strittmatter , N. S. Pellegata , K. Steiger , F. Schilling , Nat. Commun. 2023, 14, 5060. 37604826 10.1038/s41467-023-40747-3 PMC10442412

[121] F. A. Gallagher , M. I. Kettunen , S. E. Day , D.‐E. Hu , J. H. Ardenkjær‐Larsen , R. I. 'T Zandt , P. R. Jensen , M. Karlsson , K. Golman , M. H. Lerche , K. M. Brindle , Nature 2008, 453, 940. 18509335 10.1038/nature07017

[122] Z. M. Bhujwalla , D. Artemov , P. Ballesteros , S. Cerdan , R. J. Gillies , M. Solaiyappan , NMR Biomed. 2002, 15, 114. 11870907 10.1002/nbm.743

[123] N. Raghunand , X. He , R. van Sluis , B. Mahoney , B. Baggett , C. W. Taylor , G. Paine‐Murrieta , D. Roe , Z. M. Bhujwalla , R. J. Gillies , Br. J. Cancer 1999, 80, 1005. 10362108 10.1038/sj.bjc.6690455 PMC2363059

[124] N. Raghunand , M. I. Altbach , R. van Sluis , B. Baggett , C. W. Taylor , Z. M. Bhujwalla , R. J. Gillies , Biochem. Pharmacol. 1999, 57, 309. 9890558 10.1016/s0006-2952(98)00306-2

[125] A. S. Ojugo , P. M. McSheehy , D. J. McIntyre , C. McCoy , M. Stubbs , M. O. Leach , I. R. Judson , J. R. Griffiths , NMR Biomed. 1999, 12, 495. 10668042 10.1002/(sici)1099-1492(199912)12:8<495::aid-nbm594>3.0.co;2-k

[126] M. Stubbs , R. L. Veech , J. R. Griffiths , Adv. Enzyme Regul. 1995, 35, 101. 7572338 10.1016/0065-2571(94)00016-v

[127] S. P. Robinson , L. M. Rodrigues , J. R. Griffiths , M. Stubbs , Neoplasia 1999, 1, 537. 10935501 10.1038/sj.neo.7900027 PMC1508122

[128] R. J. Gillies , Z. Liu , Z. Bhujwalla , Am. J. Physiol. 1994, 267, C195. 8048479 10.1152/ajpcell.1994.267.1.C195

[129] S. Düwel , C. Hundshammer , M. Gersch , B. Feuerecker , K. Steiger , A. Buck , A. Walch , A. Haase , S. J. Glaser , M. Schwaiger , F. Schilling , Nat. Commun. 2017, 8, 15126. 28492229 10.1038/ncomms15126 PMC5482723

[130] S. Schreml , R. J. Meier , O. S. Wolfbeis , M. Landthaler , R.‐M. Szeimies , P. Babilas , Proc. Natl. Acad. Sci. USA 2011, 108, 2432. 21262842 10.1073/pnas.1006945108 PMC3038700

[131] B. Hollmann , M. Perkins , V. M. Chauhan , J. W. Aylott , K. R. Hardie , npj Biofilms Microbiomes 2021, 7, 50. 34140515 10.1038/s41522-021-00221-8 PMC8211749

[132] G. Hidalgo , A. Burns , E. Herz , A. G. Hay , P. L. Houston , U. Wiesner , L. W. Lion , Appl. Environ. Microbiol. 2009, 75, 7426. 19801466 10.1128/AEM.01220-09 PMC2786433

[133] S. Schreiber , T. H. Nguyen , M. Stüben , P. Scheid , Am. J. Physiol. Gastrointest. Liver Physiol. 2000, 279, G597. 10960360 10.1152/ajpgi.2000.279.3.G597

[134] R. Bücker , M. Azevedo‐Vethacke , C. Groll , D. Garten , C. Josenhans , S. Suerbaum , S. Schreiber , Sci. Rep. 2012, 2, 994. 23251780 10.1038/srep00994 PMC3524519

[135] M. Amiri , U. E. Seidler , K. Nikolovska , Front. Cell Dev. Biol. 2021, 9, 618135. 33553180 10.3389/fcell.2021.618135 PMC7862550

[136] A. N. Vaneev , P. V. Gorelkin , A. S. Garanina , H. V. Lopatukhina , S. S. Vodopyanov , A. V. Alova , O. O. Ryabaya , R. A. Akasov , Y. Zhang , P. Novak , S. V. Salikhov , M. A. Abakumov , Y. Takahashi , C. R. W. Edwards , N. L. Klyachko , A. G. Majouga , Y. E. Korchev , A. S. Erofeev , Anal. Chem. 2020, 92, 8010. 32441506 10.1021/acs.analchem.0c01256

[137] P. Niethammer , C. Grabher , A. T. Look , T. J. Mitchison , Nature 2009, 459, 996. 19494811 10.1038/nature08119 PMC2803098

[138] C. Nagaraja , B. L. Shashibhushan , Sagar, M. A. , P. H. Manjunath , Lung India 2012, 29, 123. 22628925 10.4103/0970-2113.95303 PMC3354484

[139] M. Corradi , P. Pignatti , G. Brunetti , M. Goldoni , A. Caglieri , S. Nava , G. Moscato , B. Balbi , Acta Biomed 2008, 79 Suppl 1, 73. 18924312

[140] A. Goraca , B. Skibska , J. Physiol. Pharmacol. 2008, 59, 379. 18622052

[141] V. de Broucker , S. M. Hassoun , S. Hulo , N. Chérot‐Kornobis , R. Nevière , R. Matran , A. Sobaszek , J.‐L. Edme , Vet. J. 2012, 194, 222. 22658821 10.1016/j.tvjl.2012.04.009

[142] S. D. Varma , P. S. Devamanoharan , Free Radic. Res. Commun. 1990, 8, 73. 2318421 10.3109/10715769009087976

[143] N. Kuge , M. Kohzuki , T. Sato , Free Radic. Res. 1999, 30, 119. 10193579 10.1080/10715769900300121

[144] P. S. Gill , C. S. Wilcox , Antioxid. Redox Signal. 2006, 8, 1597. 16987014 10.1089/ars.2006.8.1597

[145] L. Zhang , X. Wang , R. Cueto , C. Effi , Y. Zhang , H. Tan , X. Qin , Y. Ji , X. Yang , H. Wang , Redox Biol. 2019, 26, 101284. 31400697 10.1016/j.redox.2019.101284 PMC6831867

[146] Ł. Ożog , D. Aebisher , Eur. J. Clin. Exp. Med. 2018, 16, 123.

[147] D. R. Grimes , P. Kannan , D. R. Warren , B. Markelc , R. Bates , R. Muschel , M. Partridge , J. R. Soc. Interface 2016, 13, 20160070. 26935806 10.1098/rsif.2016.0070 PMC4843681

[148] P. Vaupel , A. B. Flood , H. M. Swartz , Appl. Magn. Reson. 2021, 52, 1451.

[149] C. D. McCaig , A. M. Rajnicek , B. Song , M. Zhao , Physiol. Rev. 2005, 85, 943. 15987799 10.1152/physrev.00020.2004

[150] R. Nuccitelli , Curr. Top. Dev. Biol. 2003, 58, 1. 14711011 10.1016/s0070-2153(03)58001-2

[151] B. Song , M. Zhao , J. V. Forrester , C. D. McCaig , Proc. Natl. Acad. Sci. USA 2002, 99, 13577. 12368473 10.1073/pnas.202235299 PMC129716

[152] A. G. Kléber , M. J. Janse , F. J. van Capelle , D. Durrer , Circ. Res. 1978, 42, 603. 639183 10.1161/01.res.42.5.603

[153] R. Coronel , F. J. Wilms‐Schopman , T. Opthof , F. J. van Capelle , M. J. Janse , Circ. Res. 1991, 68, 1241. 2018989 10.1161/01.res.68.5.1241

[154] M. E. Mycielska , M. B. A. Djamgoz , J. Cell Sci. 2004, 117, 1631. 15075225 10.1242/jcs.01125

[155] J. Cuzick , R. Holland , V. Barth , R. Davies , M. Faupel , I. Fentiman , H. Frischbier , J. LaMarque , M. Merson , V. Sacchini , D. Vanel , U. Veronesi , Lancet 1998, 352, 359. 9717923 10.1016/s0140-6736(97)10002-2

[156] P. N. Sawyer , E. Himmelfarb , I. Lustrin , H. Ziskind , Biophys. J. 1966, 6, 641. 5970567 10.1016/S0006-3495(66)86683-3 PMC1368020

[157] P. N. Sawyer , J. W. Pate , Am. J. Physiol. 1953, 175, 103. 13114360 10.1152/ajplegacy.1953.175.1.103

[158] Y. Liu , X. Zhang , C. Cao , Y. Zhang , J. Wei , Y. jun Li , W. Liang , Z. Hu , J. Zhang , Y. Wei , X. Deng , Adv. Funct. Mater. 2017, 27, 201703771.

[159] C. A. Erickson , R. Nuccitelli , J. Cell Biol. 1984, 98, 296. 6707093 10.1083/jcb.98.1.296 PMC2112998

[160] B. Lindemann , C. Voûte , in Frog Neurobiology, Springer, Berlin Heidelberg, 1976, pp. 169–210.

[161] C. D. McCaig , A. M. Rajnicek , B. Song , M. Zhao , Trends Neurosci. 2002, 25, 354. 12079763 10.1016/s0166-2236(02)02174-4

[162] F. Fröhlich , D. A. McCormick , Neuron 2010, 67, 129. 20624597 10.1016/j.neuron.2010.06.005 PMC3139922

[163] S. L. Schmidt , A. K. Iyengar , A. A. Foulser , M. R. Boyle , F. Fröhlich , Brain Stimul. 2014, 7, 878. 25129402 10.1016/j.brs.2014.07.033 PMC4259839

[164] S. Baillet , Nat. Neurosci. 2017, 20, 327. 28230841 10.1038/nn.4504

[165] Y. Grossman , K. Dzirasa , Neuropsychopharmacol. 2020, 45, 230. 10.1038/s41386-019-0511-8 PMC6879620 31506610

[166] M. Zhao , Semin. Cell Dev. Biol. 2009, 20, 674. 19146969 10.1016/j.semcdb.2008.12.009

[167] V. Yadav , J. D. Freedman , M. Grinstaff , A. Sen , Angew. Chem., Int. Ed. 2013, 52, 10997. 10.1002/anie.201305759 PMC4640360 24039057

[168] A. Boveris , E. Cadenas , B. Chance , Fed. Proc. 1981, 40, 195. 7461143

[169] E. Cadenas , A. Boveris , B. Chance , Biochem. J. 1980, 186, 659. 6249259 10.1042/bj1860659 PMC1161700

[170] J. Du , T. Deng , B. Cao , Z. Wang , M. Yang , J. Han , Front. Chem. 2023, 11, 1140128. 36874066 10.3389/fchem.2023.1140128 PMC9981976

[171] H. Schwabl , H. Klima , Complement. Med. Res. 2005, 12, 84. 10.1159/000083960 15947466

[172] A. M. Fernandes , K. Fero , W. Driever , H. A. Burgess , BioEssays 2013, 35, 775. 23712321 10.1002/bies.201300034 PMC4139915

[173] A. Ostadfar , Biofluid Mechanics: Principles and Applications, Academic Press, Cambridge, Massachusetts, 2016.

[174] S. J. Lunt , A. Fyles , R. P. Hill , M. Milosevic , Future Oncol. 2008, 4, 793. 19086846 10.2217/14796694.4.6.793

[175] H. T. Nia , L. L. Munn , R. K. Jain , Science 2020, 370, aaz0868. 10.1126/science.aaz0868 PMC8274378 33122355

[176] J. Riegler , Y. Labyed , S. Rosenzweig , V. Javinal , A. Castiglioni , C. X. Dominguez , J. E. Long , Q. Li , W. Sandoval , M. R. Junttila , S. J. Turley , J. Schartner , R. A. Carano , Clin. Cancer Res. 2018, 24, 4455. 29798909 10.1158/1078-0432.CCR-17-3262

[177] M. Plodinec , M. Loparic , C. A. Monnier , E. C. Obermann , R. Zanetti‐Dallenbach , P. Oertle , J. T. Hyotyla , U. Aebi , M. Bentires‐Alj , R. Y. Lim , C.‐A. Schoenenberger , Nat. Nanotechnol. 2012, 7, 757. 23085644 10.1038/nnano.2012.167

[178] S. K. Venkatesh , M. Yin , R. L. Ehman , Magn. Reson. Imaging 2013, 37, 544. 10.1002/jmri.23731 PMC3579218 23423795

[179] D. J. McGrail , Q. M. Kieu , M. R. Dawson , J. Cell Sci. 2014, 127, 2621. 24741068 10.1242/jcs.144378 PMC4058108

[180] H. Boudarel , J.‐D. Mathias , B. Blaysat , M. Grédiac , npj Biofilms Microbiomes 2018, 4, 17. 30131867 10.1038/s41522-018-0062-5 PMC6102240

[181] C. T. Kreis , R. M. A. Sullan , Nanoscale 2020, 12, 16819. 32760962 10.1039/d0nr03646c

[182] K. Kaiyrbekov , K. Endresen , K. Sullivan , Z. Zheng , Y. Chen , F. Serra , B. A. Camley , Proc. Natl. Acad. Sci. USA 2023, 120, 2301197120. 10.1073/pnas.2301197120 PMC10372565 37463218

[183] C. Leclech , D. Gonzalez‐Rodriguez , A. Villedieu , T. Lok , A.‐M. Déplanche , A. I. Barakat , Nat. Commun. 2022, 13, 2797. 35589751 10.1038/s41467-022-30488-0 PMC9120158

[184] K. G. Soans , A. P. Ramos , J. Sidhaye , A. Krishna , A. Solomatina , K. B. Hoffmann , R. Schlüßler , J. Guck , I. F. Sbalzarini , C. D. Modes , C. Norden , 2022, 32, 4817. 10.1016/j.cub.2022.09.034 36208624

[185] E. Lång , A. Lång , P. Blicher , T. Rognes , P. G. Dommersnes , S. O. Bøe , Sci. Adv. 2024, 10, adk4825. 10.1126/sciadv.adk4825 PMC11023523 38630812

[186] I. Pajic‐Lijakovic , M. Milivojevic , A. G. Clark , Front. Cell Dev. Biol. 2022, 10, 901026. 35859899 10.3389/fcell.2022.901026 PMC9289519

[187] J. Park , D.‐H. Kim , H.‐N. Kim , C. J. Wang , M. K. Kwak , E. Hur , K.‐Y. Suh , S. S. An , A. Levchenko , Nat. Mater. 2016, 15, 792. 26974411 10.1038/nmat4586 PMC5517090

[188] K. R. Levental , H. Yu , L. Kass , J. N. Lakins , M. Egeblad , J. T. Erler , S. F. T. Fong , K. Csiszar , A. Giaccia , W. Weninger , M. Yamauchi , D. L. Gasser , V. M. Weaver , Cell 2009, 139, 891. 19931152 10.1016/j.cell.2009.10.027 PMC2788004

[189] G. Führ , E. Richter , H. Zimmermann , H. Hitzler , H. Niehus , R. Hagedorn , Biol. Chem. 1998, 379, 1161. 9792450 10.1515/bchm.1998.379.8-9.1161

[190] A. Heindl , S. Nawaz , Y. Yuan , Lab. Invest. 2015, 95, 377. 25599534 10.1038/labinvest.2014.155

[191] T. Cui , J. Yu , C. Wang , S. Chen , Q. Li , K. Guo , R. Qing , G. Wang , J. Ren , Adv. Sci. 2022, 9, 2201254. 10.1002/advs.202201254 PMC9353480 35596608

[192] Q. Jia , A. Wang , Y. Yuan , B. Zhu , H. Long , Exp. Hematol. Oncol. 2022, 11, 24. 35461288 10.1186/s40164-022-00277-y PMC9034473

[193] R. Ge , Z. Wang , L. Cheng , npj Precis. Onc 2022, 6, 1. 10.1038/s41698-022-00272-w PMC9068628 35508696

[194] P. S. Stewart , M. J. Franklin , Nat. Rev. Microbiol. 2008, 6, 199. 18264116 10.1038/nrmicro1838

[195] J. Jo , A. Price‐Whelan , L. E. Dietrich , Nat. Rev. Microbiol. 2022, 20, 593. 35149841 10.1038/s41579-022-00692-2 PMC9590228

[196] M. Proudfoot , M. W. Woolrich , A. C. Nobre , M. R. Turner , Pract. Neurol. 2014, 14, 336. 24647614 10.1136/practneurol-2013-000768 PMC4174130

[197] E. P. Wijk , R. V. Wijk , Complement. Med. Res. 2005, 12, 96. 10.1159/000083935 15947468

[198] A. Shanei , Z. Alinasab , A. Kiani , M. A. Nematollahi , J. Biomed. Phys. Eng. 2017, 7, 389. 29445715 PMC5809932

[199] T. Amano , M. Kobayashi , B. Devaraj , M. Usa , H. Inaba , Urol. Res. 1995, 23, 315. 8839388 10.1007/BF00300020

[200] J. Kim , C. Choi , J. Lim , H. You , S.‐B. Sim , Y.‐K. Yom , E.‐H. Kim , K.‐S. Soh , J. Altern. Complement. Med. 2005, 11, 879. 16296922 10.1089/acm.2005.11.879

[201] E. V. Mukerjee , R. R. Isseroff , R. Nuccitelli , S. D. Collins , R. L. Smith , Conf. Proc. IEEE Eng. Med. Biol. Soc. 2006, 2006, 4326. 17947077 10.1109/IEMBS.2006.260205

[202] R. Nuccitelli , P. Nuccitelli , S. Ramlatchan , R. Sanger , P. J. S. Smith , Wound Repair Regen 2008, 16, 432. 18471262 10.1111/j.1524-475X.2008.00389.x PMC3086402

[203] R. B. Borgens , Science 1984, 225, 478. 6740320 10.1126/science.6740320

[204] Y. Boucher , L. T. Baxter , R. K. Jain , Cancer Res. 1990, 50, 4478. 2369726

[205] S. D. Nathanson , L. Nelson , Ann. Surg. Oncol. 1994, 1, 333. 7850532 10.1007/BF03187139

[206] B. D. Curti , W. J. Urba , W. G. Alvord , J. E. Janik , J. W. Smith , K. Madara , D. L. Longo , Cancer Res. 1993, 53, 2204. 8485703

[207] J. R. Less , M. C. Posner , Y. Boucher , D. Borochovitz , N. Wolmark , R. K. Jain , Cancer Res. 1992, 52, 6371. 1423283

[208] M. Milosevic , A. Fyles , D. Hedley , M. Pintilie , W. Levin , L. Manchul , R. Hill , Cancer Res. 2001, 61, 6400. 11522633

[209] C. G. Willett , Y. Boucher , E. di Tomaso , D. G. Duda , L. L. Munn , R. T. Tong , D. C. Chung , D. V. Sahani , S. P. Kalva , S. V. Kozin , M. Mino , K. S. Cohen , D. T. Scadden , A. C. Hartford , A. J. Fischman , J. W. Clark , D. P. Ryan , A. X. Zhu , L. S. Blaszkowsky , H. X. Chen , P. C. Shellito , G. Y. Lauwers , R. K. Jain , Nat. Med. 2004, 10, 145. 14745444 10.1038/nm988 PMC2693485

[210] T. Lund , H. Wiig , R. K. Reed , Am. J. Physiol. 1988, 255, H1069. 3189570 10.1152/ajpheart.1988.255.5.H1069

[211] S. R. Chary , R. K. Jain , Proc. Natl. Acad. Sci. USA 1989, 86, 5385. 2748592 10.1073/pnas.86.14.5385 PMC297627

[212] C. Blatter , E. F. J. Meijer , A. S. Nam , D. Jones , B. E. Bouma , T. P. Padera , B. J. Vakoc , Sci. Rep. 2016, 6, 29035. 27377852 10.1038/srep29035 PMC4932526

[213] P. C. W. Fung , R. K. C. Kong , Open J. Mol. Integr. Physiol. 2016, 06, 45.

[214] H. Zimmermann , R. Hagedorn , E. Richter , G. Fuhr , Eur. Biophys. J. 1999, 28, 516. 10460345 10.1007/s002490050234

[215] A. Evans , P. Whelehan , K. Thomson , K. Brauer , L. Jordan , C. Purdie , D. McLean , L. Baker , S. Vinnicombe , A. Thompson , Br. J. Cancer 2012, 107, 224. 22691969 10.1038/bjc.2012.253 PMC3394981

[216] D. L. Cochlin , R. H. Ganatra , D. F. R. Griffiths , Clin. Radiol. 2002, 57, 1014. 12409113 10.1053/crad.2002.0989

[217] S. Carrara , M. D. Leo , F. Grizzi , L. Correale , D. Rahal , A. Anderloni , F. Auriemma , A. Fugazza , P. Preatoni , R. Maselli , C. Hassan , E. Finati , B. Mangiavillano , A. Repici , Gastrointest. Endosc. 2018, 87, 1464. 29329992 10.1016/j.gie.2017.12.031

[218] O. Rouvière , C. Melodelima , A. Hoang Dinh , F. Bratan , G. Pagnoux , T. Sanzalone , S. Crouzet , M. Colombel , F. Mège‐Lechevallier , R. Souchon , Eur. Radiol. 2017, 27, 1858. 27553936 10.1007/s00330-016-4534-9

[219] M. F. Ullo , L. B. Case , WIREs Mech. Dis. 2023, 15, 1604. 10.1002/wsbm.1604 36781396

[220] D. Y. Vogel , P. D. Heijnen , M. Breur , H. E. De Vries , A. T. Tool , S. Amor , C. D. Dijkstra , J. Neuroinflamm. 2014, 11, 23. 10.1186/1742-2094-11-23 PMC3937114 24485070

[221] R. Mo , W. Ma , W. Zhou , B. Gao , PLoS Pathog. 2022, 18, 1010953. 10.1371/journal.ppat.1010953 PMC9665402 36327346

[222] R. Karmakar , J. Basic Microbiol. 2021, 61, 366. 33687766 10.1002/jobm.202000661

[223] C. DiPetrillo , E. Smith , in Methods in Cell Biology, Elsevier, Amsterdam 2009, pp. 163–180. 10.1016/S0091-679X(08)92011-2 20409805

[224] X. Ji , X. Tian , S. Feng , L. Zhang , J. Wang , R. Guo , Y. Zhu , X. Yu , Y. Zhang , H. Du , V. Zablotskii , X. Zhang , Research 2023, 6, 0080. 36939445 10.34133/research.0080 PMC10017101

[225] S. Maxson , E. A. Lopez , D. Yoo , A. Danilkovitch‐Miagkova , M. A. LeRoux , Stem Cells Transl. Med. 2012, 1, 142. 23197761 10.5966/sctm.2011-0018 PMC3659685

[226] X. Yang , C. Lin , X. Chen , S. Li , X. Li , B. Xiao , Nature 2022, 604, 377. 35388220 10.1038/s41586-022-04574-8

[227] P. Nordenfelt , T. I. Moore , S. B. Mehta , J. M. Kalappurakkal , V. Swaminathan , N. Koga , T. J. Lambert , D. Baker , J. C. Waters , R. Oldenbourg , T. Tani , S. Mayor , C. M. Waterman , T. A. Springer , Nat. Commun. 2017, 8, 2047. 29229906 10.1038/s41467-017-01848-y PMC5725580

[228] S. Mochiji , K. Wakabayashi , Commun. Integr. Biol. 2012, 5, 196. 22808332 10.4161/cib.18890 PMC3376063

[229] M. Böhm , G. Kreimer , in Progress in Botany (Eds: F. M. Cánovas , U. Lüttge , M.‐C. Risueño , H. Pretzsch ), Springer International Publishing, Berlin, 2020, *Vol*. 82 pp. 259‐304.

[230] J. Su , Y. Song , Z. Zhu , X. Huang , J. Fan , J. Qiao , F. Mao , Signal Transduct. Target. Ther. 2024, 9, 196. 39107318 10.1038/s41392-024-01888-z PMC11382761

[231] Y. Bai , C. He , P. Chu , J. Long , X. Li , X. Fu , eLife 2021, 10, 67316. 10.7554/eLife.67316 PMC8563000 34726151

[232] S. SenGupta , C. A. Parent , J. E. Bear , Nat. Rev. Mol. Cell Biol. 2021, 22, 529. 33990789 10.1038/s41580-021-00366-6 PMC8663916

[233] P. Friedl , K. Wolf , J. Cell Biol. 2010, 188, 11. 19951899 10.1083/jcb.200909003 PMC2812848

[234] D. B. Kearns , Nat. Rev. Microbiol. 2010, 8, 634. 20694026 10.1038/nrmicro2405 PMC3135019

[235] M. Tian , C. Zhang , R. Zhang , J. Yuan , Biophys. J. 2021, 120, 1615. 33636168 10.1016/j.bpj.2021.02.021 PMC8204213

[236] R. Sunyer , V. Conte , J. Escribano , A. Elosegui‐Artola , A. Labernadie , L. Valon , D. Navajas , J. M. García‐Aznar , J. J. Muñoz , P. Roca‐Cusachs , X. Trepat , Science 2016, 353, 1157. 27609894 10.1126/science.aaf7119

[237] E. Theveneau , L. Marchant , S. Kuriyama , M. Gull , B. Moepps , M. Parsons , R. Mayor , Dev. Cell 2010, 19, 39. 20643349 10.1016/j.devcel.2010.06.012 PMC2913244

[238] G. Malet‐Engra , W. Yu , A. Oldani , J. Rey‐Barroso , N. S. Gov , G. Scita , L. Dupré , Curr. Biol. 2015, 25, 242. 25578904 10.1016/j.cub.2014.11.030

[239] X. Serra‐Picamal , V. Conte , R. Vincent , E. Anon , D. T. Tambe , E. Bazellieres , J. P. Butler , J. J. Fredberg , X. Trepat , Nat. Phys. 2012, 8, 628.

[240] N. Ueki , T. Ide , S. Mochiji , Y. Kobayashi , R. Tokutsu , N. Ohnishi , K. Yamaguchi , S. Shigenobu , K. Tanaka , J. Minagawa , T. Hisabori , M. Hirono , K. Wakabayashi , Proc. Natl. Acad. Sci. USA 2016, 113, 5299. 27122315 10.1073/pnas.1525538113 PMC4868408

[241] D. A. Bazylinski , R. B. Frankel , Nat. Rev. Microbiol. 2004, 2, 217. 15083157 10.1038/nrmicro842

[242] S. Coppola , V. Kantsler , Sci. Rep. 2021, 11, 399. 33432106 10.1038/s41598-020-79887-7 PMC7801662

[243] K. Xiong , L. Xu , J. Lin , F. Mou , J. Guan , Research 2020, 2020, 6213981. 32832907 10.34133/2020/6213981 PMC7424550

[244] Y. Tu , F. Peng , X. Sui , Y. Men , P. B. White , J. C. M. Van Hest , D. A. Wilson , Nat. Chem. 2017, 9, 480. 28430193 10.1038/nchem.2674

[245] Y. Ji , X. Lin , H. Zhang , Y. Wu , J. Li , Q. He , Angew. Chem., Int. Ed. 2019, 58, 4184. 10.1002/anie.201812860 30701642

[246] Y. Lee , J. Kim , U. Bozuyuk , N. O. Dogan , M. T. A. Khan , A. Shiva , A. Wild , M. Sitti , Adv. Mater. 2023, 35, 2209812. 10.1002/adma.202209812 36585849

[247] Y. Pan , X. Ma , Y. Wu , Z. Zhao , Q. He , Y. Ji , Colloid Surface A 2024, 684, 133070.

[248] M. A. Stuart , W. T. Huck , J. Genzer , M. Müller , C. Ober , M. Stamm , G. B. Sukhorukov , I. Szleifer , V. V. Tsukruk , M. Urban , F. Winnik , S. Zauscher , I. Luzinov , S. Minko , Nat. Mater. 2010, 9, 101. 20094081 10.1038/nmat2614

[249] C. D. Alarcón , S. Pennadam , C. Alexander , Chem. Soc. Rev. 2005, 34, 276. 15726163 10.1039/b406727d

[250] P. Bawa , V. Pillay , Y. E. Choonara , L. C. Du Toit , Biomed. Mater. 2009, 4, 022001. 19261988 10.1088/1748-6041/4/2/022001

[251] E. Cabane , X. Zhang , K. Langowska , C. G. Palivan , W. Meier , Biointerphases 2012, 7, 9. 22589052 10.1007/s13758-011-0009-3

[252] N. R. B. Boase , E. R. Gillies , R. Goh , R. E. Kieltyka , J. B. Matson , F. Meng , A. Sanyal , O. Sedláček , Biomacromolecules 2024, 25, 5417. 39197109 10.1021/acs.biomac.4c00690

[253] M. You , C. Chen , L. Xu , F. Mou , J. Guan , Acc. Chem. Res. 2018, 51, 3006. 30444357 10.1021/acs.accounts.8b00291

[254] Y. Hong , N. M. K. Blackman , N. D. Kopp , A. Sen , D. Velegol , Phys. Rev. Lett. 2007, 99, 178103. 17995374 10.1103/PhysRevLett.99.178103

[255] Z. Xiao , A. Nsamela , B. Garlan , J. Simmchen , Angew. Chem., Int. Ed. 2022, 61, 202117768. 10.1002/anie.202117768 PMC9401050 35156269

[256] J. Wang , B. J. Toebes , A. S. Plachokova , Q. Liu , D. Deng , J. A. Jansen , F. Yang , D. A. Wilson , Adv. Healthcare Mater. 2020, 9, 1901710. 10.1002/adhm.201901710 32142216

[257] S. Sanchez , A. A. Solovev , Y. Mei , O. G. Schmidt , J. Am. Chem. Soc. 2010, 132, 13144. 20860367 10.1021/ja104362r

[258] H. Zhang , Z. Cao , Q. Zhang , J. Xu , S. L. Yun , K. Liang , Z. Gu , Small 2020, 16, 2002732. 10.1002/smll.202002732 33048446

[259] A. Y. Jee , Y. K. Cho , S. Granick , T. Tlusty , Proc. Natl. Acad. Sci. USA 2018, 115, 10812. 10.1073/pnas.1814180115 PMC6243271 30385635

[260] A. Y. Jee , S. Dutta , Y. K. Cho , T. Tlusty , S. Granick , Proc. Natl. Acad. Sci. USA 2018, 115, 14. 29255047 10.1073/pnas.1717844115 PMC5776828

[261] Z. Wu , M. Zhou , X. Tang , J. Zeng , Y. Li , Y. Sun , J. Huang , L. Chen , M. Wan , C. Mao , ACS Nano 2022, 16, 3808. 35199998 10.1021/acsnano.1c08391

[262] A. Joseph , C. Contini , D. Cecchin , S. Nyberg , L. Ruiz‐Perez , J. Gaitzsch , G. Fullstone , X. Tian , J. Azizi , J. Preston , G. Volpe , G. Battaglia , Sci. Adv. 2017, 3, 1700362. 10.1126/sciadv.1700362 PMC5540238 28782037

[263] Z. Liu , T. Li , N. Li , Y. Wang , L. Chen , X. Tang , M. Wan , C. Mao , Sci. China Chem. 2022, 65, 989.

[264] Y. Li , J. Liu , Y. Wu , Q. He , *J. Am. Chem. Soc*. 2024, jacs.4c00334.

[265] F. Mou , Q. Xie , J. Liu , S. Che , L. Bahmane , M. You , J. Guan , Natl. Sci. Rev. 2021, 8, nwab066. 34876993 10.1093/nsr/nwab066 PMC8645024

[266] M. N. Popescu , W. E. Uspal , C. Bechinger , P. Fischer , Nano Lett. 2018, 18, 5345. 30047271 10.1021/acs.nanolett.8b02572

[267] C. Zhou , C. Gao , Y. Wu , T. Si , M. Yang , Q. He , Angew. Chem., Int. Ed. 2022, 61, 202116013. 10.1002/anie.202116013 34981604

[268] A. Somasundar , S. Ghosh , F. Mohajerani , L. N. Massenburg , T. Yang , P. S. Cremer , D. Velegol , A. Sen , Nat. Nanotechnol. 2019, 14, 1129. 31740796 10.1038/s41565-019-0578-8

[269] N. S. Mandal , A. Sen , R. D. Astumian , J. Am. Chem. Soc. 2023, 145, 5730. 36867055 10.1021/jacs.2c11945

[270] J. Agudo‐Canalejo , P. Illien , R. Golestanian , Nano Lett. 2018, 18, 2711. 29552886 10.1021/acs.nanolett.8b00717

[271] P. Illien , T. Adeleke‐Larodo , R. Golestanian , Epl‐europhys. Lett 2017, 119, 40002.

[272] P. Illien , X. Zhao , K. K. Dey , P. J. Butler , A. Sen , R. Golestanian , Nano Lett. 2017, 17, 4415. 28593755 10.1021/acs.nanolett.7b01502

[273] F. Zhang , Z. Li , C. Chen , H. Luan , R. H. Fang , L. Zhang , J. Wang , Adv. Mater. 2024, 36, 2303714. 10.1002/adma.202303714 PMC10799182 37471001

[274] F. Zhang , Z. Guo , Z. Li , H. Luan , Y. Yu , A. T. Zhu , S. Ding , W. Gao , R. H. Fang , L. Zhang , J. Wang , Sci. Adv. 2024, 10, adn6157. 10.1126/sciadv.adn6157 PMC11168470 38865468

[275] D. Park , S. J. Park , S. Cho , Y. Lee , Y. K. Lee , J.‐J. Min , B. J. Park , S. Y. Ko , J.‐O. Park , S. Park , Biotechnol. Bioeng. 2014, 111, 134. 23893511 10.1002/bit.25007

[276] A. Sahari , M. A. Traore , B. E. Scharf , B. Behkam , Biomed. Microdevices 2014, 16, 717. 24907051 10.1007/s10544-014-9876-y

[277] J. H. Zheng , V. H. Nguyen , S.‐N. Jiang , S.‐H. Park , W. Tan , S. H. Hong , M. G. Shin , I.‐J. Chung , Y. Hong , H.‐S. Bom , H. E. Choy , S. E. Lee , J. H. Rhee , J.‐J. Min , Sci. Transl. Med. 2017, 9, aak9537. 10.1126/scitranslmed.aak9537 28179508

[278] H. Zhang , Z. Li , C. Gao , X. Fan , Y. Pang , T. Li , Z. Wu , H. Xie , Q. He , Sci. Rob. 2021, 6, aaz9519. 10.1126/scirobotics.aaz9519 34043546

[279] P. Sun , Q. Deng , L. Kang , Y. Sun , J. Ren , X. Qu , ACS Nano 2020, 14, 13894. 32955858 10.1021/acsnano.0c06290

[280] J. Han , J. Zhen , V. Du Nguyen , G. Go , Y. Choi , S. Y. Ko , J.‐O. Park , S. Park , Sci. Rep. 2016, 6, 28717. 27346486 10.1038/srep28717 PMC4921872

[281] V. D. Nguyen , H.‐K. Min , H. Y. Kim , J. Han , Y. H. Choi , C.‐S. Kim , J.‐O. Park , E. Choi , ACS Nano 2021, 15, 8492. 33973786 10.1021/acsnano.1c00114

[282] C. Chen , X. Chang , P. Angsantikul , J. Li , B. Esteban‐Fernández de Ávila , E. Karshalev , W. Liu , F. Mou , S. He , R. Castillo , Y. Liang , J. Guan , L. Zhang , J. Wang , Adv. Biosyst. 2018, 2, 1700160.

[283] H. Xu , M. Medina‐Sanchez , V. Magdanz , L. Schwarz , F. Hebenstreit , O. G. Schmidt , ACS Nano 2018, 12, 327. 29202221 10.1021/acsnano.7b06398

[284] B. W. Park , S. H. Jung , S. Das , S. M. Lee , J.‐H. Park , H. Kim , J.‐W. Hwang , S. Lee , H.‐J. Kim , H.‐Y. Kim , S. Jung , D.‐W. Cho , J. Jang , K. Ban , H.‐J. Park , Sci. Adv. 2020, 6, aay6994. 10.1126/sciadv.aay6994 PMC7141892 32284967

[285] K. S. Aboody , A. Brown , N. G. Rainov , K. A. Bower , S. Liu , W. Yang , J. E. Small , U. Herrlinger , V. Ourednik , P. M. Black , X. O. Breakefield , E. Y. Snyder , Proc. Natl. Acad. Sci. USA 2000, 97, 12846. 11070094 10.1073/pnas.97.23.12846 PMC18852

[286] S. Li , L. Wang , Y. Gu , L. Lin , M. Zhang , M. Jin , C. Mao , J. Zhou , W. Zhang , X. Huang , C. Corbo , W. Tao , E. Lu , J. Liu , Matter 2021, 4, 3621.

[287] C. Wang , W. Sun , Y. Ye , Q. Hu , H. N. Bomba , Z. Gu , Nat. Biomed. Eng. 2017, 1, 1.

[288] P. Tiet , J. Berlin , Biochem. Pharmacol. 2017, 145, 18. 28941937 10.1016/j.bcp.2017.09.006 PMC5681359

[289] Z. Li , Y. Duan , F. Zhang , H. Luan , W.‐T. Shen , Y. Yu , N. Xian , Z. Guo , E. Zhang , L. Yin , R. H. Fang , W. Gao , L. Zhang , J. Wang , Sci. Rob. 2024, 9, adl2007. 10.1126/scirobotics.adl2007 38924422

[290] R. Luo , J. Liu , Q. Cheng , M. Shionoya , C. Gao , R. Wang , Sci. Adv. 2024, 10, ado6798. 10.1126/sciadv.ado6798 PMC11212727 38941458

[291] T. Yin , Q. Fan , F. Hu , X. Ma , Y. Yin , B. Wang , L. Kuang , X. Hu , B. Xu , Y. Wang , Nano Lett. 2022, 22, 6606. 35948420 10.1021/acs.nanolett.2c01863

[292] S. Tang , D. Tang , H. Zhou , Y. Li , D. Zhou , X. Peng , C. Ren , Y. Su , S. Zhang , H. Zheng , F. Wan , J. Yoo , H. Han , X. Ma , W. Gao , S. Wu , Proc. Natl. Acad. Sci. USA 2024, 121, 2403460121. 10.1073/pnas.2403460121 PMC11287275 39008666

[293] C. M. Hu , R. H. Fang , K. C. Wang , B. T. Luk , S. Thamphiwatana , D. Dehaini , P. Nguyen , P. Angsantikul , C. H. Wen , A. V. Kroll , C. Carpenter , M. Ramesh , V. Qu , S. H. Patel , J. Zhu , W. Shi , F. M. Hofman , T. C. Chen , W. Gao , K. Zhang , S. Chien , L. Zhang , Nature 2015, 526, 118. 26374997 10.1038/nature15373 PMC4871317

[294] B. Bahmani , H. Gong , B. T. Luk , K. J. Haushalter , E. DeTeresa , M. Previti , J. Zhou , W. Gao , J. D. Bui , L. Zhang , R. H. Fang , J. Zhang , Nat. Commun. 2021, 12, 1999. 33790276 10.1038/s41467-021-22311-z PMC8012593

[295] Y. Kim , X. Zhao , Chem. Rev. 2022, 122, 5317. 35104403 10.1021/acs.chemrev.1c00481 PMC9211764

[296] U. Bozuyuk , E. Suadiye , A. Aghakhani , N. O. Dogan , J. Lazovic , M. E. Tiryaki , M. Schneider , A. C. Karacakol , S. O. Demir , G. Richter , M. Sitti , Adv. Funct. Mater. 2022, 32, 2109741.

[297] S. Das , A. Garg , A. I. Campbell , J. Howse , A. Sen , D. Velegol , R. Golestanian , S. J. Ebbens , Nat. Commun. 2015, 6, 8999. 26627125 10.1038/ncomms9999 PMC4686856

[298] J. Simmchen , J. Katuri , W. E. Uspal , M. N. Popescu , M. Tasinkevych , S. Sánchez , Nat. Commun. 2016, 7, 10598. 26856370 10.1038/ncomms10598 PMC4748132

[299] C. Maggi , J. Simmchen , F. Saglimbeni , J. Katuri , M. Dipalo , F. De Angelis , S. Sanchez , R. Di Leonardo , Small 2016, 12, 446. 26649462 10.1002/smll.201502391

[300] J. Katuri , D. Caballero , R. Voituriez , J. Samitier , S. Sanchez , ACS Nano 2018, 12, 7282. 29949338 10.1021/acsnano.8b03494

[301] L. S. Palacios , S. Tchoumakov , M. Guix , I. Pagonabarraga , S. Sánchez , A. G. Grushin , Nat. Commun. 2021, 12, 4691. 34344869 10.1038/s41467-021-24948-2 PMC8333048

[302] J. Palacci , S. Sacanna , A. Abramian , J. Barral , K. Hanson , A. Y. Grosberg , D. J. Pine , P. M. Chaikin , Sci. Adv. 2015, 1, 1400214. 10.1126/sciadv.1400214 PMC4640647 26601175

[303] R. Baker , J. E. Kauffman , A. Laskar , O. E. Shklyaev , M. Potomkin , L. Dominguez‐Rubio , H. Shum , Y. Cruz‐Rivera , I. S. Aranson , A. C. Balazs , A. Sen , Nanoscale 2019, 11, 10944. 31139774 10.1039/c8nr10257k

[304] R. Dey , C. M. Buness , B. V. Hokmabad , C. Jin , C. C. Maass , Nat. Commun. 2022, 13, 2952. 35618708 10.1038/s41467-022-30611-1 PMC9135748

[305] L. Ren , D. Zhou , Z. Mao , P. Xu , T. J. Huang , T. E. Mallouk , ACS Nano 2017, 11, 10591. 28902492 10.1021/acsnano.7b06107

[306] J. Katuri , W. E. Uspal , J. Simmchen , A. Miguel‐López , S. Sánchez , Sci. Adv. 2018, 4, aao1755. 10.1126/sciadv.aao1755 PMC5787385 29387790

[307] J. Yu , L. Yang , L. Zhang , Int. J. Robot. Res. 2018, 37, 912.

[308] Q. Wang , L. Yang , B. Wang , E. Yu , J. Yu , L. Zhang , ACS Appl. Mater. Interfaces 2019, 11, 1630. 30560650 10.1021/acsami.8b17402

[309] C. M. Wentworth , A. C. Castonguay , P. G. Moerman , C. H. Meredith , R. V. Balaj , S. I. Cheon , L. D. Zarzar , Angew. Chem., Int. Ed. 2022, 61, 202204510. 10.1002/anie.202204510 35678216

[310] S. Chen , X. Peetroons , A. C. Bakenecker , F. Lezcano , I. S. Aranson , S. Sánchez , Nat. Commun. 2024, 15, 9315. 39472587 10.1038/s41467-024-53664-w PMC11522643

[311] J. Zhang , J. Guo , F. Mou , J. Guan , Micromachines 2018, 9, 88.30393364

[312] R. Golestanian , Phys. Rev. Lett. 2012, 108, 038303. 22400792 10.1103/PhysRevLett.108.038303

[313] J. Zhang , R. Alert , J. Yan , N. S. Wingreen , S. Granick , Nat. Phys. 2021, 17, 961.

[314] E. Lauga , The Fluid Dynamics of Cell Motility, Cambridge University Press, Cambridge 2020.

[315] F. Rojas‐Pérez , B. Delmotte , S. Michelin , J. Fluid Mech. 2021, 919, A22.

[316] Y. Peng , P. Xu , S. Duan , J. Liu , J. L. Moran , W. Wang , Angew. Chem., Int. Ed. 2022, 61, 202116041. 10.1002/anie.202116041 34994039

[317] F. Mou , J. Zhang , Z. Wu , S. Du , Z. Zhang , L. Xu , J. Guan , iScience 2019, 19, 415. 31421596 10.1016/j.isci.2019.07.050 PMC6704395

[318] J. Zheng , J. Chen , Y. Jin , Y. Wen , Y. Mu , C. Wu , Y. Wang , P. Tong , Z. Li , X. Hou , J. Tang , Nature 2023, 617, 499. 37198311 10.1038/s41586-023-05873-4 PMC10191859

[319] D. Cao , Z. Yan , D. Cui , M. Y. Khan , S. Duan , G. Xie , Z. He , D. Y. Xing , W. Wang , Langmuir 2024, 40, 10884. 38756056 10.1021/acs.langmuir.4c00058

[320] C. Wu , J. Dai , X. Li , L. Gao , J. Wang , J. Liu , J. Zheng , X. Zhan , J. Chen , X. Cheng , M. Yang , J. Tang , Nat. Nanotechnol. 2021, 16, 288. 33432205 10.1038/s41565-020-00825-9

[321] X. Chen , Y. Xu , C. Zhou , K. Lou , Y. Peng , H. P. Zhang , W. Wang , Sci. Adv. 2022, 8, abn9130. 10.1126/sciadv.abn9130 PMC9132452 35613263

[322] Y. C. Tseng , J. Song , J. Zhang , E. Shandilya , A. Sen , J. Am. Chem. Soc. 2024, 146, 16097. 38805671 10.1021/jacs.4c03415

[323] W. Wang , W. Duan , A. Sen , T. E. Mallouk , Proc. Natl. Acad. Sci. USA 2013, 110, 17744. 24127603 10.1073/pnas.1311543110 PMC3816472

[324] M. He , J. P. Gales , É. Ducrot , Z. Gong , G.‐R. Yi , S. Sacanna , D. J. Pine , Nature 2020, 585, 524. 32968261 10.1038/s41586-020-2718-6

[325] Y. Wang , Y. Wang , D. R. Breed , V. N. Manoharan , L. Feng , A. D. Hollingsworth , M. Weck , D. J. Pine , Nature 2012, 491, 51. 23128225 10.1038/nature11564

[326] M. R. Jones , N. C. Seeman , C. A. Mirkin , Science 2015, 347, 1260901. 25700524 10.1126/science.1260901

[327] T. Bauerle , R. C. Loffler , C. Bechinger , Nat. Commun. 2020, 11, 2547. 32439919 10.1038/s41467-020-16161-4 PMC7242396

[328] Y. C. Tseng , J. Song , J. Zhang , E. Shandilya , A. Sen , *J. Am. Chem. Soc*. 2024, jacs.4c03415. 10.1021/jacs.4c03415 38805671

[329] B. Dai , J. Wang , Z. Xiong , X. Zhan , W. Dai , C.‐C. Li , S.‐P. Feng , J. Tang , Nat. Nanotechnol. 2016, 11, 1087. 27749832 10.1038/nnano.2016.187

[330] C. Lozano , B. ten Hagen , H. Löwen , C. Bechinger , Nat. Commun. 2016, 7, 12828. 27687580 10.1038/ncomms12828 PMC5056439

[331] C. Zhou , L. Yang , Y. Wu , M. Yang , Q. He , Chem.‐Eur. J. 2022, 28, 202202319.

[332] X. Xia , Y. Li , X. Xiao , Z. Zhang , C. Mao , T. Li , M. Wan , Small 2024, 20, 2306191. 10.1002/smll.202306191 37775935

[333] B. Liebchen , H. Löwen , Acc. Chem. Res. 2018, 51, 2982. 30375857 10.1021/acs.accounts.8b00215

[334] Z. T. Liu , Y. Shi , Y. Zhao , H. Chaté , X. Shi , T. H. Zhang , Proc. Natl. Acad. Sci. USA 2021, 118, 2104724118. 10.1073/pnas.2104724118 PMC8501844 34588304

[335] J. Palacci , S. Sacanna , A. P. Steinberg , D. J. Pine , P. M. Chaikin , Science 2013, 339, 936. 23371555 10.1126/science.1230020

[336] Z. Yang , L. Wang , Z. Gao , X. Hao , M. Luo , Z. Yu , J. Guan , ACS Nano 2023, 17, 6023. 36892585 10.1021/acsnano.3c00548

[337] T. Gao , J. Lin , L. Xu , J. Guan , Nanomaterials 2022, 12, 2049. 35745388 10.3390/nano12122049 PMC9229371

[338] Y. Ji , X. Lin , Z. Wu , Y. Wu , W. Gao , Q. He , Angew. Chem., Int. Ed. 2019, 58, 12200. 10.1002/anie.201907733 31282598

[339] T. Li , Z. Liu , J. Hu , L. Chen , T. Chen , Q. Tang , B. Yu , B. Zhao , C. Mao , M. Wan , Adv. Mater. 2022, 34, 2206654. 10.1002/adma.202206654 36122571

[340] F. Peng , Y. Tu , Y. Men , J. C. M. van Hest , D. A. Wilson , Adv. Mater. 2017, 29, 1604996. 10.1002/adma.201604996 27891683

[341] E. Jambon‐Puillet , A. Testa , C. Lorenz , R. W. Style , A. A. Rebane , E. R. Dufresne , Nat. Commun. 2024, 15, 3919. 38724503 10.1038/s41467-024-47889-y PMC11082165

[342] M. Dindo , A. Bevilacqua , G. Soligo , V. Calabrese , A. Monti , A. Q. Shen , M. E. Rosti , P. Laurino , J. Am. Chem. Soc. 2024, 146, 15965. 38620052 10.1021/jacs.4c02823

[343] T. Xu , F. Soto , W. Gao , R. Dong , V. Garcia‐Gradilla , E. Magaña , X. Zhang , J. Wang , J. Am. Chem. Soc. 2015, 137, 2163. 25634724 10.1021/ja511012v

[344] M. Yang , Y. Zhang , F. Mou , C. Cao , L. Yu , Z. Li , J. Guan , Sci. Adv. 2023, 9, adk7251. 10.1126/sciadv.adk7251 PMC10686566 38019908

[345] C. Liu , J. Niu , T. Cui , J. Ren , X. Qu , J. Am. Chem. Soc. 2022, 144, 19611. 36240426 10.1021/jacs.2c09599

[346] T. Cui , S. Wu , Y. Sun , J. Ren , X. Qu , Nano Lett. 2020, 20, 7350. 32856923 10.1021/acs.nanolett.0c02767

[347] Q. Wang , S. Yang , L. Zhang , Nano‐Micro Lett. 2024, 16, 40. 10.1007/s40820-023-01261-9 PMC10689342 38032461

[348] L. Feuerstein , C. G. Biermann , Z. Xiao , C. Holm , J. Simmchen , J. Am. Chem. Soc. 2021, 143, 17015. 34523911 10.1021/jacs.1c06400

[349] W. Wang , T. Y. Chiang , D. Velegol , T. E. Mallouk , J. Am. Chem. Soc. 2013, 135, 10557. 23795959 10.1021/ja405135f

[350] B. Zhang , H. Pan , Z. Chen , T. Yin , M. Zheng , L. Cai , Sci. Adv. 2023, 9, adc8978. 10.1126/sciadv.adc8978 PMC9946363 36812317

[351] Y. Ye , F. Tong , S. Wang , J. Jiang , J. Gao , L. Liu , K. Liu , F. Wang , Z. Wang , J. Ou , B. Chen , D. A. Wilson , Y. Tu , F. Peng , Nano Lett. 2021, 21, 8086. 34559543 10.1021/acs.nanolett.1c02441

[352] C. Xu , Y. Liu , J. Li , P. Ning , Z. Shi , W. Zhang , Z. Li , R. Zhou , Y. Tong , Y. Li , C. Lv , Y. Shen , Q. Cheng , B. He , Y. Cheng , Adv. Mater. 2023, 35, 2204996. 10.1002/adma.202204996 36515124

